# Development of *mRuby2*-Transfected C3H10T1/2 Fibroblasts for Musculoskeletal Tissue Engineering

**DOI:** 10.1371/journal.pone.0139054

**Published:** 2015-09-25

**Authors:** Dai Fei Elmer Ker, Rashmi Sharma, Evelyna Tsi Hsin Wang, Yunzhi Peter Yang

**Affiliations:** 1 Department of Orthopaedic Surgery, Stanford University, Stanford, California, United States of America; 2 Department of Bioengineering, Stanford University, Stanford, California, United States of America; 3 Department of Material Science and Engineering, Stanford University, Stanford, California, United States of America; University of Torino, ITALY

## Abstract

Mouse C3H10T1/2 fibroblasts are multipotent, mesenchymal stem cell (MSC)-like progenitor cells that are widely used in musculoskeletal research. In this study, we have established a clonal population of C3H10T1/2 cells stably-transfected with *mRuby2*, an orange-red fluorescence reporter gene. Flow cytometry analysis and fluorescence imaging confirmed successful transfection of these cells. Cell counting studies showed that untransfected C3H10T1/2 cells and *mRuby2*-transfected C3H10T1/2 cells proliferated at similar rates. Adipogenic differentiation experiments demonstrated that untransfected C3H10T1/2 cells and *mRuby2*-transfected C3H10T1/2 cells stained positive for Oil Red O and showed increased expression of adipogenic genes including *adiponectin* and *lipoprotein lipase*. Chondrogenic differentiation experiments demonstrated that untransfected C3H10T1/2 cells and *mRuby2*-transfected C3H10T1/2 cells stained positive for Alcian Blue and showed increased expression of chondrogenic genes including *aggrecan*. Osteogenic differentiation experiments demonstrated that untransfected C3H10T1/2 cells and *mRuby2*-transfected C3H10T1/2 cells stained positive for alkaline phosphatase (ALP) as well as Alizarin Red and showed increased expression of osteogenic genes including *alp*, *ocn* and *osf-1*. When seeded on calcium phosphate-based ceramic scaffolds, *mRuby2*-transfected C3H10T1/2 cells maintained even fluorescence labeling and osteogenic differentiation. In summary, *mRuby2*-transfected C3H10T1/2 cells exhibit mRuby2 fluorescence and showed little-to-no difference in terms of cell proliferation and differentiation as untransfected C3H10T1/2 cells. These cells will be available from American Type Culture Collection (ATCC; CRL-3268™) and may be a valuable tool for preclinical studies.

## Introduction

The mouse C3H10T1/2 fibroblast cell line is an attractive, multipotent cell source for musculoskeletal research. Established in 1973, this cell line was derived from 14–17 day old C3H mouse embryos [1, 2] and shares similar characteristics with mesenchymal stem cells (MSCs) with respect to cell differentiation [3–27] and secretion of paracrine factors conducive to tissue regeneration [28–31]. As such, C3H10T1/2 cells are an ideal surrogate for studying the biology and translational application of MSCs.

For such studies, fluorescence labeling of cells is widely used as a technique to monitor and track cells *in vitro* and *in vivo*. Fluorescence cell labeling can either utilize short-term approaches such as incubation with fluorescent dyes and transient transfection of fluorescent reporter gene(s) or long-term approaches such as stable transfection of fluorescent reporter gene(s). Often, long-term methods are preferred since short-term methods utilizing fluorescent dyes and transient reporter gene(s) undergo dilution as cells divide, exhibiting a progressive loss in fluorescence signal. However, long-term approaches that involve stable transfection of fluorescent reporter gene(s) may result in undesired and unknown perturbations to cell behavior such as inability to undergo cell differentiation at high gene doses [32].

In this study, we report on the stable transfection of C3H10T1/2 with one of brightest monomeric orange-red fluorescent reporter genes to date, *mRuby2* [33], and compare the proliferation as well as differentiation ability of untransfected and stably-transfected C3H10T1/2 cells. Our results demonstrated that C3H10T1/2 cells stably transfected with *mRuby2* fluorescent reporter gene exhibited little-to-no change in cell proliferation as well as adipogenic, chondrogenic, and osteogenic differentiation. As such, the development of *mRuby2*-transfected C3H10T1/2 cells may serve as a useful tool for studying musculoskeletal biology and regeneration.

## Methods

### Cell culture

Multipotent mouse C3H10T1/2 cells (ATTC, Manassas, VA) were grown in Dulbecco’s Modified Eagle’s Media (DMEM; Life Technologies, Carlsbad, CA), 10% fetal bovine serum (FBS; Life Technologies, Carlsbad, CA) and 1% penicillin-streptomycin (PS; Life Technologies, Carlsbad, CA). All cells were kept at 37°C, 5% CO_2_ in a humidified incubator.

### Cloning and transfection

The *mRuby2* gene [33] (Kindly provided by Amy Lam and Michael Lin, Stanford University, CA, USA) was cloned into pVitro2-MCS-Blast plasmid (InvivoGen, San Diego, CA) to generate pVitro2-*mRuby2*-Blast plasmid, which was subsequently verified by DNA-sequencing (Elim Biopharmaceuticals Inc., Hayward, CA; Genbank Accession Number: BankIt1780092 mRuby2 KP236589). Cells were seeded at a density of 0.63 x 10^4^ cells/cm^2^ overnight and transfected using jetPRIME transfection reagent (PolyPlus, Berkeley, CA) according to the manufacturer’s instructions. Briefly, cells were seeded in a 6-well plate at 60–70% confluency and transfected with 2 μg of plasmid DNA. Stably-transfected cells were selected over a period of 10 days in DMEM, 10% FBS, 1% PS media containing 3 μg/mL blasticidin. Blasticidin-resistant cells were maintained in complete DMEM media post-selection.

### Dil labeling

C3H10T1/2 cells were labeled with 5 μM DiI dye (Life Technologies, Carlsbad, CA) for 20 min at 37°C according to the manufacturer’s instructions.

### Fabrication of calcium phosphate-based ceramics

Calcium phosphate-based ceramics (8 mm diameter, 2.5 mm height) were fabricated using a template casting technique as described previously [34].

### Flow cytometry

Cells stably transfected with pVitro2-*mRuby2*-Blast plasmid were sorted using a BD Aria II flow cytometer (BD Biosciences, Franklin Lakes, NJ) as individual clones into 96-well plates. These clones were subsequently expanded and assayed for differentiation potential as described below. Data were analyzed using Flowjo 9.7.5 (http://www.flowjo.com). In addition, these cells were deposited into ATCC (Manassas, VA) for public use.

### Cell doubling

Cells were seeded into five 24-well plates at a density of 0.26 x 10^4^ cells/cm^2^ overnight. The following day (Day 0), media were changed to DMEM, 10% FBS, 1% PS. Media were changed every 48 h. Cells were counted every 24 h using a Beckman Coulter Z2 Particle Counter (Beckman Coulter Inc., Pasadena, CA). Cell doubling times were calculated using R Studio (http://www.rstudio.com). For each sample, the period of exponential growth was manually determined by visual inspection and the log of cell counts was plotted against the period of exponential growth. The data corresponding to this period of exponential growth were fitted to a linear model and the slope (growth rate) was determined. Subsequently, the doubling time was determined by applying the formula (Doubling time = ln(2)/slope).

### Alcian Blue staining

Cells were seeded into 24-well plates at a density of 8 x 10^4^ cells/5 μL drops to generate micromass cultures. After 2 h (Day 0), media were changed to DMEM, 10% FBS, 1% PS (Control media) or StemPro Chondrogenic Differentiation Kit Media (Chondrogenic media, Life Technologies, Carlsbad, CA). Media were changed every 72 h. After 15 days of chondrogenic induction, cells were washed with PBS, fixed with 10% neutral buffered formalin for 30 min, stained with 1% Alcian Blue (in 1N HCl; Electron Microscopy Sciences, Hatfield, PA) for 30 min, rinsed three times with distilled water, air-dried and imaged using an inverted Zeiss AxioObserver Z1 microscope equipped with a color camera (Zeiss microimaging, Thornwood, NY).

### Alkaline phosphatase (ALP) staining

Cells were seeded into 24-well plates or our in-house manufactured calcium phosphate-based ceramics[34] at a density of 1.57 x 10^4^ cells/cm^2^ and 5.97 x 10^4^ cells/cm^2^ overnight, respectively. The following day (Day 0), media were changed to DMEM, 10% FBS, 1% PS (Without BMP-2) or DMEM, 10% FBS, 1% PS, 100 ng/mL BMP-2 (With BMP-2, Medtronic, Minneapolis, MN). Media were changed every 48 h. At the appropriate time point (6 or 10 days), cells were fixed for 1 min in 3.7% formaldehyde. ALP activity (Sigma Aldrich, St. Louis, MO) was detected according to the manufacturer’s instructions (Sigma Aldrich, St. Louis, MO). Samples were imaged using an inverted Zeiss AxioObserver Z1 microscope equipped with a color camera. Where necessary, the average pixel intensity was determined using the image histogram tool in Adobe Photoshop 8.0 (Adobe Systems, San Jose, CA) as previously described [35, 36].

### Alizarin Red staining

Cells were seeded into 24-well plates at a density of 1.57 x 10^4^ cells/cm^2^ overnight. The following day (Day 0), media were changed to DMEM, 10% FBS, 1% PS, 50 μg/mL ascorbic acid, 10 mM β-glycerophosphate (Control media) or DMEM, 10% FBS, 1% PS, 50 μg/mL ascorbic acid, 10 mM β-glycerophosphate, 100 ng/mL BMP-2 (Osteogenic media). Media were changed every 72 h. After 27 days, cells were washed with PBS, fixed with 10% neutral buffered formalin for 30 min, stained with 2% Alizarin Red stain (Electron Microscopy Sciences, Hatfield, PA) for 45 min, rinsed three times with distilled water, air-dried and imaged using an inverted Zeiss AxioObserver Z1 microscope equipped with a color camera.

To quantify Alizarin Red staining, 1 mL of extraction solvent (8% acetic acid, 20% methanol in water) was added to each well for 45 min. Standards were constructed using 0, 0.1, 0.5, 1, 2, 5, 10, 50, 100, 200, 500, 700 μg/mL Alizarin Red stain. Absorbance of standards and samples were read at 405 nm using a Tecan Infinite F50 spectrometer (Tecan Trading AG, Switzerland).

### Oil Red O staining

Cells were seeded into 24-well plates at a density of 1.57 x 10^4^ cells/cm^2^ overnight. The following day (Day 0), media were changed to DMEM, 10% FBS, 1% PS (Control media) or StemPro Adipogenic Differentiation Kit Media (Adipogenic media, Life Technologies, Carlsbad, CA). Media were changed every 72 h. After 15 days of adipogenic induction, cells were washed with PBS, fixed with 10% neutral buffered formalin for 30 min, stained with 0.5% Oil Red O (in 60% isopropanol; Electron Microscopy Sciences, Hatfield, PA) for 30 min, rinsed with 60% ispropanol, air-dried and imaged using an inverted Zeiss AxioObserver Z1 microscope equipped with a color camera.

### Non-competitive, semi-quantitative polymerase chain reaction (PCR)

Cells were seeded at similar cell densities in 6-well plates as described for Alcian Blue, Alizarin Red and Oil Red O staining experiments. At the appropriate timepoint(s), total RNA was harvested according to the manufacturer’s instructions (RNeasy Mini Kit; Qiagen, Valencia, CA). Reverse transcription into cDNA was performed according to the manufacturer’s instructions (Omniscript RT Kit; Qiagen, Valencia, CA). Semi-quantitative PCR was performed for target genes using Platinum blue mastermix as per manufacturer’s instructions (Life Technologies, Carlsbad, CA).

Adipogenic target genes included *adiponectin* (*adipoq*, forward primer: GGACAAGGCCGTTCTCTTCA, reverse primer: TGGGCTATGGGTAGTTGCAG), *lipoprotein lipase* (*lpl*, forward primer: TGAGGATGGCAAGCAACACA, reverse primer: GTTTGTCCAGTGTCAGCCAG) and *peroxisome proliferator activated receptor gamma* (*pparg*, forward primer: GGGCTGAGGAGAAGTCACAC, reverse primer: ACAGACTCGGCACTCAATGG). Chondrogenic target genes included *aggrecan* (*acan*, forward primer: CTTACCCTGAGGCTGGTGTG, reverse primer: ACATTGCTCCTGGTCTGCAA), *collagen2a1* (*col2a1*, forward primer: TCGACATGTCAGCCTTTGCT, reverse primer: AATGTCCATGGGTGCGATGT) and *sex determining region Y-box 9* (*sox9*, forward primer: CCAGCAAGAACAAGCCACAC, reverse primer: CTCATGCCGGAGGAGGAATG). Osteogenic target genes included *alkaline phosphatase* (*alp*, forward primer: GGGCAATGAGGTCACATCCA, reverse primer: AGCCTTTGGGGTTCTTGGTC), *osteoblast specific factor-1* (*osf-1*, forward primer: CTCTATTTCCCTCCCCGGCAG, reverse primer: ACGCACACACTCCACTGCCAT) and *osteocalcin/bone gamma-carboxyglutamic acid-containing protein* (*ocn*, forward primer: GGTCAATCCCCATGTCCAGG, reverse primer: GTTTGGCTTTAGGGCAGCAC). Expression of target genes were normalized to *glyceraldehyde-3 phosphate dehydrogenase* (*gapdh*, forward primer: GGTTGTCTCCTGCGACTTCA, reverse primer: TAGGGCCTCTCTTGCTCAGT) internal control.

PCR products and gene expression levels were analyzed by agarose gel electrophoresis and Adobe Photoshop 8.0, respectively.

### Statistical analysis

To determine significance among treatment groups, various statistical tests were performed. For flow cytometry analysis, univariate population comparison was performed using a Chi squared test and Overton subtraction using FlowJo 9.7.5 software. For all other experiments, either a t-test (Two treatment groups) or one-way analysis of variance followed by Tukey’s Honestly Significant Difference post hoc test (More than two treatment groups) was performed using SYSTAT 12 software (Systat Software Inc., Richmond, CA). A *p* value ≤ 0.05 was considered statistically significant.

## Results

### Transfection of mRuby2 fluorescent reporter gene

**Fig 1:**
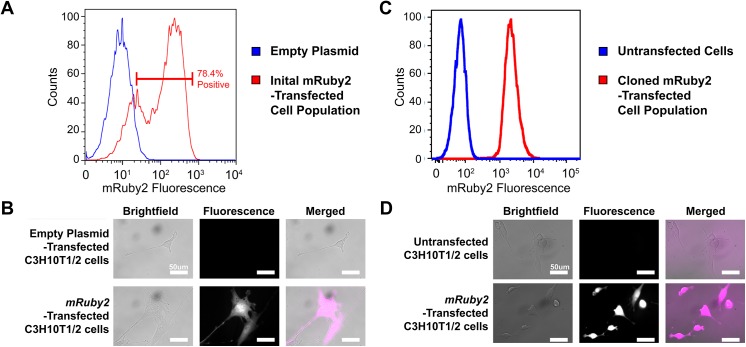
Stable Transfection of C3H10T1/2 Cells with empty plasmid or *mRuby2* Fluorescence Reporter Gene. **A.** Flow cytometry analysis of mRuby2 fluorescence in C3H10T1/2 cells transfected with empty plasmid (Blue) and *mRuby2* fluorescence reporter gene (Red). Data shown represent initial transfected cell populations prior to cell cloning. Majority of *mRuby2*-transfected C3H10T1/2 cells (78.4%) exhibited increased mRuby2 fluorescence over control. **B.** Brightfield and fluorescence images of C3H10T1/2 cells transfected with empty plasmid and cloned *mRuby2*-transfected C3H10T1/2 cells (n = 9). Cloned *mRuby2*-transfected C3H10T1/2 cells exhibited increased mRuby2 fluorescence over control. **C.** Flow cytometry analysis of mRuby2 fluorescence in untransfected C3H10T1/2 cells (Blue) and C3H10T1/2 cells transfected with *mRuby2* fluorescence reporter gene (Red). Data shown represent a stably-transfected clonal cell population after approximately 2 months culture. *mRuby2*-transfected C3H10T1/2 cells exhibited increased mRuby2 fluorescence over control. **D.** Brightfield and fluorescence images of untransfected C3H10T1/2 cells and cloned *mRuby2*-transfected C3H10T1/2 cells after approximately 2 months culture (n = 3). Cloned *mRuby2*-transfected C3H10T1/2 cells exhibited increased mRuby2 fluorescence over control. Scale bars 50 μm.

C3H10T1/2 cells stably-transfected with *mRuby2* fluorescence reporter gene were analyzed by flow cytometry and fluorescence imaging (**Fig 1**). 78.4% of *mRuby2*-transfected C3H10T1/2 cells exhibited increased mRuby2 fluorescence over control (**Fig 1A,** *p* < 0.01). From this population, several stably-transfected, bright mRuby2-positive clones were isolated and expanded for subsequent studies. Fluorescence imaging showed that cloned *mRuby2*-transfected C3H10T1/2 cells retained mRuby2 expression (**Fig 1B**). After approximately 2 months of culture, cloned *mRuby2*-transfected C3H10T1/2 cells still retained mRuby2 expression as determined by flow cytometry (**Fig 1C** *p* < 0.01) and fluorescence imaging (**Fig 1D**).

### Cell proliferation

**Fig 2:**
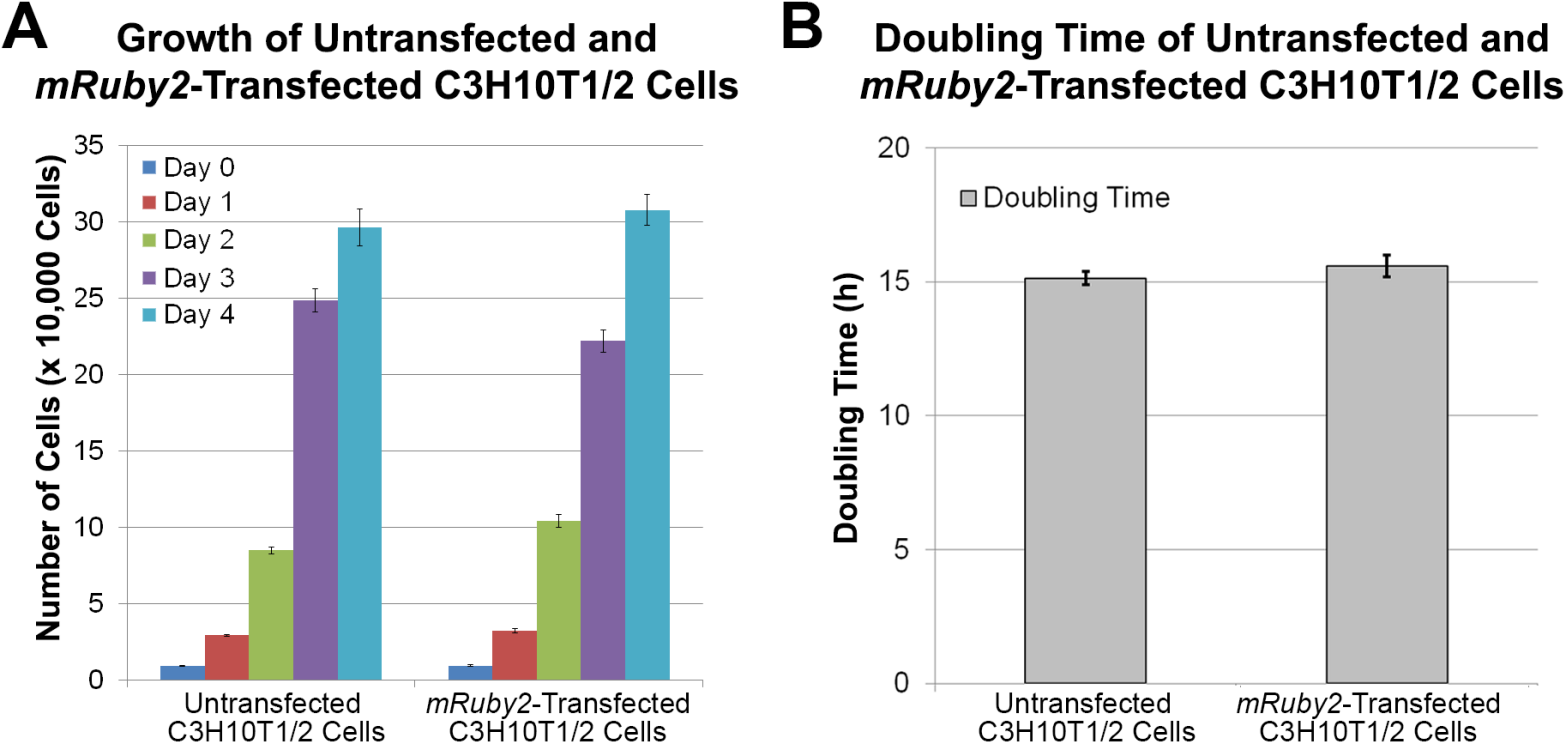
Proliferation of Untransfected C3H10T1/2 Cells and Cloned *mRuby2*-Transfected C3H10T1/2 Cells. **A.** Growth of untransfected C3H10T1/2 cells and cloned *mRuby2*-transfected C3H10T1/2 cells (n = 9). No differences were observed. **B.** Doubling time of untransfected C3H10T1/2 cells and cloned *mRuby2*-transfected C3H10T1/2 cells (n = 9). No differences were observed. Error bars indicate standard error of mean.

The proliferation capabilities of untransfected C3H10T1/2 cells and *mRuby2*-transfected C3H10T1/2 cells were determined by cell counting (**Fig 2**). Both groups exhibited no differences in both cell counts (**Fig 2A**) and cell doubling times (**Fig 2B**, *p* = 0.356). As such, transfection of *mRuby2* fluorescence reporter gene did not affect C3H10T1/2 cell proliferation under normal culture conditions.

### Adipogenic differentiation

**Fig 3:**
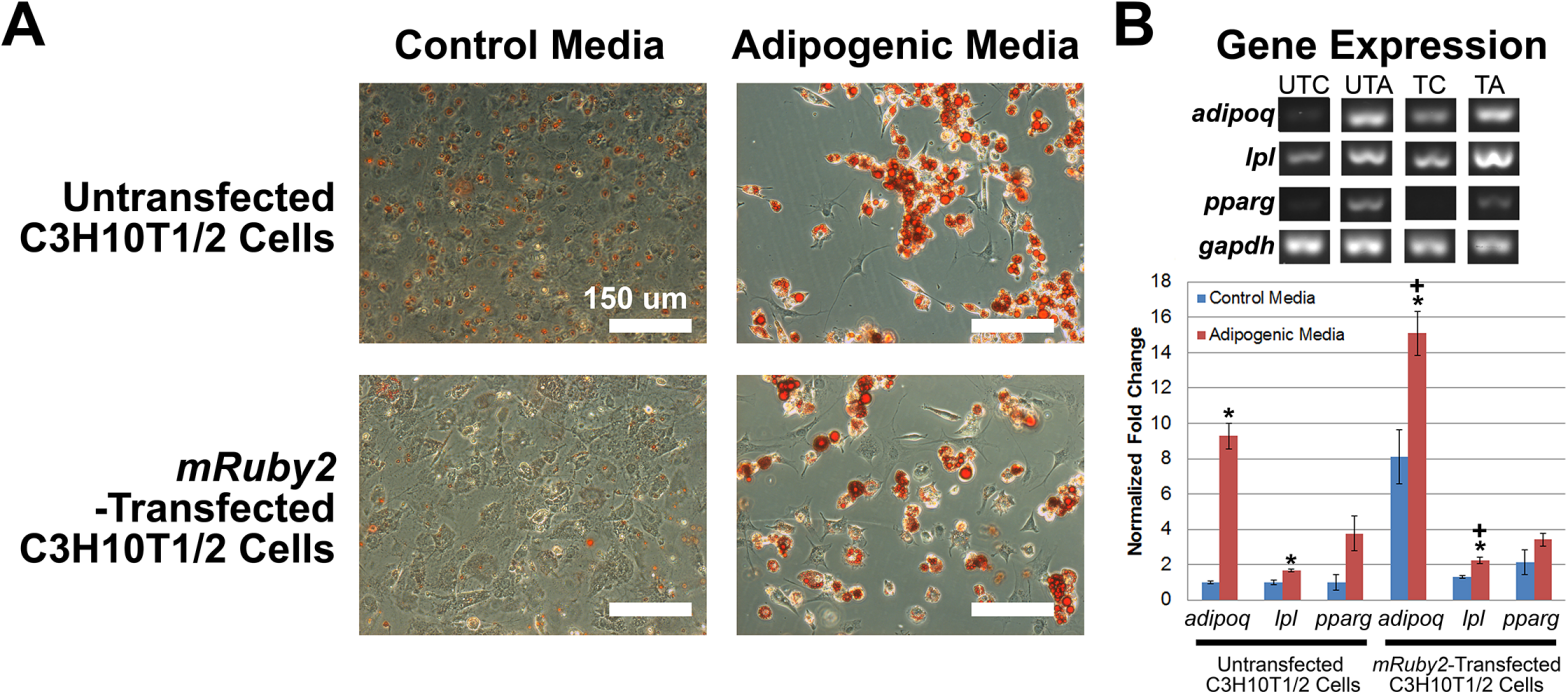
Adipogenic Differentiation of Untransfected C3H10T1/2 Cells and Cloned *mRuby2*-Transfected C3H10T1/2 Cells. **A.** Oil Red O staining of untransfected C3H10T1/2 cells and cloned *mRuby2*-transfected C3H10T1/2 cells after 15 days in control and adipogenic media (n = 9). When cultured in adipogenic media, both cell types exhibited strong, positive staining for lipid droplets (Red). Scale bars 150 μm. **B.** Non-competitive, semi-quantitative PCR of adipogenic genes (*adiponectin*: *adipoq*, *lipoprotein lipase*: *lpl* and *peroxisome proliferator-activated receptor gamma*: *pparg*) after 15 days in control and adipogenic media (n = 3). Data were normalized to *glyceraldehyde dehydrogenase* (*gapdh*) housekeeping gene. When cultured in adipogenic media, both cell types exhibited increased expression of *adipoq* and *lpl* but not *pparg*. UTC, untransfected control. UTA, untransfected adipogenic. TC, transfected control. TA, transfected adipogenic. *, statistically significant (p ≤ 0.05) when compared to its respective control. +, statistically significant (p ≤ 0.05) when compared to untransfected C3H10T1/2 cells in control media. Error bars indicate standard error of mean.

The adipogenic capabilities of untransfected C3H10T1/2 cells and *mRuby2*-transfected C3H10T1/2 cells were determined by Oil Red O staining for lipid droplets and non-competitive, semi-quantitative PCR for adipogenic gene expression (**Fig 3**). After 15 days, cells in both adipogenic groups exhibited a rounded morphology with intracellular accumulation of lipid droplets that stained positive for Oil Red O (**Fig 3A**) while sporadic Oil Red O staining was observed in both control groups (**Fig 3A**). For gene expression studies, untransfected C3H10T1/2 cells and *mRuby2*-transfected C3H10T1/2 cells exhibited similar trends (**Fig 3B**). After 15 days, cells in both adipogenic groups upregulated expression of *adiponectin* (*adipoq*) relative to its respective control (**Fig 3B**, *p* = 0.002 for C3H10T1/2 cells and *p* = 0.006 for *mRuby2*-transfected C3H10T1/2 cells). Similarly, cells in both adipogenic groups upregulated expression of *lipoprotein lipase* (*lpl*) relative to its respective control (**Fig 3B**, *p* = 0.031 for C3H10T1/2 cells and *p* = 0.012 for *mRuby2*-transfected C3H10T1/2 cells). In addition, expression of *adipoq* and *lpl* in *mRuby2*-transfected C3H10T1/2 cells in the adipogenic group were also increased compared to untransfected C3H10T1/2 cells in the control group (*p* < 0.001 for *adipoq* and *p* = 0.001 for *lpl*). Expression of *peroxisome proliferator-activated receptor gamma* (*pparg*) remained unchanged relative to its respective control (**Fig 3B**, *p* = 0.115 for C3H10T1/2 cells and *p* = 0.349 for *mRuby2*-transfected C3H10T1/2 cells). As such, transfection of *mRuby2* fluorescence reporter gene did not affect C3H10T1/2 adipogenic differentiation.

### Chondrogenic differentiation

**Fig 4:**
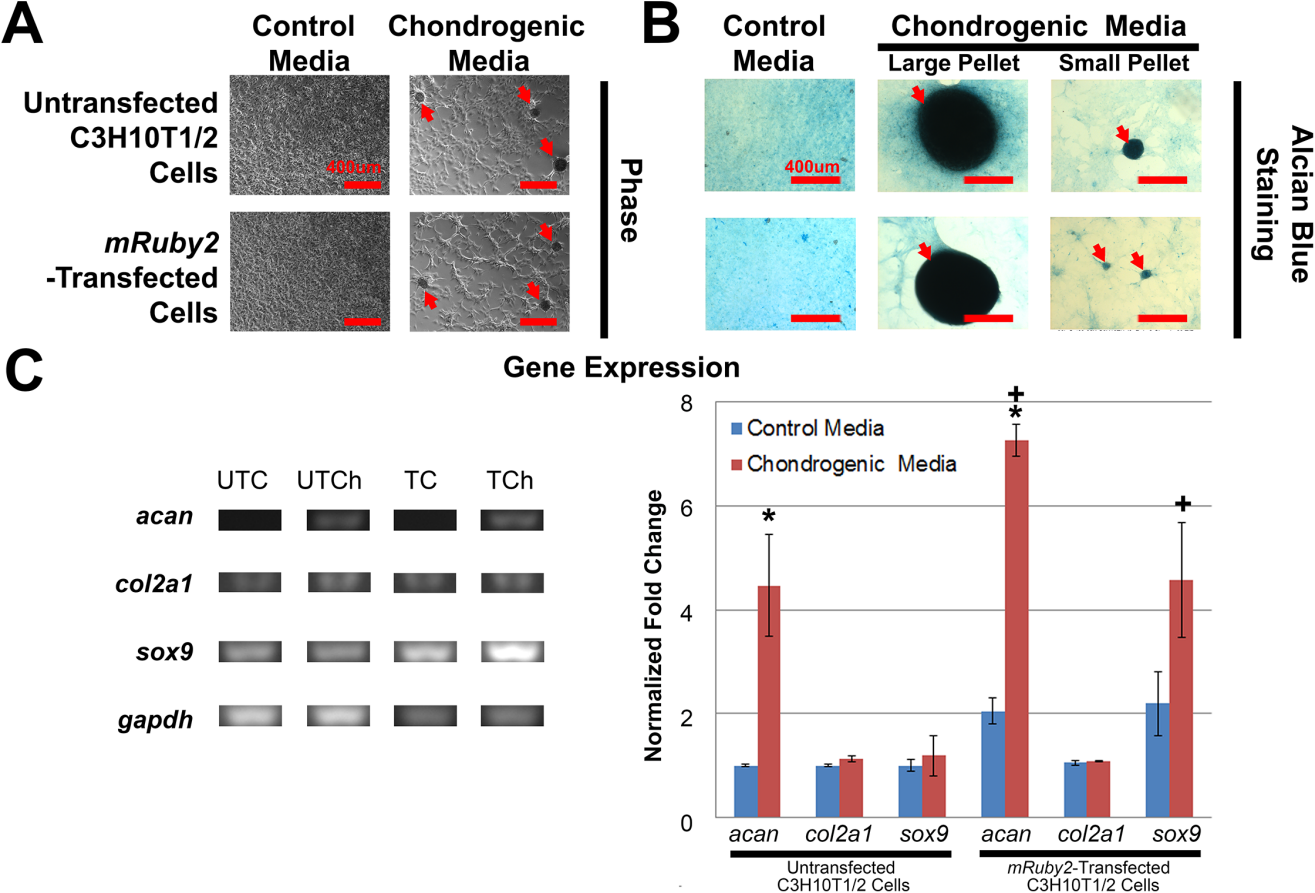
Chondrogenic Differentiation of Untransfected C3H10T1/2 Cells and Cloned *mRuby2*-Transfected C3H10T1/2 Cells. **A.** Phase-contrast images of untransfected C3H10T1/2 cells and cloned *mRuby2*-transfected C3H10T1/2 cells after 15 days in control and chondrogenic media (n = 9). When cultured in chondrogenic media, both cell types formed chondrogenic pellets as indicated by red arrows. Scale bars 400 μm. **B.** Alcian Blue staining of untransfected C3H10T1/2 cells and cloned *mRuby2*-transfected C3H10T1/2 cells after 15 days in control and chondrogenic media (n = 9). When cultured in chondrogenic media, both cell types formed chondrogenic pellets that exhibited strong, positive staining (Blue) for proteoglycans as indicated by red arrows. Scale bars 400 μm. **C.** Non-competitive, semi-quantitative PCR of chondrogenic genes (*aggrecan*: *acan*, *collagen2a1*: *col2a1* and *sex determining region Y-box 9*: *sox9*) after 15 days in control and chondrogenic media (n = 3). Data were normalized to *glyceraldehyde dehydrogenase* (*gapdh*) housekeeping gene. When cultured in chrondrogenic media, both cell types exhibited increased expression of *acan* but not *sox9* and *col2a1*. UTC, untransfected control. UTCh, untransfected chondrogenic. TC, transfected control. TCh, transfected chondrogenic. *, statistically significant (p ≤ 0.05) when compared to its respective control. +, statistically significant (p ≤ 0.05) when compared to untransfected C3H10T1/2 cells in control media. Error bars indicate standard error of mean.

The chondrogenic capabilities of untransfected C3H10T1/2 cells and *mRuby2*-transfected C3H10T1/2 cells were determined by Alcian Blue staining for proteoglycans and non-competitive, semi-quantitative PCR for chondrogenic gene expression (**Fig 4**). After 15 days, cells in both chondrogenic groups formed round structures reminiscent of chondrogenic pellets (**Fig 4A and 4B**) that stained positive for Alcian Blue (**Fig 4B**) while sporadic Alcian Blue staining was observed in both control groups (**Fig 4B**). For gene expression studies, untransfected C3H10T1/2 cells and *mRuby2*-transfected C3H10T1/2 cells exhibited similar trends (**Fig 4C**). After 15 days, cells in both chondrogenic groups upregulated expression of *aggrecan* (*acan*) relative to its respective control (**Fig 4C**, *p* = 0.008 for C3H10T1/2 cells and *p* = 0.001 for *mRuby2*-transfected C3H10T1/2 cells). In addition, expression of *acan* in *mRuby2*-transfected C3H10T1/2 cells in the chondrogenic media group was also increased compared to untransfected C3H10T1/2 cells in the control group (*p* < 0.001). Expression of *collagen2a1* (*col2a1*) remained unchanged relative to its respective control (**Fig 4C**, *p* = 0.198 for C3H10T1/2 cells and *p* = 0.914 for *mRuby2*-transfected C3H10T1/2 cells). Expression of *sex determining region Y-box 9* (*sox9*) remained unchanged relative to its respective control (**Fig 4C**, *p* = 0.997 for C3H10T1/2 cells and *p* = 0.128 for *mRuby2*-transfected C3H10T1/2 cells). However, expression of *sox9* in *mRuby2*-transfected C3H10T1/2 cells in the chondrogenic group was increased compared to untransfected C3H10T1/2 cells in the control group (*p* = 0.022). As such, transfection of *mRuby2* fluorescence reporter gene did not affect C3H10T1/2 chondrogenic differentiation.

### Osteogenic differentiation

**Fig 5:**
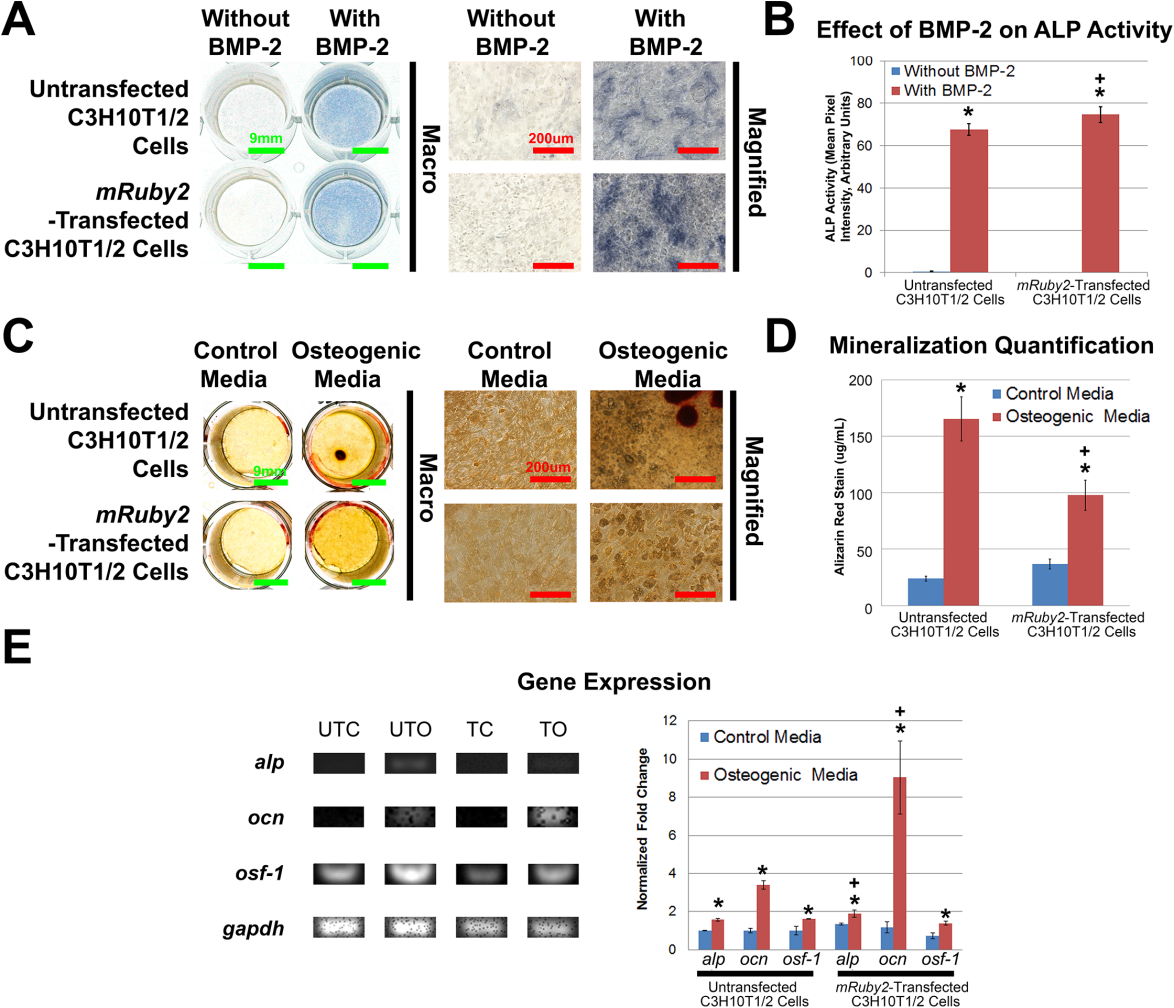
Osteogenic Differentiation of Untransfected C3H10T1/2 Cells and Cloned *mRuby2*-Transfected C3H10T1/2 Cells. **A.** ALP staining of untransfected C3H10T1/2 cells and cloned *mRuby2*-transfected C3H10T1/2 cells after 6 days in control and BMP-2 containing media (n = 9). When cultured in BMP-2 containing media, both cell types exhibited increased ALP staining (Blue). Scale bars 9 mm and 200 μm as indicated. **B.** Quantification of ALP staining of untransfected C3H10T1/2 cells and cloned *mRuby2*-transfected C3H10T1/2 cells after 6 days in control and BMP-2 containing media (n = 9). When cultured in BMP-2 containing media, both cell types exhibited increased ALP staining. **C.** Alizarin Red staining of untransfected C3H10T1/2 cells and cloned *mRuby2*-transfected C3H10T1/2 cells after 27 days in control and osteogenic media (n = 11). When cultured in osteogenic media, both cell types exhibited increased Alizarin Red staining (Red). Scale bars 9 mm and 200 μm as indicated. **D.** Quantification of Alizarin Red staining of untransfected C3H10T1/2 cells and cloned *mRuby2*-transfected C3H10T1/2 cells after 27 days in control and osteogenic media (n = 11). When cultured in osteogenic media, both cell types exhibited increased Alizarin Red staining. **E.** Non-competitive, semi-quantitative PCR of osteogenic genes (*alkaline phosphatase*: *alp*, *osteocalcin*: *ocn and osteoblast specific factor-1*: *osf-1*) after 27 days in control and osteogenic media (n = 3). Data were normalized to *glyceraldehyde dehydrogenase* (*gapdh*) housekeeping gene. When cultured in osteogenic media, both cell types exhibited increased expression of *alp*, *ocn and osf-1*. UTC, untransfected control. UTO, untransfected osteogenic. TC, transfected control. TO, transfected osteogenic. *, statistically significant (p ≤ 0.05) when compared to its respective control. +, statistically significant (p ≤ 0.05) when compared to untransfected C3H10T1/2 cells in control media. Error bars indicate standard error of mean.

The osteogenic capabilities of untransfected C3H10T1/2 cells and *mRuby2*-transfected C3H10T1/2 cells were determined by ALP staining for early osteoblast differentiation, Alizarin Red staining for calcium mineralization and non-competitive, semi-quantitative PCR for osteogenic gene expression (**Fig 5**). After 6 days, cells in both BMP-2-treated groups exhibited similar levels of ALP activity (**Fig 5A and 5B**, *p* < 0.001 for C3H10T1/2 cells and *p* < 0.001 for *mRuby2*-transfected C3H10T1/2 cells). In addition, ALP staining of *mRuby2*-transfected C3H10T1/2 cells in the BMP-2-treated group was also increased compared to untransfected C3H10T1/2 cells in the control group (*p* < 0.001). After 27 days, cells in both osteogenic groups exhibited similar levels of Alizarin Red staining (**Fig 5C and 5D**) while sporadic Alizarin Red staining was observed in both control groups (**Fig 5C and 5D**). Quantification of Alizarin Red staining showed that untransfected control, untransfected osteogenic, transfected control and transfected osteogenic groups contained a mean of 23.9 ± 2.1, 165.3 ± 19.5, 36.7 ± 4.4 and 97.7 ± 13.2 μg/mL Alizarin Red per well, respectively (**Fig 5D**). Cells in both osteogenic groups showed increased Alizarin Red staining relative to their respective control (**Fig 5D**, *p* < 0.001 for C3H10T1/2 cells and *p* = 0.005 for *mRuby2*-transfected C3H10T1/2 cells). In addition, Alizarin Red staining of *mRuby2*-transfected C3H10T1/2 cells in the osteogenic group was also increased compared to untransfected C3H10T1/2 cells in the control group (*p* = 0.001). For gene expression studies, untransfected C3H10T1/2 cells and *mRuby2*-transfected C3H10T1/2 cells exhibited similar trends (**Fig 5E**). After 27 days, cells in both osteogenic groups upregulated expression of *alp* relative to its respective control (**Fig 5E**, *p* = 0.020 for C3H10T1/2 cells and *p* = 0.031 for *mRuby2*-transfected C3H10T1/2 cells). Similarly, cells in both osteogenic groups upregulated expression of *osteocalcin* (*ocn*) relative to its respective control (**Fig 5E**, *p* = 0.032 for C3H10T1/2 cells and *p* < 0.001 for *mRuby2*-transfected C3H10T1/2 cells). In addition, expression of *alp* and *ocn* in *mRuby2*-transfected C3H10T1/2 cells in the osteogenic group were also increased compared to untransfected C3H10T1/2 cells in the control group (*p* = 0.002 for *alp* and *p* < 0.001 for *ocn*). Furthermore, cells in both osteogenic groups upregulated expression of *osteoblast specific factor-1* (*osf-1*) relative to its respective control (**Fig 5E**, *p* = 0.045 for C3H10T1/2 cells and *p* = 0.022 for *mRuby2*-transfected C3H10T1/2 cells). As such, transfection of *mRuby2* fluorescence reporter gene did not affect C3H10T1/2 osteogenic differentiation.

### Potential use in tissue engineering studies

**Fig 6:**
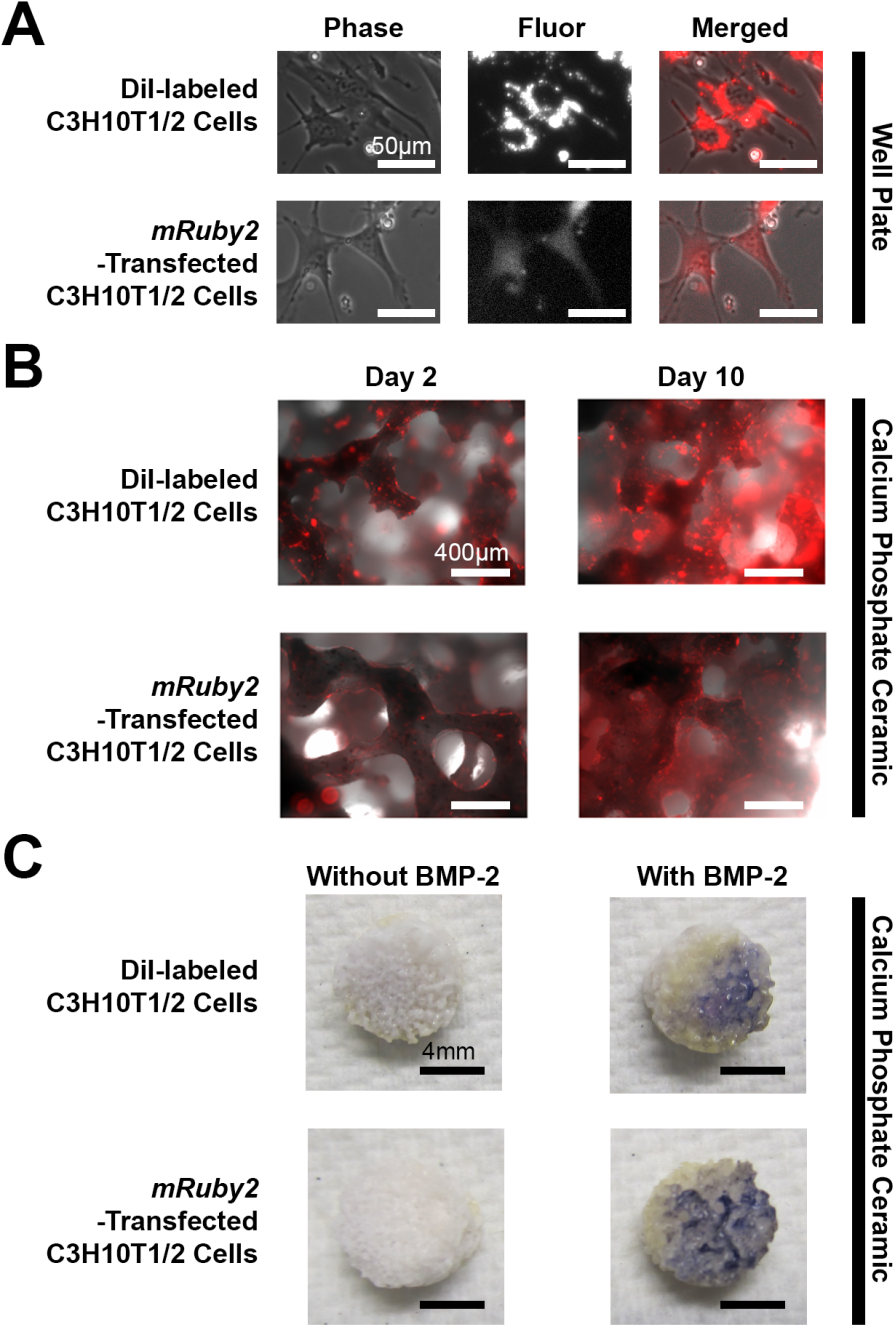
Osteogenic Differentiation of DiI-labeled C3H10T1/2 Cells and Cloned *mRuby2*-Transfected C3H10T1/2 Cells on Calcium Phosphate Ceramics. **A.** Phase-contrast and fluorescence images of C3H10T1/2 cells labeled with 5 μM Dil dye and cloned *mRuby2*-transfected C3H10T1/2 cells in tissue culture-treated well plates (n = 6). Cloned *mRuby2*-transfected C3H10T1/2 cells exhibited even labeling throughout the cells whereas DiI-labled C3H10T1/2 cells exhibited peri-nuclear labeling. Scale bars 50 μm. **B.** Phase-contrast and fluorescence images of C3H10T1/2 cells labeled with 5 μM Dil dye and cloned *mRuby2*-transfected C3H10T1/2 cells in calcium phosphate-based ceramics (n = 6). Both cell types exhibited fluorescence over a period of 10 days. Scale bars 400 μm. **c.** ALP staining of C3H10T1/2 cells labeled with 5 μM Dil dye and cloned *mRuby2*-transfected C3H10T1/2 cells after 10 days in control and BMP-2 containing media (n = 3). When cultured in BMP-2 containing media, both cell types exhibited increased ALP staining (Blue). Scale bars 4 mm (n = 3).

The potential use of *mRuby2*-transfected C3H10T1/2 cells for long-term labeling in musculoskeletal tissue engineering studies was determined by fluorescence imaging in tissue culture-treated well plates and calcium phosphate-based ceramics as well as ALP staining in calcium phosphate-based ceramics (**Fig 6**). After 2 days post-seeding, DiI-labeled C3H10T12 cells exhibited peri-nuclear staining whereas *mRuby2*-transfected C3H10T1/2 cells remained labeled throughout the cell (**Fig 6A**). Both labeled cell types could be visualized for at least 10 days post-seeding on calcium phosphate-based ceramics (**Fig 6B**). Also, both cell types in the BMP-2-treated groups exhibited increased ALP staining compared to their respective control groups (**Fig 6C**).

## Discussion

The aim of this study was to develop C3H10T1/2 cells stably-transfected with mRuby2 [33], an orange-red fluorescent reporter gene. Our studies show that untransfected C3H10T1/2 cells and *mRuby2-*transfected C3H10T1/2 cells did not exhibit any stark differences in terms of cell proliferation (**Fig 2**) as well as adipogenic, chondrogenic and osteogenic differentiation (**Figs 3**, **4**, **5** and **6**), indicating that these MSC-like cells may serve as a useful tool for studying musculoskeletal biology and regeneration.

As previously mentioned, MSCs are self-renewing multipotent stem cells that can differentiate into cells of the musculoskeletal system and secrete paracrine factors to promote tissue regeneration [3–5, 8–11, 13, 14, 16–20, 22, 24, 26–28, 30, 37–39]. While their origin is still subject to ongoing investigations, some studies have indicated that MSCs are derived from pericyte-like cells [3, 40] and are associated with blood vessels throughout the body in tissues such as muscle [5]. As a result, these cells can be isolated from multiple tissues including adipose[27], amniotic fluid [13], bone marrow [10, 20], cord blood [9], teeth [18], heart [4], neuroglia [16], placenta [14], synovium [8], skeletal muscle [5, 26] as well as skin [26]. In addition to their broad availability, MSCs can differentiate into cells of adipose [3, 20], bone [3, 20], cartilage [3, 20], heart [17], liver [19], neural [22], skeletal muscle [24] and tendon [11] lineages. Given their diverse source and ability to differentiate into a multitude of cells, many clinical trials involving MSCs have been conducted [41].

Despite these promising features, MSCs are a heterogeneous cell population with widely varied phenotypic behavior that hinders both their study and clinical translation [42]. This heterogeneous behavior is a result of donor-to-donor variability, their derivation from numerous tissue sources as well as the varied approaches used to isolate them including tissue culture plate adherence [10, 20, 43] and different combinations of cell surface markers [20, 41]. For example, adipose-derived rabbit MSCs show reduced osteogenic differentiation potential when compared to bone marrow- and muscle-derived MSCs [42] while adipose-derived MSCs and microvascular flaps obtained from elderly and diabetic patients demonstrate a reduced ability to secrete angiogenic factors and undergo smooth muscle and angiogenic differentiation [44, 45]. Furthermore, owing to limitations in cell culture techniques, MSCs exhibit a progressive loss of its stem cell properties with increased passaging [46]. In contrast, C3H10T1/2 cells are a widely available immortalized cell line that exhibit reproducible behavior. While the heterogenous and widely varied behavior of MSCs must be addressed for clinical translation, C3H10T1/2 cells provide a viable alternative and reproducible means for studying the biology and translational application of MSCs within the context of preclinical studies.

As previously mentioned, C3H10T1/2 cells have some features that are MSC-like, including the ability to differentiate into cells of musculoskeletal lineage and the ability to secrete paracrine factors to promote tissue regeneration. For example, C3H10T1/2 cells have been reported to differentiate into a variety of musculoskeletal cells of adipose [6, 12, 25], bone [15, 25], cartilage [21, 25], muscle [7, 23] and tendon [11, 35, 36] lineages. In addition, C3H10T1/2 cells also secrete paracrine factors that mediate interactions with endothelial cells to increase as well as participate in blood vessel formation [29, 31]. Indeed, comparable cell differentiation capability between MSCs and C3H10T1/2 cells has been shown with both cell types capable of differentiation into various mesenchymal lineages although some minor differences in adipogenic differentiation have been reported [47].

MSC identity is often ascertained through differentiation of cells into three mesenchymal lineages, adipose, cartilage and bone, since this feat cannot be accomplished by differentiated cells such as skin fibroblasts [20]. In our study, we compared the differentiation ability of untransfected C3H10T1/2 cells and *mRuby2*-transfected C3H10T1/2 cells (**Figs 3**, **4** and **5**). Differentiation of cells into adipocytes is confirmed by positive staining of neutral lipid droplets via Oil Red O staining [20] and expression of adipogenic genes such as *adipoq*, *lpl* and *pparg*. In our study, untransfected C3H10T1/2 cells and *mRuby2*-transfected C3H10T1/2 cells in both adipogenic groups stained positive for Oil Red O staining and showed increased expression of *adipoq* and *lpl* relative to their respective control (**Fig 3**). However, the lack of increased expression for *pparg* was unexpected. This may be explained by either the low sample number used for gene expression studies (n = 3) or the use of a single media for adipogenic differentiation. Some adipogenic studies utilized a combination of proliferation and adipogenic differentiation media that are alternatively fed to cells every few days to stimulate growth and differentiation [48]. This procedure helps to increase the number of adipogenic cells since cell proliferation often decreases as cells differentiate. Indeed, untransfected C3H10T1/2 cells and *mRuby2*-transfected C3H10T1/2 cells in both adipogenic groups were sparsely populated relative to their control groups and contained a mixture of adipocytes and non-adipocytes (**Fig 3A**), which may result in lowered expression of some adipogenic genes [48]. Differentiation of cells into chondrocytes is confirmed by positive staining of cartilage extracellular matrix rich in proteoglycans via Alcian Blue staining [49] and expression of chondrogenic genes such as *acan* and *sox9*. In our study, untransfected C3H10T1/2 cells and *mRuby2*-transfected C3H10T1/2 cells in both chondrogenic groups stained positive for Alcian Blue staining and showed increased expression of *acan* relative to their respective control (**Fig 2**). However, the lack of increased expression for *sox9* and *col2a1* were unexpected. This may be explained by either the low sample number used for gene expression studies (n = 3) or the use of micromass cultures for chondrogenic differentiation. Some chondrogenic studies utilize a micropellet format where cells are centrifuged into a pellet and cultured with chondrogenic media, creating a three dimensional culture environment that is crucial for chondrogenesis [50]. This three dimensional environment may be less well reproduced in the micro mass culture format of our study where cells are seeded at high density within a small two dimensional area. Indeed, untransfected C3H10T1/2 cells and *mRuby2*-transfected C3H10T1/2 cells in both chondrogenic groups contained a mixture of chondrocytes and non-chondrocytes (**Fig 4**), which may result in lower expression of some chondrogenic genes. Differentiation of cells into osteoblasts is confirmed by positive staining of ALP staining, formation of calcium deposits within bone extracellular matrix via Alizarin Red staining [49] and expression of osteogenic genes such as *alp* and *ocn*. In our study, untransfected C3H10T1/2 cells and *mRuby2*-transfected C3H10T1/2 cells in both BMP-2-treated and osteogenic groups showed increased staining for ALP activity, Alizarin Red staining and increased expression of *alp*, *ocn* and *osf-1* relative to their respective control (**Fig 5**). In addition, *mRuby2*-transfected C3H10T1/2 cells exhibited increased ALP staining on calcium phosphate-based ceramics [34], indicating their potential use in musculoskeletal tissue engineering studies (**Fig 6**).

Fluorescence-based cell tracking is a powerful method for visualizing biological processes *in vitro* and *in vivo*, requiring careful consideration of desired fluorescent probe properties and the approach used for implementation. Such criteria include but are not limited to: 1) fluorescent dyes or proteins that are specific for the cell-of-interest, 2) fluorescent dyes or proteins that can be monitored over the desired duration, 3) fluorescent dyes or proteins that can be adequately detected while accommodating co-staining with other reagents and 4) fluorescent dyes or proteins that are non-toxic and do not hinder cellular processes.

First, *in vitro* co-cultures studies and *in vivo* pre-clinical studies that involve transplantation of stem cells often require fluorescent dyes or proteins capable of distinguishing different cell types. While *in vitro* co-cultured cells can be easily distinguished through immunostaining, determining *in vivo* engraftment and differentiation of transplanted stem cells including MSCs are more complex since the *in vivo* environment is comprised of a complex milieu of cell types. This complexity arises because several commonly-used MSC markers such as CD105, CD73, CD90 and STRO-1 can be found in non-MSCs including vascular endothelial cells [51]. Also, these cell surface markers may change following cell isolation [51]. Stable-transfection of cells with a fluorescent reporter gene such as *mRuby2* circumvents these limitations as cells-of-interest and their progeny are fluorescently-labeled.

Second, cell differentiation experiments often require long durations (Up to four weeks for bone mineralization studies) which is difficult to monitor with transient fluorescent dyes and reporter gene(s) as their fluorescence signal is progressively diluted with cell division [51]. This is evident with C3H10T1/2 cells labelled with DiI, a spectrally similar (orange-red), long-term cell membrane dye where even labeling was observed immediately post seeding (**Data not shown**) but only peri-nuclear staining of cells was observed from 2 days post-seeding onwards (**Fig 6**). Such uneven long-term cell membrane-labeling poses a potential complicating factor in that the membrane of labeled, dead cells may be absorbed by host tissue, resulting in misinterpretation of cell engraftment and differentiation [51]. This scenario poses fewer issues for *mRuby2*-transfected C3H10T1/2 cells since they exhibit even labeling throughout the cell (**Fig 6**).

Third, fluorescent dyes or proteins must be sufficiently bright to differentiate from background and be amenable to co-staining with other reagents to glean additional and complementary information. In this regard, *mRuby2* fluorescent reporter gene is one of the brightest orange-red fluorescence protein to date [33] (Excitation max: 559 nm, emission max: 600 nm) and allows for multi-channel imaging when combined with other more commonly-used variants of fluorescent dyes and proteins [33]. In addition, orange-red fluorescent proteins are noteworthy since cells and tissues including bone predominantly exhibit green autofluorescence [51, 52].

Lastly, the introduction of these fluorescent dyes or proteins should not result in any artifacts that compromise cellular processes such as growth or differentiation. When transfected into HeLa cells, *mRuby* fluorescent reporter gene, the predecessor of *mRuby2*, exhibited punctate fluorescent labeling resembling lyosomal localization as well as 10-fold higher cytotoxicity than enhanced green fluorescent protein [53]. This lyosomal localization and cytotoxicity was not observed when its successor, *mRuby2* was transfected into C3H10T1/2 cells (**Fig 1**). While the basis for these observations and differences remain unknown, it is speculated that high levels of mRuby fluorescent protein in HeLa cells caused protein aggregation, resulting in subsequent lysosomal localization and cytotoxicity [54, 55]. Related to this, high gene dosage has also been shown to change cell behavior and compromise a cell’s ability to undergo cell differentiation. For example, viral transduction of human umbilical vein endothelial cells with green fluorescent protein at high multiplicity of infection has been reported to reduce angiogenic differentiation, resulting in an inability to form vascular sprouts [32]. In contrast, *mruby2-*transfected C3H10T1/2 cells exhibited similar rates of proliferation (**Fig 2**) and differentiation as untransfected C3H10T1/2 cells (**Figs 3**, **4** and **5**).

In summary, we have stably-transfected C3H10T1/2 cells with *mRuby2* fluorescence reporter gene. These cells exhibit little-to-no change with respect to cell proliferation and tri-lineage mesenchymal cell differentiation when compared to untransfected C3H10T1/2 cells. Thus, these may have potential uses as a surrogate MSC in preclinical studies.

## Supporting Information

S1 Data Raw data used in this study. Within the Data.zip file, there are 6 folders, whose name corresponds to each individual figure of our manuscript. Raw data including microscope and gel images as well as calculations are contained within each of these folders. Fig01 folder contains 2 flow cytometry plots for Figure 1A and 1C in pdf format as well as 12 phase-contrast and fluorescence microscope images for Figures 1B and 1D. Fig02 folder contains an excel sheet for cell counting and cell doubling data for Figure 2A and 2B. Fig03 folder contains 4 oil red o-stained microscope images for Figure 3A, and 1 agarose gel image (PCR data) as well as an excel sheet for calculating adipogenic gene expression data in Figure 3B. Fig04 folder contains 4 phase-contrast microscope images for Figure 4A, 6 alcian blue-stained microscope images for Figure 4B, and 2 agarose gel images (PCR data) as well as an excel sheet for calculating chondrogenic gene expression data in Figure 4C. Fig05 folder contains 4 ALP-stained microscope images for Figure 5A, an excel sheet for quantifying ALP activity in Figure 5B, 4 alizain red-stained microscope images for Figure 5C, an excel sheet for quantifying alizarin red staining in Figure 5D, and 3 agarose gel images (PCR data) as well as an excel sheet for calculating osteogenic gene expression data in Figure 5E. Fig06 folder contains 8 phase-contrast and fluorescence microscope images for Figures 6A and 6B and 2 ALP-stained images for Figure 6C.
(ZIP)
