# Supplementary figures and images for: Development of mRuby2-Transfected C3H10T1/2 Fibroblasts for Musculoskeletal Tissue Engineering
Source: PLoS One. 2015 Sep 25;10(9):e0139054. doi: 10.1371/journal.pone.0139054 (PMC4583363; doi:10.1371/journal.pone.0139054)

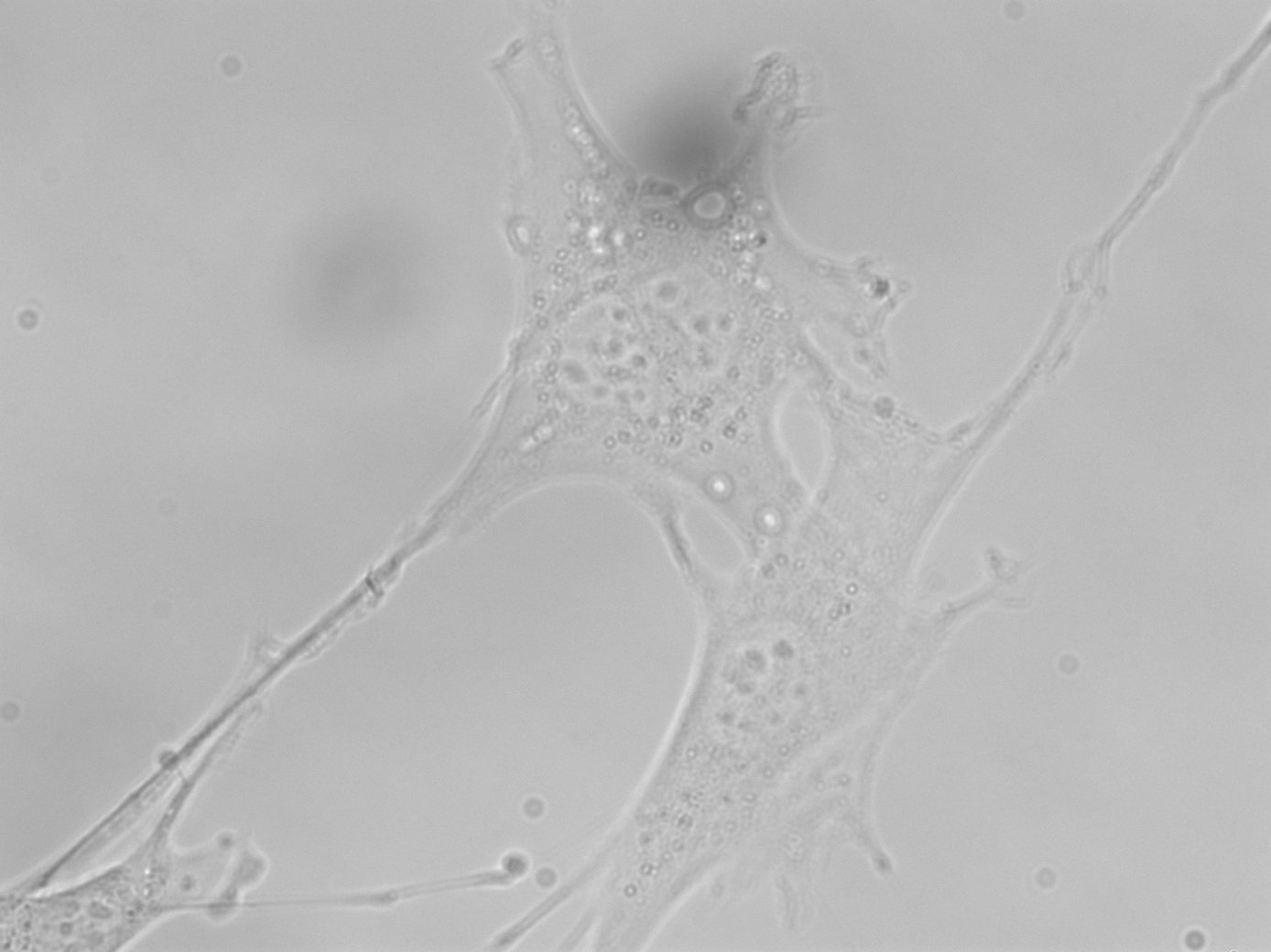

Supplement: S1 Data — Within the Data.zip file, there are 6 folders, whose name corresponds to each individual figure of our manuscript. Raw data including microscope and gel images as well as calculations are contained within each of these folders. Fig01 folder contains 2 flow cytometry plots for Figure 1A and 1C in pdf format as well as 12 phase-contrast and fluorescence microscope images for Figures 1B and 1D. Fig02 folder contains an excel sheet for cell counting and cell doubling data for Figure 2A and 2B. Fig03 folder contains 4 oil red o-stained microscope images for Figure 3A, and 1 agarose gel image (PCR data) as well as an excel sheet for calculating adipogenic gene expression data in Figure 3B. Fig04 folder contains 4 phase-contrast microscope images for Figure 4A, 6 alcian blue-stained microscope images for Figure 4B, and 2 agarose gel images (PCR data) as well as an excel sheet for calculating chondrogenic gene expression data in Figure 4C. Fig05 folder contains 4 ALP-stained microscope images for Figure 5A, an excel sheet for quantifying ALP activity in Figure 5B, 4 alizain red-stained microscope images for Figure 5C, an excel sheet for quantifying alizarin red staining in Figure 5D, and 3 agarose gel images (PCR data) as well as an excel sheet for calculating osteogenic gene expression data in Figure 5E. Fig06 folder contains 8 phase-contrast and fluorescence microscope images for Figures 6A and 6B and 2 ALP-stained images for Figure 6C. (ZIP) [file pone.0139054.s001.zip › Data/Fig01 Flow Cytometry Data/Fig1B 4 40X-C3H10THalfpVitro2MCS-P16pVitro2mRuby2-P14-10s-02-pVitro2-mRuby2-01_c1.TIF]

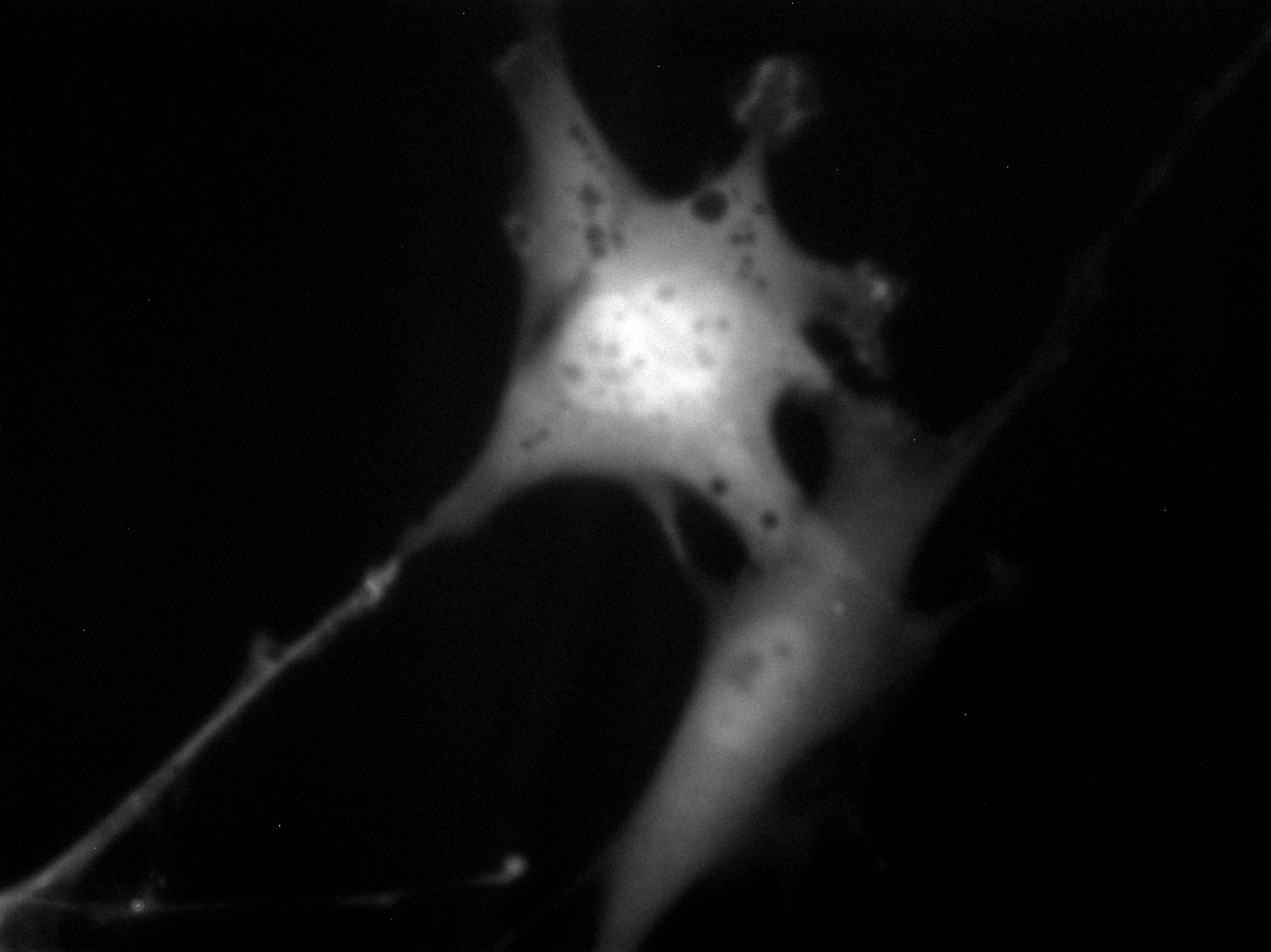

Supplement: S1 Data — Within the Data.zip file, there are 6 folders, whose name corresponds to each individual figure of our manuscript. Raw data including microscope and gel images as well as calculations are contained within each of these folders. Fig01 folder contains 2 flow cytometry plots for Figure 1A and 1C in pdf format as well as 12 phase-contrast and fluorescence microscope images for Figures 1B and 1D. Fig02 folder contains an excel sheet for cell counting and cell doubling data for Figure 2A and 2B. Fig03 folder contains 4 oil red o-stained microscope images for Figure 3A, and 1 agarose gel image (PCR data) as well as an excel sheet for calculating adipogenic gene expression data in Figure 3B. Fig04 folder contains 4 phase-contrast microscope images for Figure 4A, 6 alcian blue-stained microscope images for Figure 4B, and 2 agarose gel images (PCR data) as well as an excel sheet for calculating chondrogenic gene expression data in Figure 4C. Fig05 folder contains 4 ALP-stained microscope images for Figure 5A, an excel sheet for quantifying ALP activity in Figure 5B, 4 alizain red-stained microscope images for Figure 5C, an excel sheet for quantifying alizarin red staining in Figure 5D, and 3 agarose gel images (PCR data) as well as an excel sheet for calculating osteogenic gene expression data in Figure 5E. Fig06 folder contains 8 phase-contrast and fluorescence microscope images for Figures 6A and 6B and 2 ALP-stained images for Figure 6C. (ZIP) [file pone.0139054.s001.zip › Data/Fig01 Flow Cytometry Data/Fig1B 5 40X-C3H10THalfpVitro2MCS-P16pVitro2mRuby2-P14-10s-02-pVitro2-mRuby2-01_c2.TIF]

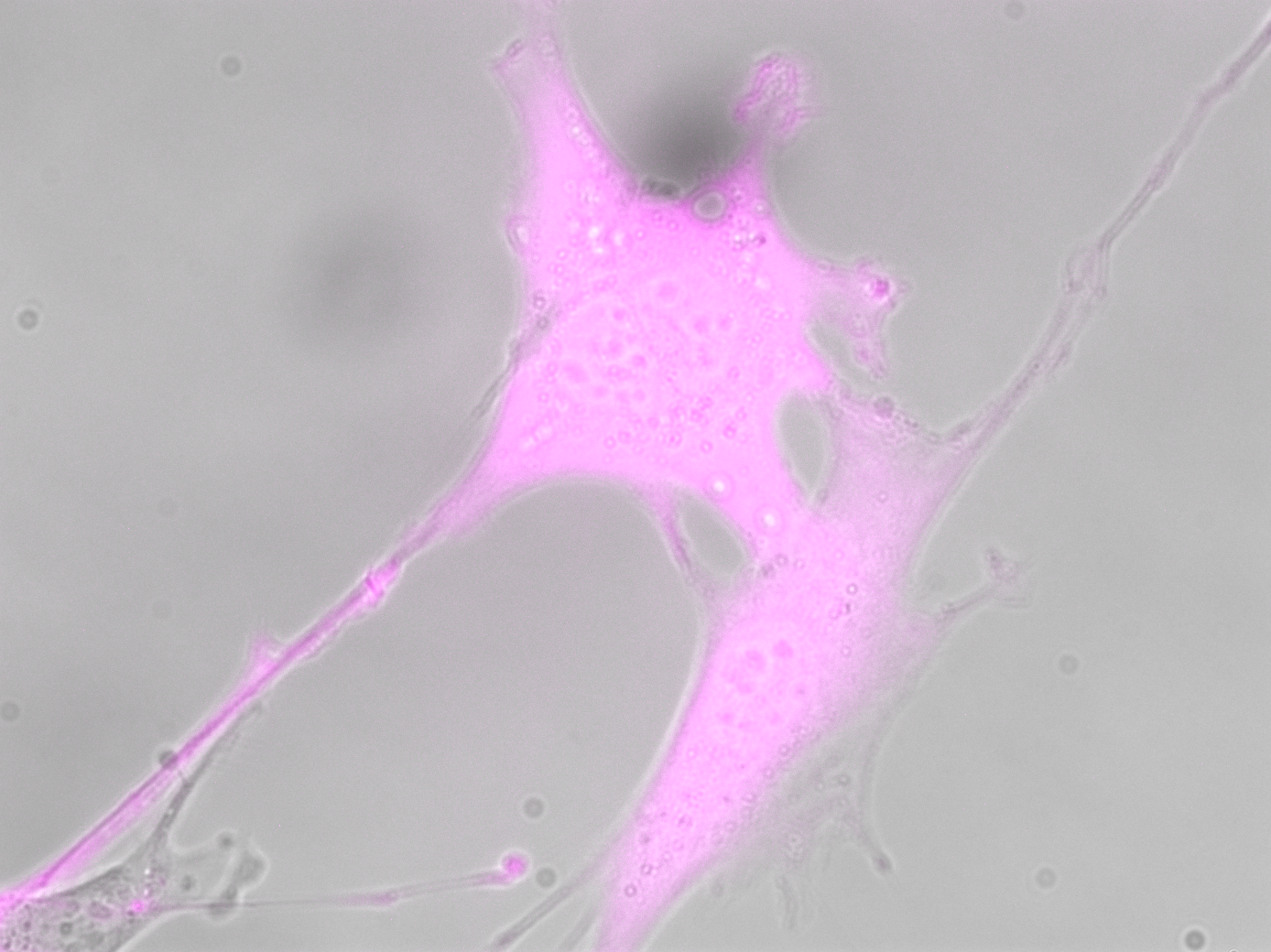

Supplement: S1 Data — Within the Data.zip file, there are 6 folders, whose name corresponds to each individual figure of our manuscript. Raw data including microscope and gel images as well as calculations are contained within each of these folders. Fig01 folder contains 2 flow cytometry plots for Figure 1A and 1C in pdf format as well as 12 phase-contrast and fluorescence microscope images for Figures 1B and 1D. Fig02 folder contains an excel sheet for cell counting and cell doubling data for Figure 2A and 2B. Fig03 folder contains 4 oil red o-stained microscope images for Figure 3A, and 1 agarose gel image (PCR data) as well as an excel sheet for calculating adipogenic gene expression data in Figure 3B. Fig04 folder contains 4 phase-contrast microscope images for Figure 4A, 6 alcian blue-stained microscope images for Figure 4B, and 2 agarose gel images (PCR data) as well as an excel sheet for calculating chondrogenic gene expression data in Figure 4C. Fig05 folder contains 4 ALP-stained microscope images for Figure 5A, an excel sheet for quantifying ALP activity in Figure 5B, 4 alizain red-stained microscope images for Figure 5C, an excel sheet for quantifying alizarin red staining in Figure 5D, and 3 agarose gel images (PCR data) as well as an excel sheet for calculating osteogenic gene expression data in Figure 5E. Fig06 folder contains 8 phase-contrast and fluorescence microscope images for Figures 6A and 6B and 2 ALP-stained images for Figure 6C. (ZIP) [file pone.0139054.s001.zip › Data/Fig01 Flow Cytometry Data/Fig1B 6 40X-C3H10THalfpVitro2MCS-P16pVitro2mRuby2-P14-10s-02-pVitro2-mRuby2-01_(c1+c2).TIF]

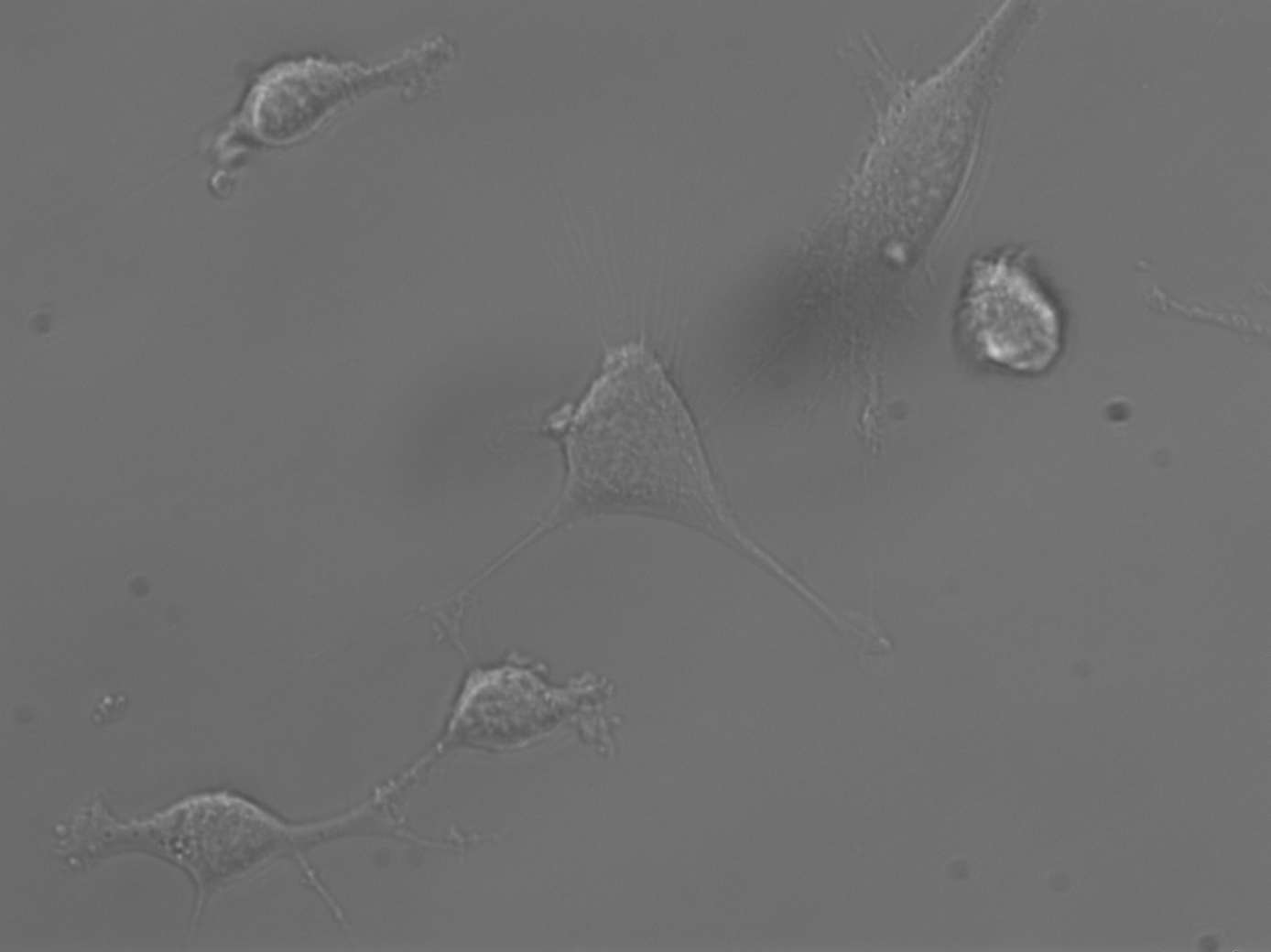

Supplement: S1 Data — Within the Data.zip file, there are 6 folders, whose name corresponds to each individual figure of our manuscript. Raw data including microscope and gel images as well as calculations are contained within each of these folders. Fig01 folder contains 2 flow cytometry plots for Figure 1A and 1C in pdf format as well as 12 phase-contrast and fluorescence microscope images for Figures 1B and 1D. Fig02 folder contains an excel sheet for cell counting and cell doubling data for Figure 2A and 2B. Fig03 folder contains 4 oil red o-stained microscope images for Figure 3A, and 1 agarose gel image (PCR data) as well as an excel sheet for calculating adipogenic gene expression data in Figure 3B. Fig04 folder contains 4 phase-contrast microscope images for Figure 4A, 6 alcian blue-stained microscope images for Figure 4B, and 2 agarose gel images (PCR data) as well as an excel sheet for calculating chondrogenic gene expression data in Figure 4C. Fig05 folder contains 4 ALP-stained microscope images for Figure 5A, an excel sheet for quantifying ALP activity in Figure 5B, 4 alizain red-stained microscope images for Figure 5C, an excel sheet for quantifying alizarin red staining in Figure 5D, and 3 agarose gel images (PCR data) as well as an excel sheet for calculating osteogenic gene expression data in Figure 5E. Fig06 folder contains 8 phase-contrast and fluorescence microscope images for Figures 6A and 6B and 2 ALP-stained images for Figure 6C. (ZIP) [file pone.0139054.s001.zip › Data/Fig01 Flow Cytometry Data/Fig1C 4 40X-mRuby2P27-05_c1.tif]

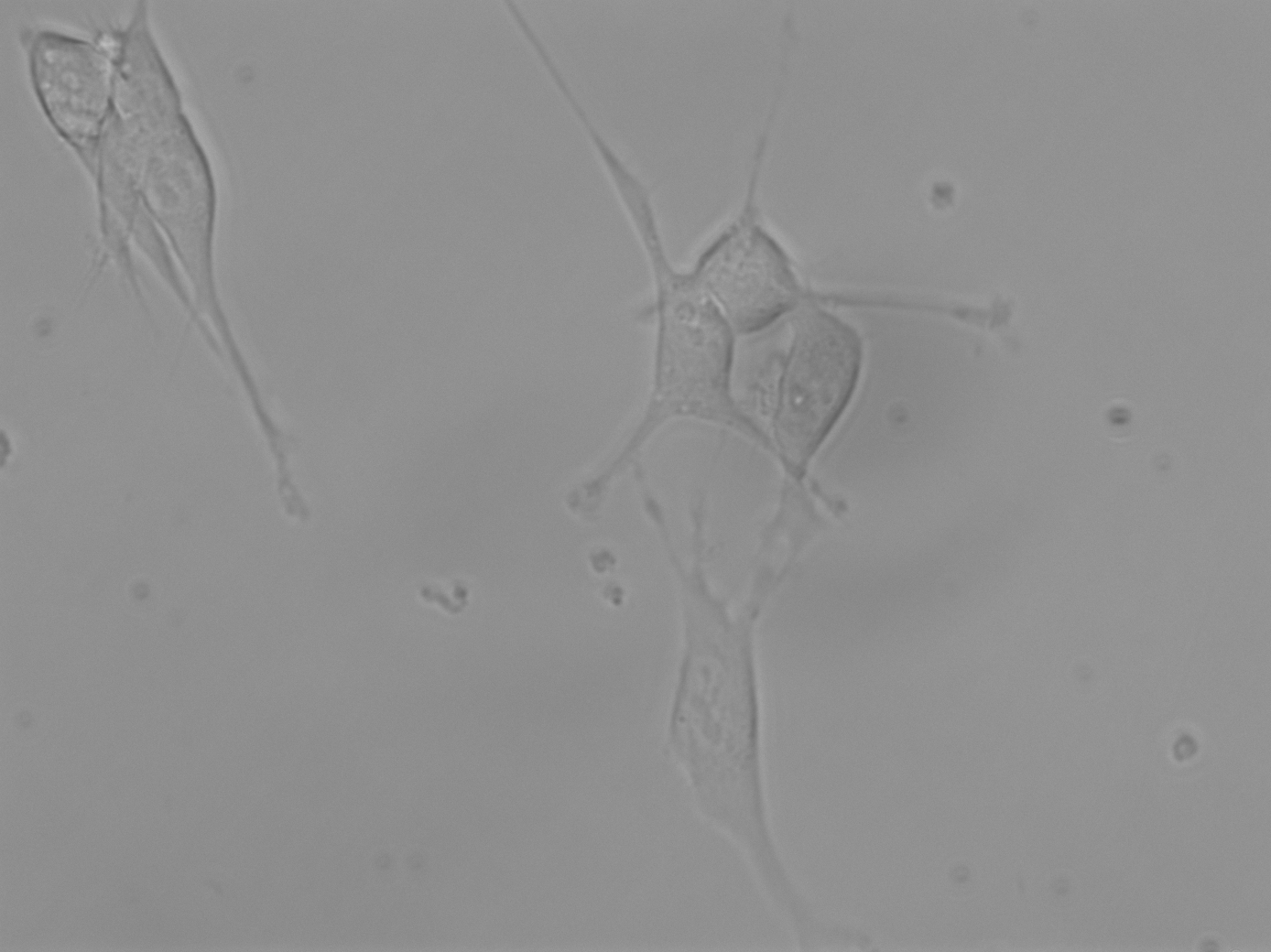

Supplement: S1 Data — Within the Data.zip file, there are 6 folders, whose name corresponds to each individual figure of our manuscript. Raw data including microscope and gel images as well as calculations are contained within each of these folders. Fig01 folder contains 2 flow cytometry plots for Figure 1A and 1C in pdf format as well as 12 phase-contrast and fluorescence microscope images for Figures 1B and 1D. Fig02 folder contains an excel sheet for cell counting and cell doubling data for Figure 2A and 2B. Fig03 folder contains 4 oil red o-stained microscope images for Figure 3A, and 1 agarose gel image (PCR data) as well as an excel sheet for calculating adipogenic gene expression data in Figure 3B. Fig04 folder contains 4 phase-contrast microscope images for Figure 4A, 6 alcian blue-stained microscope images for Figure 4B, and 2 agarose gel images (PCR data) as well as an excel sheet for calculating chondrogenic gene expression data in Figure 4C. Fig05 folder contains 4 ALP-stained microscope images for Figure 5A, an excel sheet for quantifying ALP activity in Figure 5B, 4 alizain red-stained microscope images for Figure 5C, an excel sheet for quantifying alizarin red staining in Figure 5D, and 3 agarose gel images (PCR data) as well as an excel sheet for calculating osteogenic gene expression data in Figure 5E. Fig06 folder contains 8 phase-contrast and fluorescence microscope images for Figures 6A and 6B and 2 ALP-stained images for Figure 6C. (ZIP) [file pone.0139054.s001.zip › Data/Fig01 Flow Cytometry Data/Fig1D 1 40X-C3HP35-02_c1.tif]

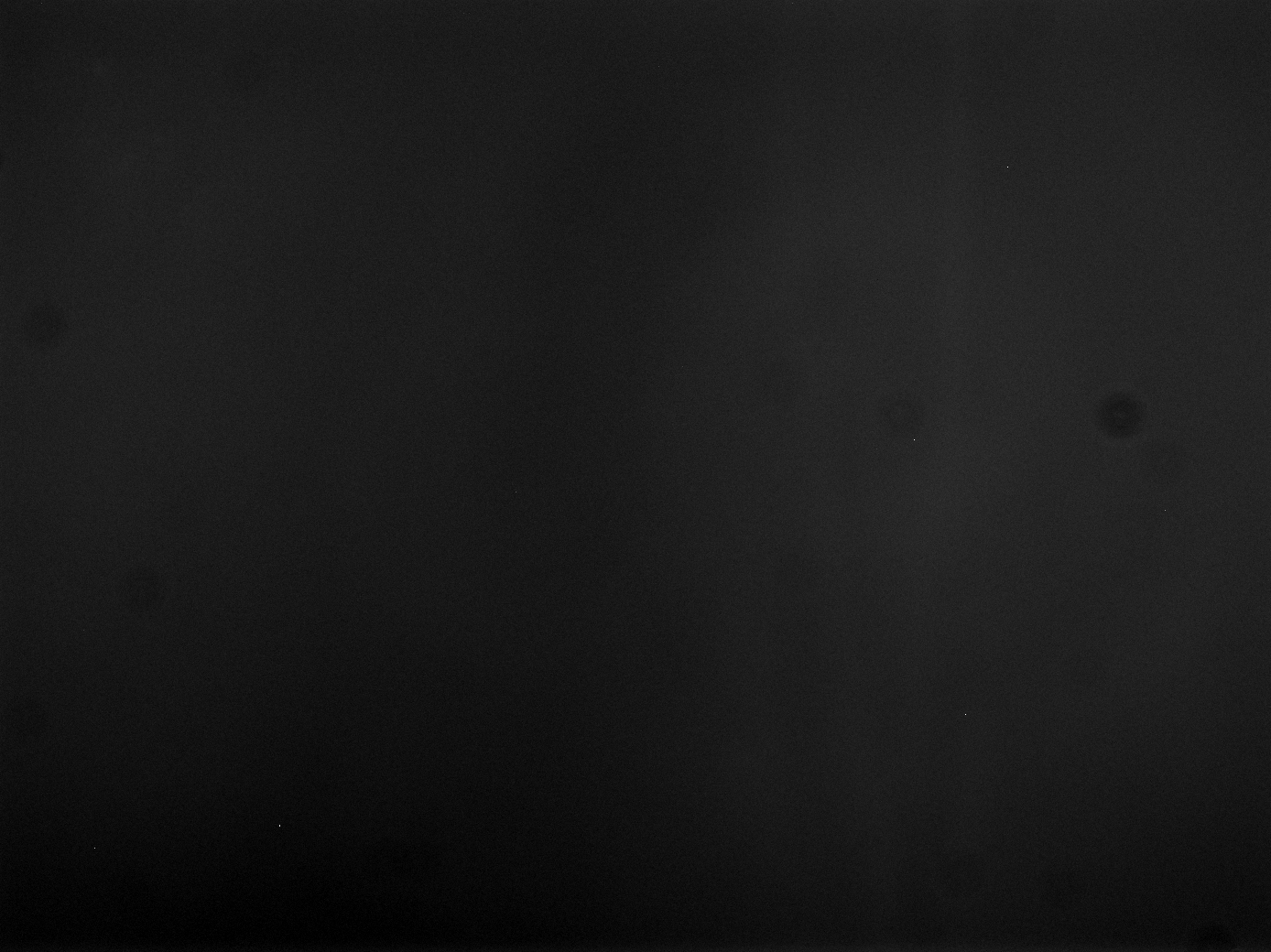

Supplement: S1 Data — Within the Data.zip file, there are 6 folders, whose name corresponds to each individual figure of our manuscript. Raw data including microscope and gel images as well as calculations are contained within each of these folders. Fig01 folder contains 2 flow cytometry plots for Figure 1A and 1C in pdf format as well as 12 phase-contrast and fluorescence microscope images for Figures 1B and 1D. Fig02 folder contains an excel sheet for cell counting and cell doubling data for Figure 2A and 2B. Fig03 folder contains 4 oil red o-stained microscope images for Figure 3A, and 1 agarose gel image (PCR data) as well as an excel sheet for calculating adipogenic gene expression data in Figure 3B. Fig04 folder contains 4 phase-contrast microscope images for Figure 4A, 6 alcian blue-stained microscope images for Figure 4B, and 2 agarose gel images (PCR data) as well as an excel sheet for calculating chondrogenic gene expression data in Figure 4C. Fig05 folder contains 4 ALP-stained microscope images for Figure 5A, an excel sheet for quantifying ALP activity in Figure 5B, 4 alizain red-stained microscope images for Figure 5C, an excel sheet for quantifying alizarin red staining in Figure 5D, and 3 agarose gel images (PCR data) as well as an excel sheet for calculating osteogenic gene expression data in Figure 5E. Fig06 folder contains 8 phase-contrast and fluorescence microscope images for Figures 6A and 6B and 2 ALP-stained images for Figure 6C. (ZIP) [file pone.0139054.s001.zip › Data/Fig01 Flow Cytometry Data/Fig1D 2 40X-C3HP35-02_c2.tif]

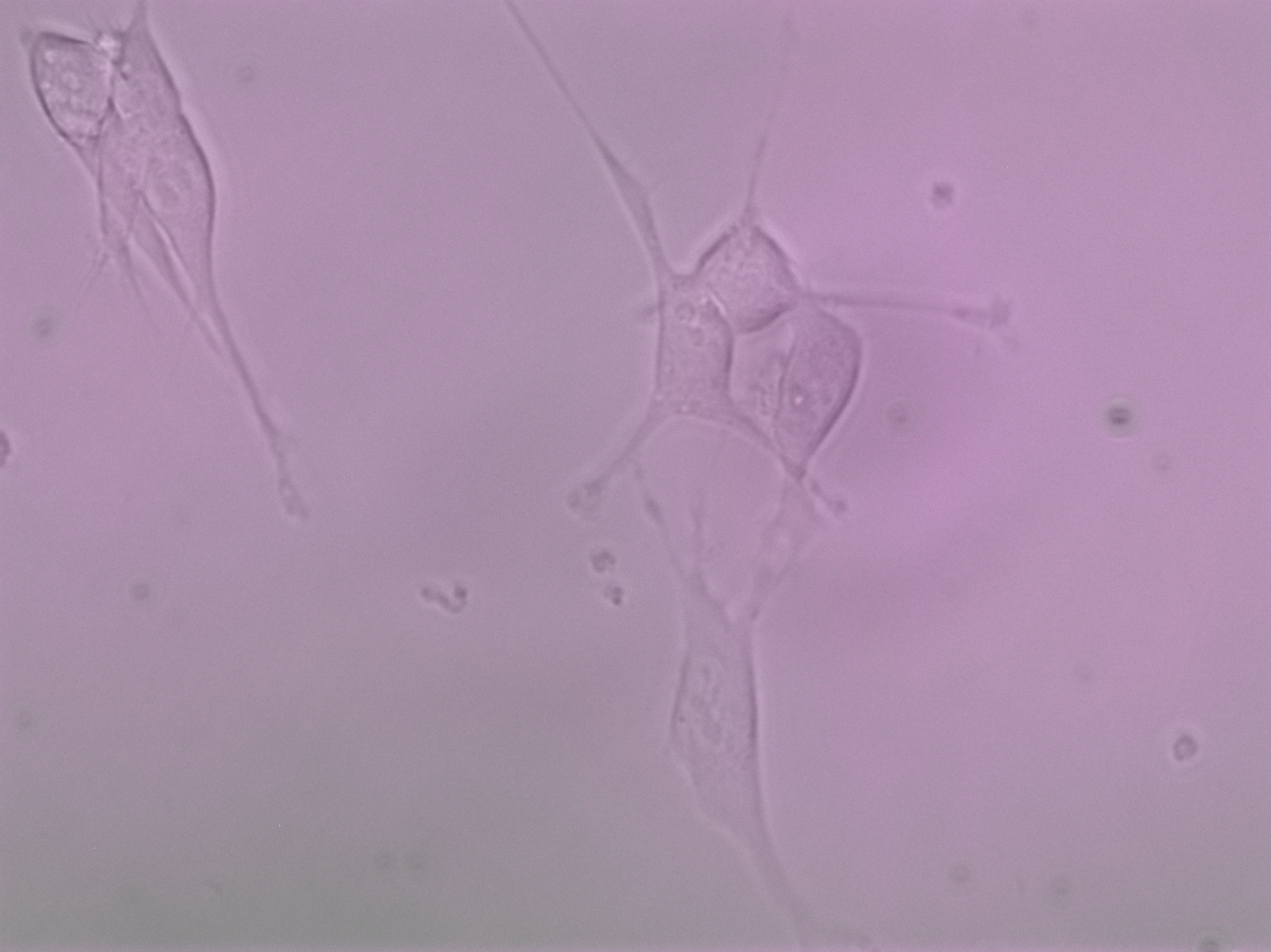

Supplement: S1 Data — Within the Data.zip file, there are 6 folders, whose name corresponds to each individual figure of our manuscript. Raw data including microscope and gel images as well as calculations are contained within each of these folders. Fig01 folder contains 2 flow cytometry plots for Figure 1A and 1C in pdf format as well as 12 phase-contrast and fluorescence microscope images for Figures 1B and 1D. Fig02 folder contains an excel sheet for cell counting and cell doubling data for Figure 2A and 2B. Fig03 folder contains 4 oil red o-stained microscope images for Figure 3A, and 1 agarose gel image (PCR data) as well as an excel sheet for calculating adipogenic gene expression data in Figure 3B. Fig04 folder contains 4 phase-contrast microscope images for Figure 4A, 6 alcian blue-stained microscope images for Figure 4B, and 2 agarose gel images (PCR data) as well as an excel sheet for calculating chondrogenic gene expression data in Figure 4C. Fig05 folder contains 4 ALP-stained microscope images for Figure 5A, an excel sheet for quantifying ALP activity in Figure 5B, 4 alizain red-stained microscope images for Figure 5C, an excel sheet for quantifying alizarin red staining in Figure 5D, and 3 agarose gel images (PCR data) as well as an excel sheet for calculating osteogenic gene expression data in Figure 5E. Fig06 folder contains 8 phase-contrast and fluorescence microscope images for Figures 6A and 6B and 2 ALP-stained images for Figure 6C. (ZIP) [file pone.0139054.s001.zip › Data/Fig01 Flow Cytometry Data/Fig1D 3 40X-C3HP35-02_c1+2.tif]

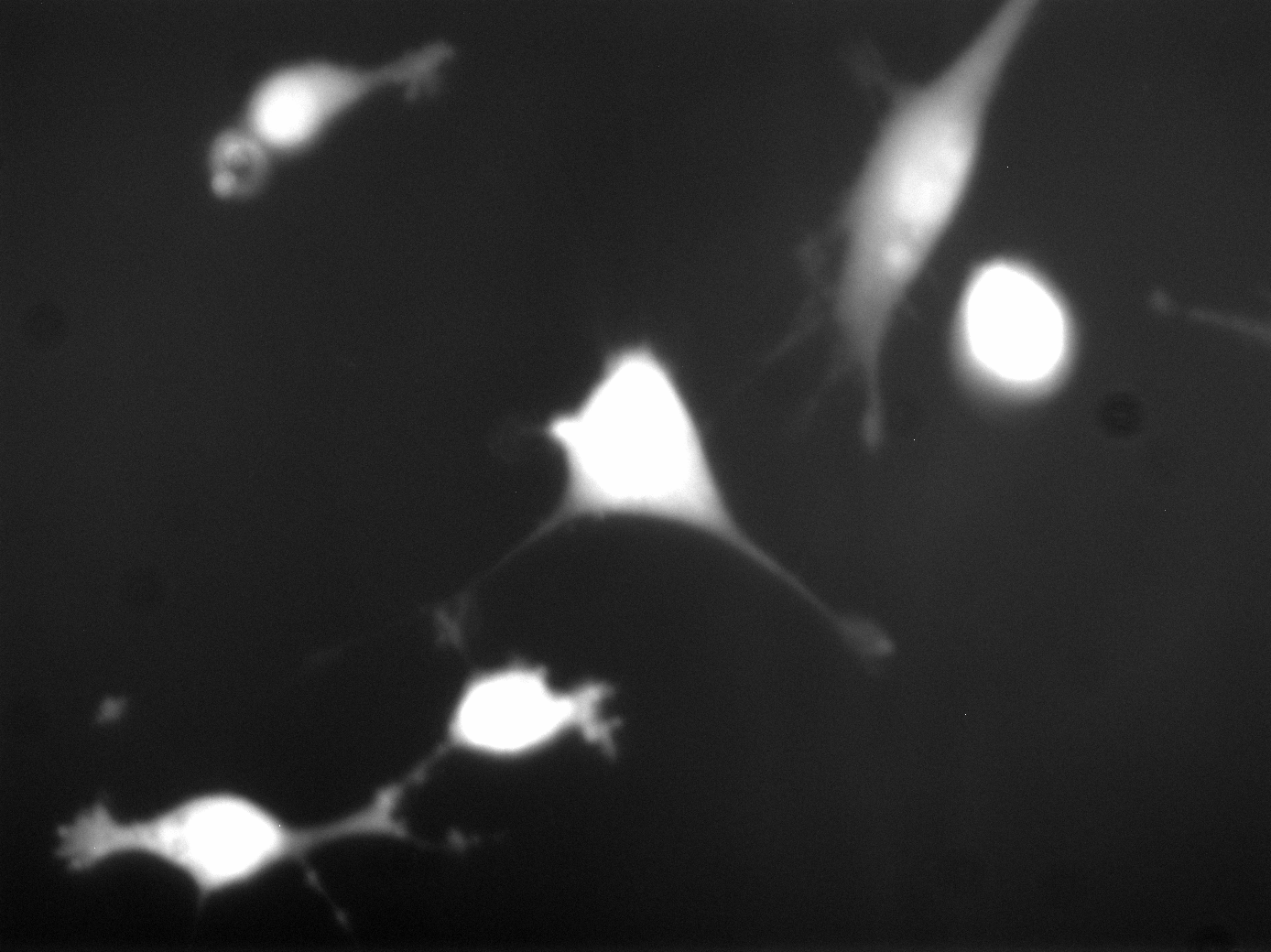

Supplement: S1 Data — Within the Data.zip file, there are 6 folders, whose name corresponds to each individual figure of our manuscript. Raw data including microscope and gel images as well as calculations are contained within each of these folders. Fig01 folder contains 2 flow cytometry plots for Figure 1A and 1C in pdf format as well as 12 phase-contrast and fluorescence microscope images for Figures 1B and 1D. Fig02 folder contains an excel sheet for cell counting and cell doubling data for Figure 2A and 2B. Fig03 folder contains 4 oil red o-stained microscope images for Figure 3A, and 1 agarose gel image (PCR data) as well as an excel sheet for calculating adipogenic gene expression data in Figure 3B. Fig04 folder contains 4 phase-contrast microscope images for Figure 4A, 6 alcian blue-stained microscope images for Figure 4B, and 2 agarose gel images (PCR data) as well as an excel sheet for calculating chondrogenic gene expression data in Figure 4C. Fig05 folder contains 4 ALP-stained microscope images for Figure 5A, an excel sheet for quantifying ALP activity in Figure 5B, 4 alizain red-stained microscope images for Figure 5C, an excel sheet for quantifying alizarin red staining in Figure 5D, and 3 agarose gel images (PCR data) as well as an excel sheet for calculating osteogenic gene expression data in Figure 5E. Fig06 folder contains 8 phase-contrast and fluorescence microscope images for Figures 6A and 6B and 2 ALP-stained images for Figure 6C. (ZIP) [file pone.0139054.s001.zip › Data/Fig01 Flow Cytometry Data/Fig1D 5 40X-mRuby2P27-05_c2.tif]

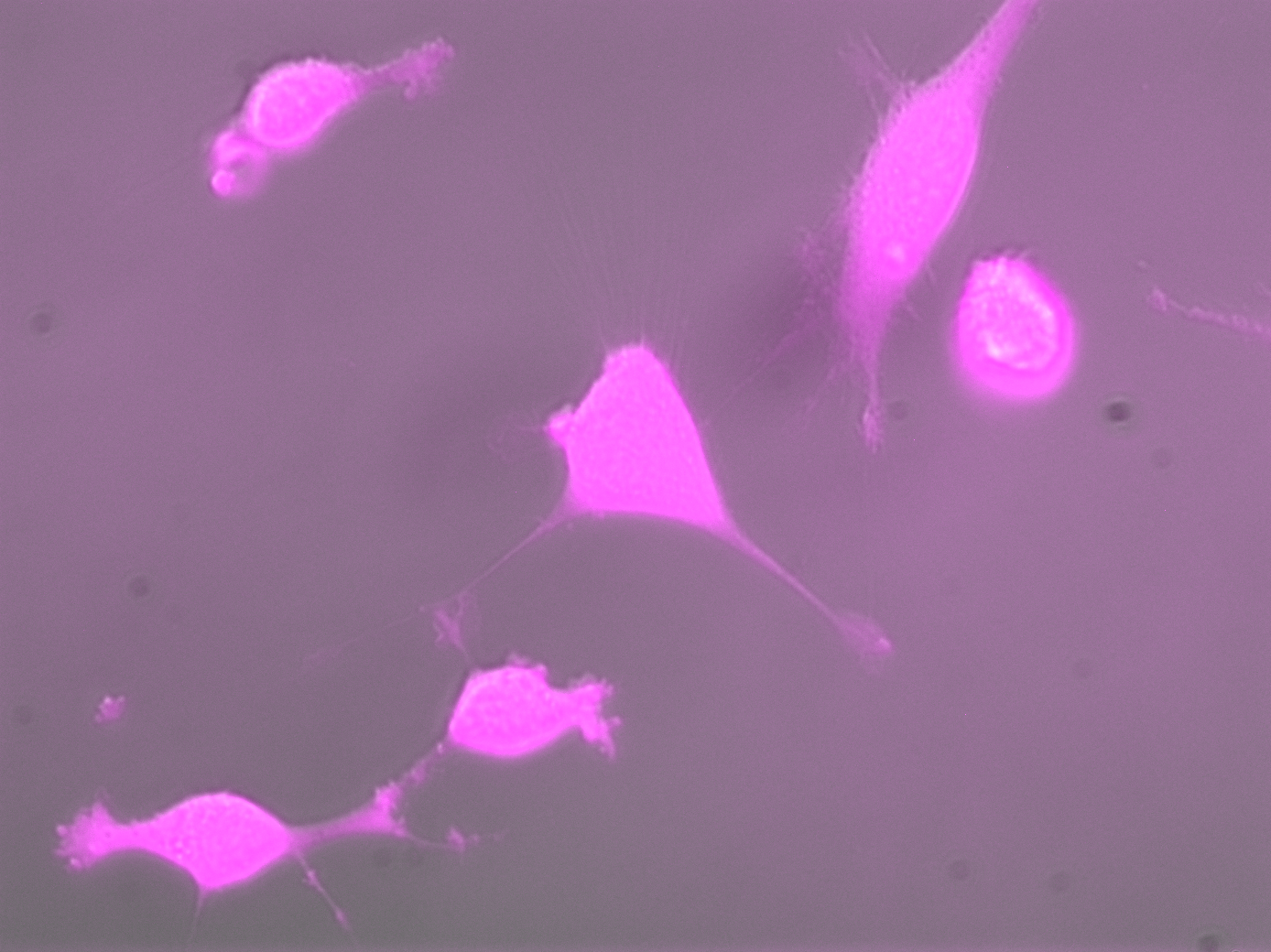

Supplement: S1 Data — Within the Data.zip file, there are 6 folders, whose name corresponds to each individual figure of our manuscript. Raw data including microscope and gel images as well as calculations are contained within each of these folders. Fig01 folder contains 2 flow cytometry plots for Figure 1A and 1C in pdf format as well as 12 phase-contrast and fluorescence microscope images for Figures 1B and 1D. Fig02 folder contains an excel sheet for cell counting and cell doubling data for Figure 2A and 2B. Fig03 folder contains 4 oil red o-stained microscope images for Figure 3A, and 1 agarose gel image (PCR data) as well as an excel sheet for calculating adipogenic gene expression data in Figure 3B. Fig04 folder contains 4 phase-contrast microscope images for Figure 4A, 6 alcian blue-stained microscope images for Figure 4B, and 2 agarose gel images (PCR data) as well as an excel sheet for calculating chondrogenic gene expression data in Figure 4C. Fig05 folder contains 4 ALP-stained microscope images for Figure 5A, an excel sheet for quantifying ALP activity in Figure 5B, 4 alizain red-stained microscope images for Figure 5C, an excel sheet for quantifying alizarin red staining in Figure 5D, and 3 agarose gel images (PCR data) as well as an excel sheet for calculating osteogenic gene expression data in Figure 5E. Fig06 folder contains 8 phase-contrast and fluorescence microscope images for Figures 6A and 6B and 2 ALP-stained images for Figure 6C. (ZIP) [file pone.0139054.s001.zip › Data/Fig01 Flow Cytometry Data/Fig1D 6 40X-mRuby2P27-05_c1+2.tif]

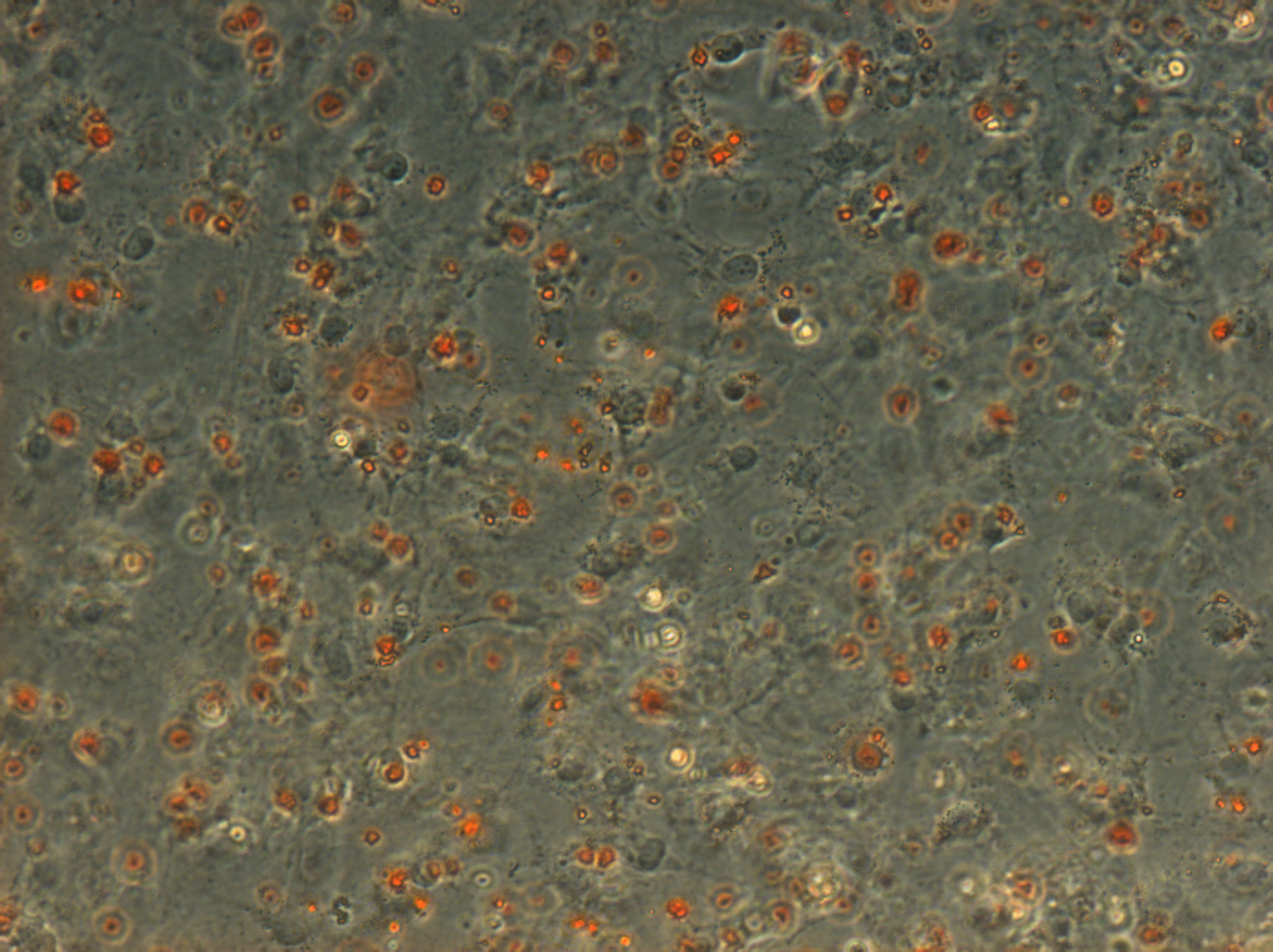

Supplement: S1 Data — Within the Data.zip file, there are 6 folders, whose name corresponds to each individual figure of our manuscript. Raw data including microscope and gel images as well as calculations are contained within each of these folders. Fig01 folder contains 2 flow cytometry plots for Figure 1A and 1C in pdf format as well as 12 phase-contrast and fluorescence microscope images for Figures 1B and 1D. Fig02 folder contains an excel sheet for cell counting and cell doubling data for Figure 2A and 2B. Fig03 folder contains 4 oil red o-stained microscope images for Figure 3A, and 1 agarose gel image (PCR data) as well as an excel sheet for calculating adipogenic gene expression data in Figure 3B. Fig04 folder contains 4 phase-contrast microscope images for Figure 4A, 6 alcian blue-stained microscope images for Figure 4B, and 2 agarose gel images (PCR data) as well as an excel sheet for calculating chondrogenic gene expression data in Figure 4C. Fig05 folder contains 4 ALP-stained microscope images for Figure 5A, an excel sheet for quantifying ALP activity in Figure 5B, 4 alizain red-stained microscope images for Figure 5C, an excel sheet for quantifying alizarin red staining in Figure 5D, and 3 agarose gel images (PCR data) as well as an excel sheet for calculating osteogenic gene expression data in Figure 5E. Fig06 folder contains 8 phase-contrast and fluorescence microscope images for Figures 6A and 6B and 2 ALP-stained images for Figure 6C. (ZIP) [file pone.0139054.s001.zip › Data/Fig03 Adipogenic Data/ORO Set03 01-15DayAdipogenesis-2p7V-Exposure250ms-ColorOffset0-C3H10THalf-Control01.TIF]

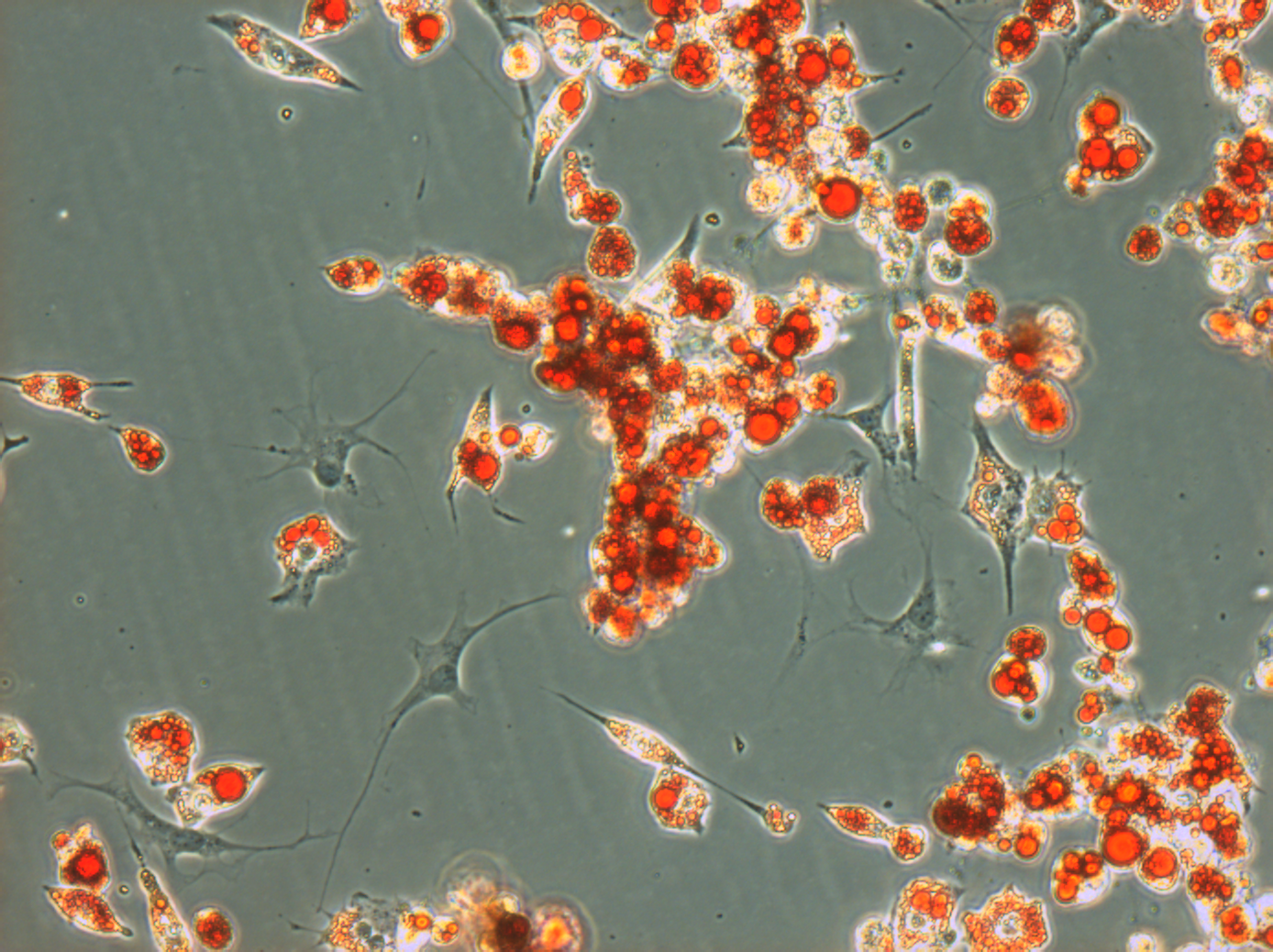

Supplement: S1 Data — Within the Data.zip file, there are 6 folders, whose name corresponds to each individual figure of our manuscript. Raw data including microscope and gel images as well as calculations are contained within each of these folders. Fig01 folder contains 2 flow cytometry plots for Figure 1A and 1C in pdf format as well as 12 phase-contrast and fluorescence microscope images for Figures 1B and 1D. Fig02 folder contains an excel sheet for cell counting and cell doubling data for Figure 2A and 2B. Fig03 folder contains 4 oil red o-stained microscope images for Figure 3A, and 1 agarose gel image (PCR data) as well as an excel sheet for calculating adipogenic gene expression data in Figure 3B. Fig04 folder contains 4 phase-contrast microscope images for Figure 4A, 6 alcian blue-stained microscope images for Figure 4B, and 2 agarose gel images (PCR data) as well as an excel sheet for calculating chondrogenic gene expression data in Figure 4C. Fig05 folder contains 4 ALP-stained microscope images for Figure 5A, an excel sheet for quantifying ALP activity in Figure 5B, 4 alizain red-stained microscope images for Figure 5C, an excel sheet for quantifying alizarin red staining in Figure 5D, and 3 agarose gel images (PCR data) as well as an excel sheet for calculating osteogenic gene expression data in Figure 5E. Fig06 folder contains 8 phase-contrast and fluorescence microscope images for Figures 6A and 6B and 2 ALP-stained images for Figure 6C. (ZIP) [file pone.0139054.s001.zip › Data/Fig03 Adipogenic Data/ORO Set03 02-15DayAdipogenesis-2p7V-Exposure250ms-ColorOffset0-C3H10THalf-Adipogenesis03.TIF]

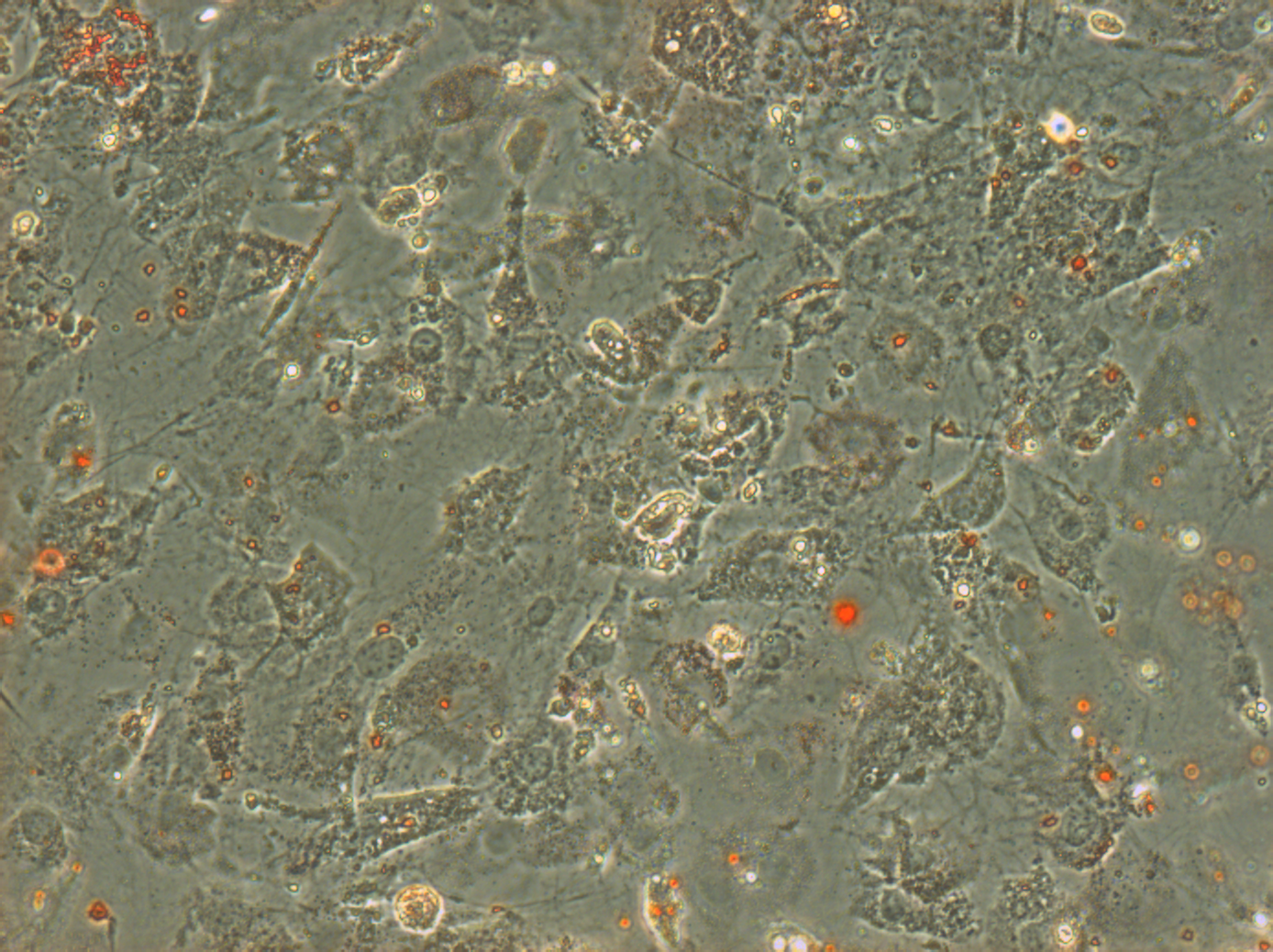

Supplement: S1 Data — Within the Data.zip file, there are 6 folders, whose name corresponds to each individual figure of our manuscript. Raw data including microscope and gel images as well as calculations are contained within each of these folders. Fig01 folder contains 2 flow cytometry plots for Figure 1A and 1C in pdf format as well as 12 phase-contrast and fluorescence microscope images for Figures 1B and 1D. Fig02 folder contains an excel sheet for cell counting and cell doubling data for Figure 2A and 2B. Fig03 folder contains 4 oil red o-stained microscope images for Figure 3A, and 1 agarose gel image (PCR data) as well as an excel sheet for calculating adipogenic gene expression data in Figure 3B. Fig04 folder contains 4 phase-contrast microscope images for Figure 4A, 6 alcian blue-stained microscope images for Figure 4B, and 2 agarose gel images (PCR data) as well as an excel sheet for calculating chondrogenic gene expression data in Figure 4C. Fig05 folder contains 4 ALP-stained microscope images for Figure 5A, an excel sheet for quantifying ALP activity in Figure 5B, 4 alizain red-stained microscope images for Figure 5C, an excel sheet for quantifying alizarin red staining in Figure 5D, and 3 agarose gel images (PCR data) as well as an excel sheet for calculating osteogenic gene expression data in Figure 5E. Fig06 folder contains 8 phase-contrast and fluorescence microscope images for Figures 6A and 6B and 2 ALP-stained images for Figure 6C. (ZIP) [file pone.0139054.s001.zip › Data/Fig03 Adipogenic Data/ORO Set03 03-15DayAdipogenesis-2p7V-Exposure250ms-ColorOffset0-C3H10THalfmRuby2-Control01.TIF]

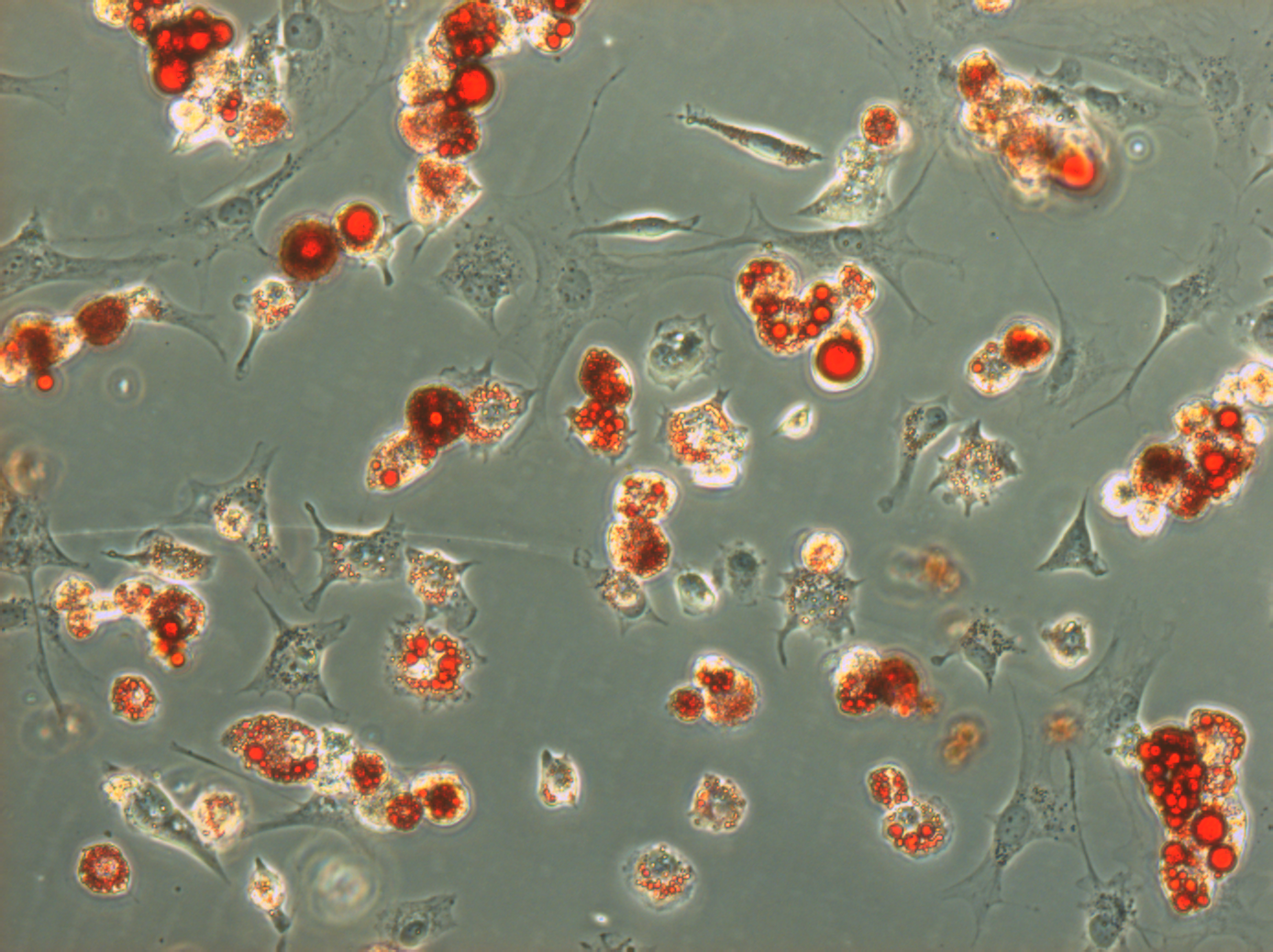

Supplement: S1 Data — Within the Data.zip file, there are 6 folders, whose name corresponds to each individual figure of our manuscript. Raw data including microscope and gel images as well as calculations are contained within each of these folders. Fig01 folder contains 2 flow cytometry plots for Figure 1A and 1C in pdf format as well as 12 phase-contrast and fluorescence microscope images for Figures 1B and 1D. Fig02 folder contains an excel sheet for cell counting and cell doubling data for Figure 2A and 2B. Fig03 folder contains 4 oil red o-stained microscope images for Figure 3A, and 1 agarose gel image (PCR data) as well as an excel sheet for calculating adipogenic gene expression data in Figure 3B. Fig04 folder contains 4 phase-contrast microscope images for Figure 4A, 6 alcian blue-stained microscope images for Figure 4B, and 2 agarose gel images (PCR data) as well as an excel sheet for calculating chondrogenic gene expression data in Figure 4C. Fig05 folder contains 4 ALP-stained microscope images for Figure 5A, an excel sheet for quantifying ALP activity in Figure 5B, 4 alizain red-stained microscope images for Figure 5C, an excel sheet for quantifying alizarin red staining in Figure 5D, and 3 agarose gel images (PCR data) as well as an excel sheet for calculating osteogenic gene expression data in Figure 5E. Fig06 folder contains 8 phase-contrast and fluorescence microscope images for Figures 6A and 6B and 2 ALP-stained images for Figure 6C. (ZIP) [file pone.0139054.s001.zip › Data/Fig03 Adipogenic Data/ORO Set03 04-15DayAdipogenesis-2p7V-Exposure250ms-ColorOffset0-C3H10THalfmRuby2-Adipogenesis03.TIF]

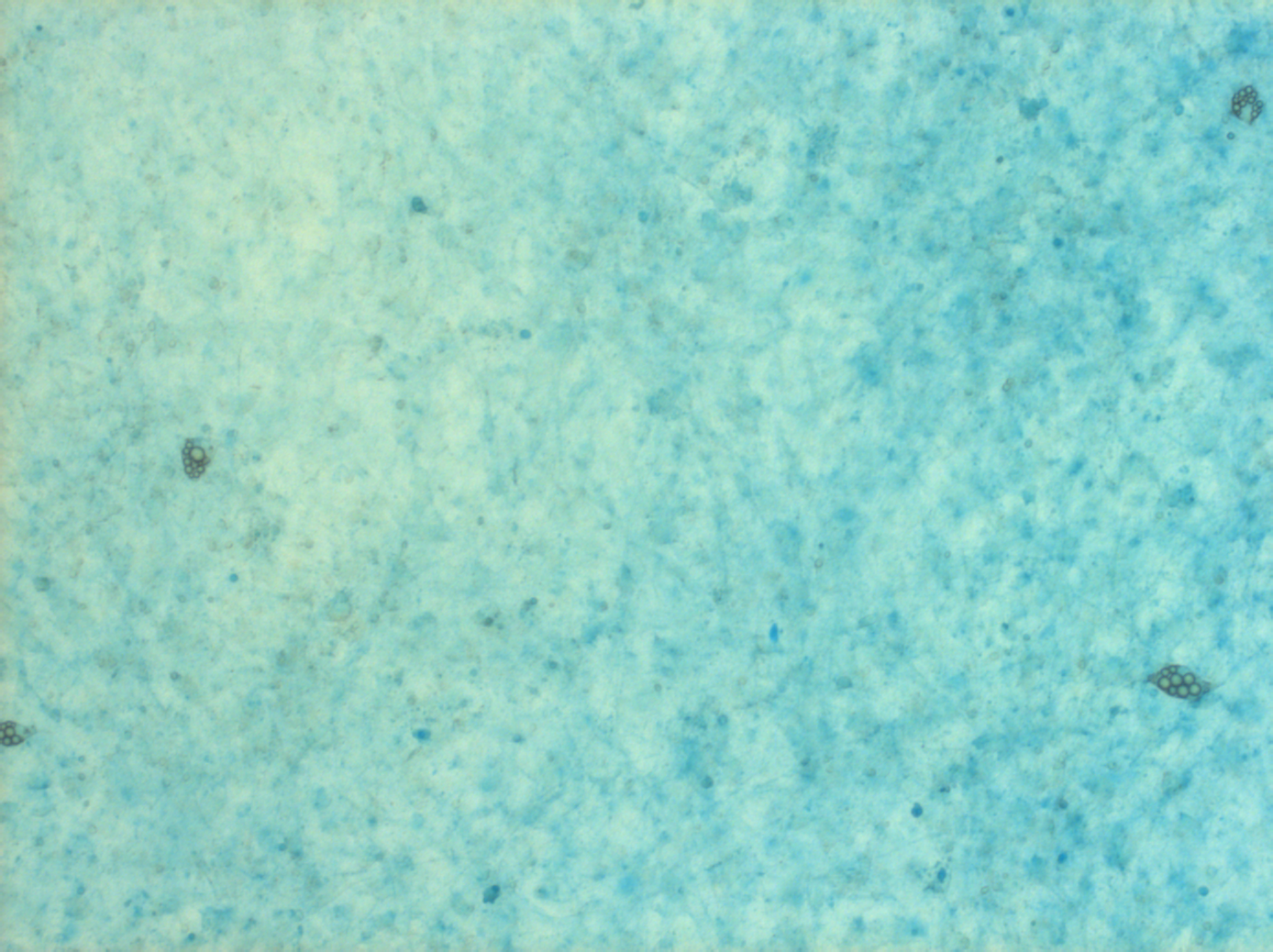

Supplement: S1 Data — Within the Data.zip file, there are 6 folders, whose name corresponds to each individual figure of our manuscript. Raw data including microscope and gel images as well as calculations are contained within each of these folders. Fig01 folder contains 2 flow cytometry plots for Figure 1A and 1C in pdf format as well as 12 phase-contrast and fluorescence microscope images for Figures 1B and 1D. Fig02 folder contains an excel sheet for cell counting and cell doubling data for Figure 2A and 2B. Fig03 folder contains 4 oil red o-stained microscope images for Figure 3A, and 1 agarose gel image (PCR data) as well as an excel sheet for calculating adipogenic gene expression data in Figure 3B. Fig04 folder contains 4 phase-contrast microscope images for Figure 4A, 6 alcian blue-stained microscope images for Figure 4B, and 2 agarose gel images (PCR data) as well as an excel sheet for calculating chondrogenic gene expression data in Figure 4C. Fig05 folder contains 4 ALP-stained microscope images for Figure 5A, an excel sheet for quantifying ALP activity in Figure 5B, 4 alizain red-stained microscope images for Figure 5C, an excel sheet for quantifying alizarin red staining in Figure 5D, and 3 agarose gel images (PCR data) as well as an excel sheet for calculating osteogenic gene expression data in Figure 5E. Fig06 folder contains 8 phase-contrast and fluorescence microscope images for Figures 6A and 6B and 2 ALP-stained images for Figure 6C. (ZIP) [file pone.0139054.s001.zip › Data/Fig04 Chondrogenic Data/AB 01a-Set01-05X-C3H10THalf-Control03.TIF]

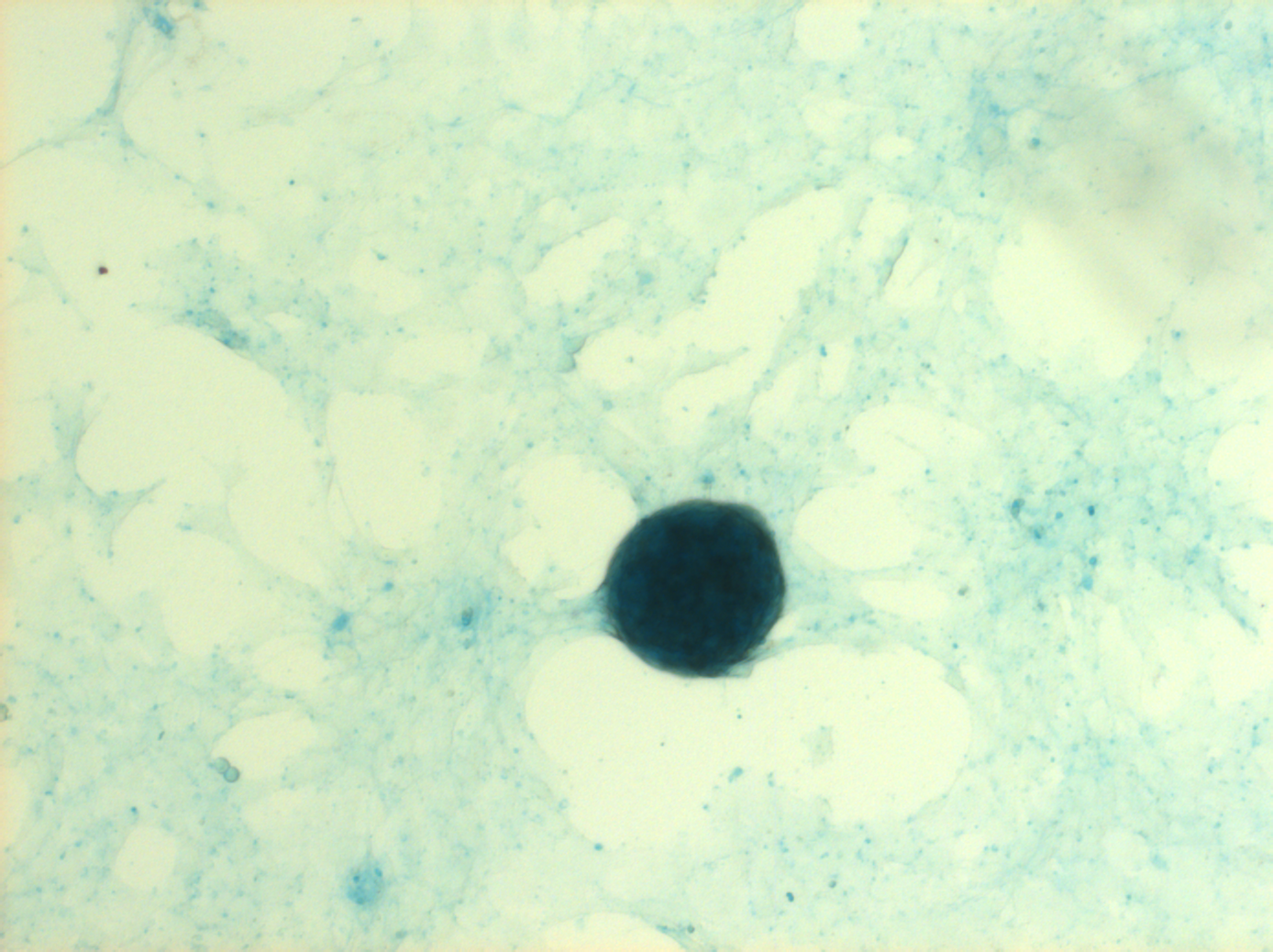

Supplement: S1 Data — Within the Data.zip file, there are 6 folders, whose name corresponds to each individual figure of our manuscript. Raw data including microscope and gel images as well as calculations are contained within each of these folders. Fig01 folder contains 2 flow cytometry plots for Figure 1A and 1C in pdf format as well as 12 phase-contrast and fluorescence microscope images for Figures 1B and 1D. Fig02 folder contains an excel sheet for cell counting and cell doubling data for Figure 2A and 2B. Fig03 folder contains 4 oil red o-stained microscope images for Figure 3A, and 1 agarose gel image (PCR data) as well as an excel sheet for calculating adipogenic gene expression data in Figure 3B. Fig04 folder contains 4 phase-contrast microscope images for Figure 4A, 6 alcian blue-stained microscope images for Figure 4B, and 2 agarose gel images (PCR data) as well as an excel sheet for calculating chondrogenic gene expression data in Figure 4C. Fig05 folder contains 4 ALP-stained microscope images for Figure 5A, an excel sheet for quantifying ALP activity in Figure 5B, 4 alizain red-stained microscope images for Figure 5C, an excel sheet for quantifying alizarin red staining in Figure 5D, and 3 agarose gel images (PCR data) as well as an excel sheet for calculating osteogenic gene expression data in Figure 5E. Fig06 folder contains 8 phase-contrast and fluorescence microscope images for Figures 6A and 6B and 2 ALP-stained images for Figure 6C. (ZIP) [file pone.0139054.s001.zip › Data/Fig04 Chondrogenic Data/AB 01b-Set01-05X-C3H10THalf-Chondro01.TIF]

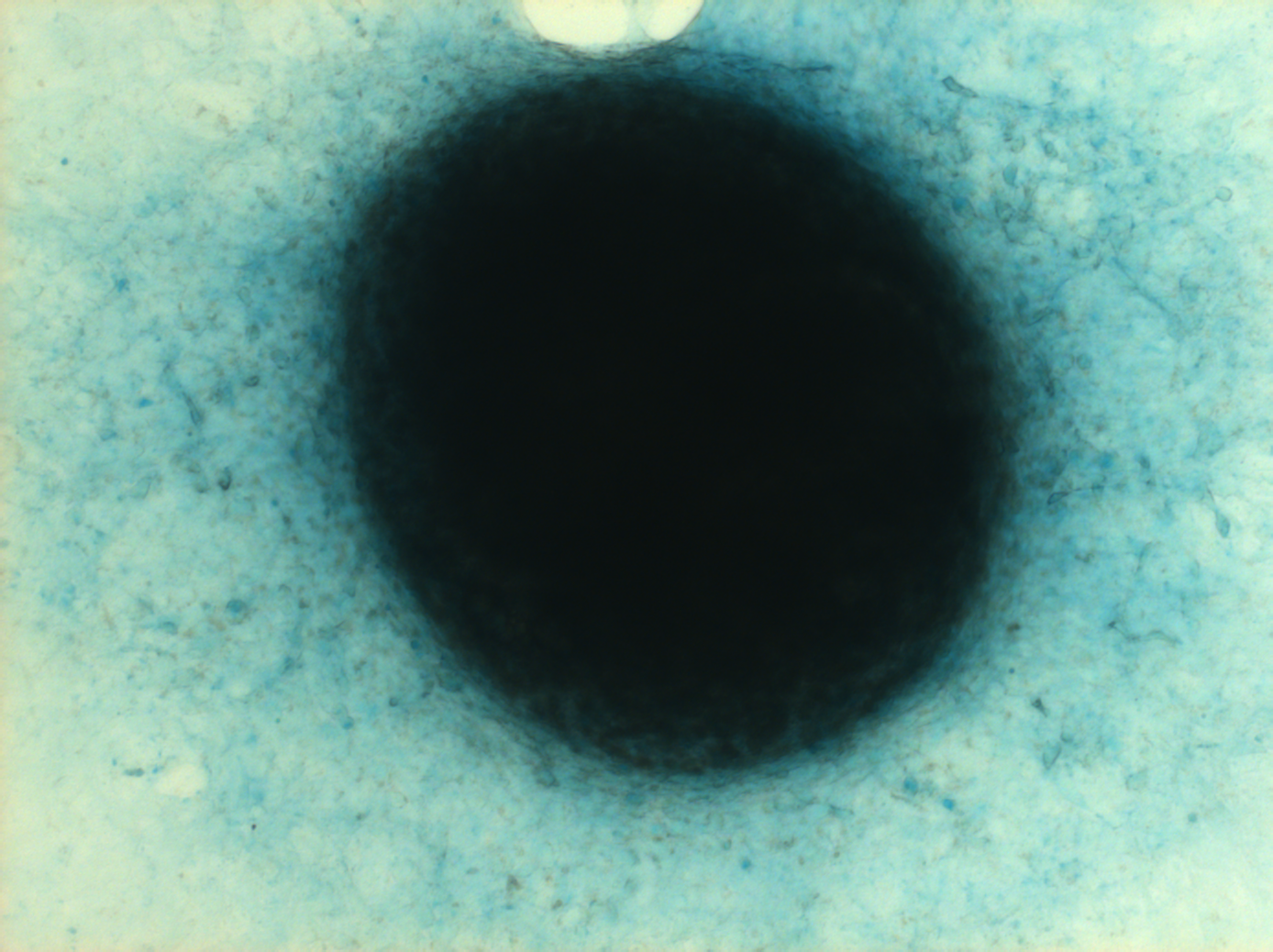

Supplement: S1 Data — Within the Data.zip file, there are 6 folders, whose name corresponds to each individual figure of our manuscript. Raw data including microscope and gel images as well as calculations are contained within each of these folders. Fig01 folder contains 2 flow cytometry plots for Figure 1A and 1C in pdf format as well as 12 phase-contrast and fluorescence microscope images for Figures 1B and 1D. Fig02 folder contains an excel sheet for cell counting and cell doubling data for Figure 2A and 2B. Fig03 folder contains 4 oil red o-stained microscope images for Figure 3A, and 1 agarose gel image (PCR data) as well as an excel sheet for calculating adipogenic gene expression data in Figure 3B. Fig04 folder contains 4 phase-contrast microscope images for Figure 4A, 6 alcian blue-stained microscope images for Figure 4B, and 2 agarose gel images (PCR data) as well as an excel sheet for calculating chondrogenic gene expression data in Figure 4C. Fig05 folder contains 4 ALP-stained microscope images for Figure 5A, an excel sheet for quantifying ALP activity in Figure 5B, 4 alizain red-stained microscope images for Figure 5C, an excel sheet for quantifying alizarin red staining in Figure 5D, and 3 agarose gel images (PCR data) as well as an excel sheet for calculating osteogenic gene expression data in Figure 5E. Fig06 folder contains 8 phase-contrast and fluorescence microscope images for Figures 6A and 6B and 2 ALP-stained images for Figure 6C. (ZIP) [file pone.0139054.s001.zip › Data/Fig04 Chondrogenic Data/AB 01b-Set01-05X-C3H10THalf-Chondro02.TIF]

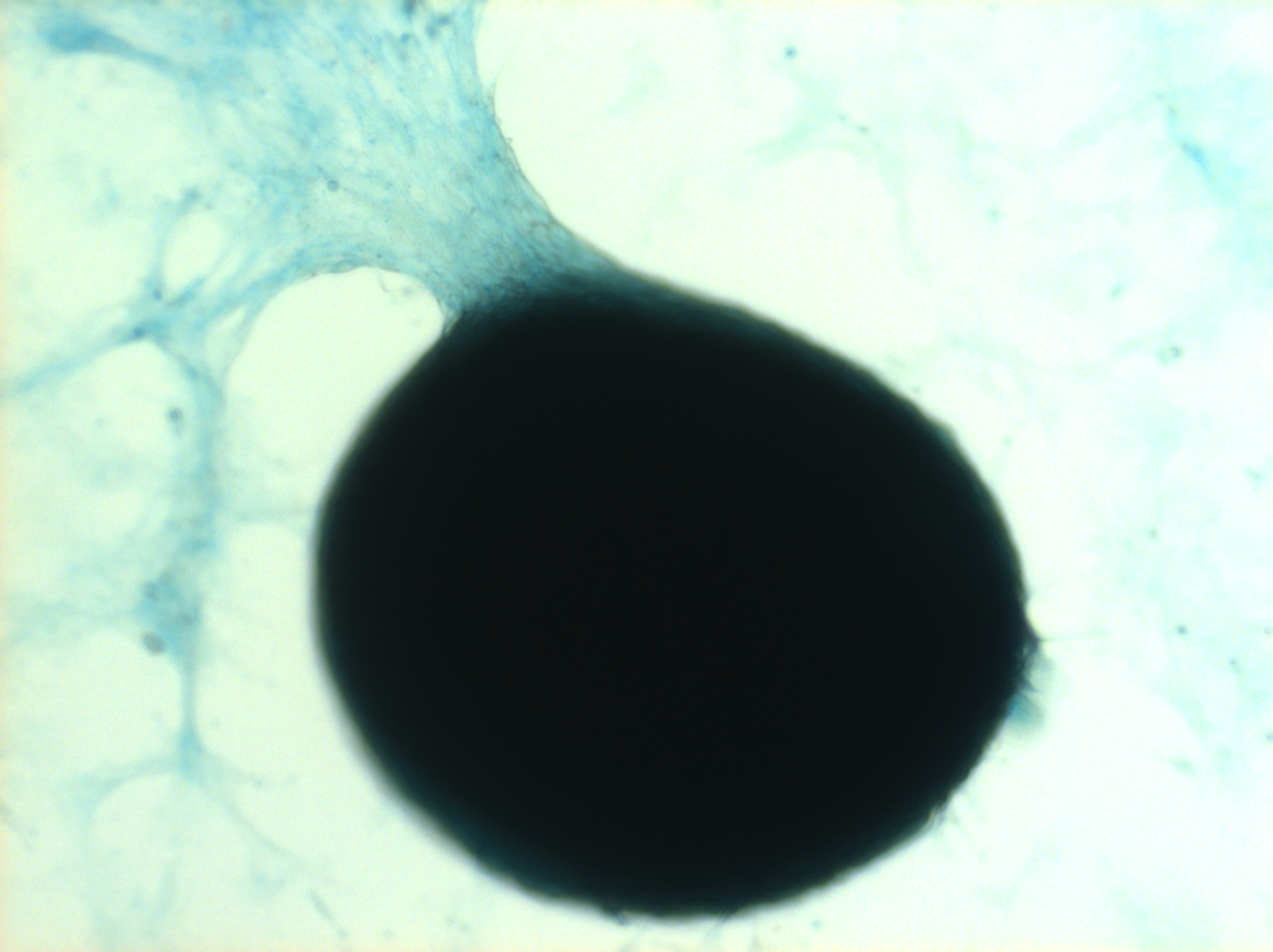

Supplement: S1 Data — Within the Data.zip file, there are 6 folders, whose name corresponds to each individual figure of our manuscript. Raw data including microscope and gel images as well as calculations are contained within each of these folders. Fig01 folder contains 2 flow cytometry plots for Figure 1A and 1C in pdf format as well as 12 phase-contrast and fluorescence microscope images for Figures 1B and 1D. Fig02 folder contains an excel sheet for cell counting and cell doubling data for Figure 2A and 2B. Fig03 folder contains 4 oil red o-stained microscope images for Figure 3A, and 1 agarose gel image (PCR data) as well as an excel sheet for calculating adipogenic gene expression data in Figure 3B. Fig04 folder contains 4 phase-contrast microscope images for Figure 4A, 6 alcian blue-stained microscope images for Figure 4B, and 2 agarose gel images (PCR data) as well as an excel sheet for calculating chondrogenic gene expression data in Figure 4C. Fig05 folder contains 4 ALP-stained microscope images for Figure 5A, an excel sheet for quantifying ALP activity in Figure 5B, 4 alizain red-stained microscope images for Figure 5C, an excel sheet for quantifying alizarin red staining in Figure 5D, and 3 agarose gel images (PCR data) as well as an excel sheet for calculating osteogenic gene expression data in Figure 5E. Fig06 folder contains 8 phase-contrast and fluorescence microscope images for Figures 6A and 6B and 2 ALP-stained images for Figure 6C. (ZIP) [file pone.0139054.s001.zip › Data/Fig04 Chondrogenic Data/AB 02b-Set01-05X-C3H10THalfmRuby2-Chondro03.TIF]

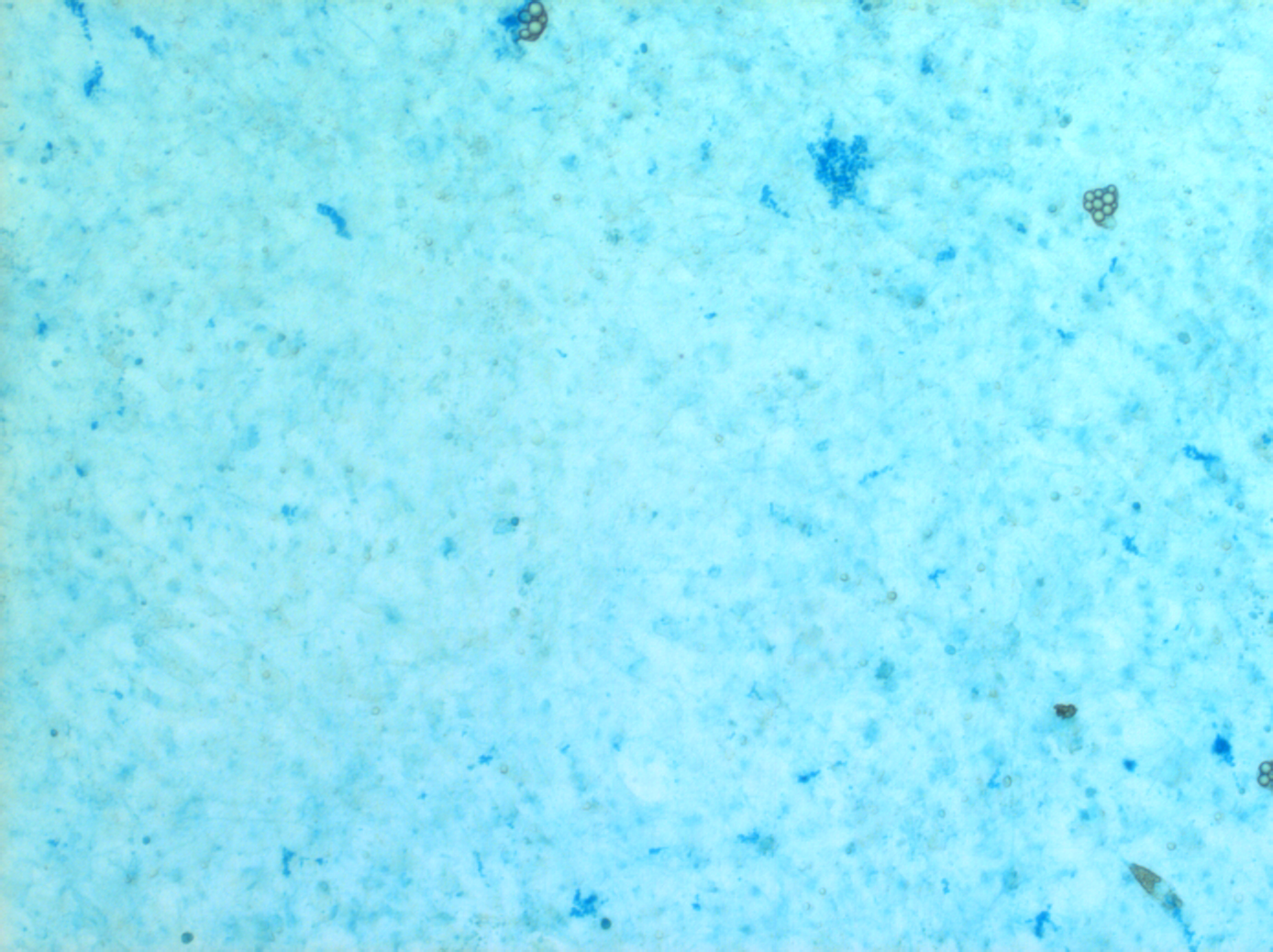

Supplement: S1 Data — Within the Data.zip file, there are 6 folders, whose name corresponds to each individual figure of our manuscript. Raw data including microscope and gel images as well as calculations are contained within each of these folders. Fig01 folder contains 2 flow cytometry plots for Figure 1A and 1C in pdf format as well as 12 phase-contrast and fluorescence microscope images for Figures 1B and 1D. Fig02 folder contains an excel sheet for cell counting and cell doubling data for Figure 2A and 2B. Fig03 folder contains 4 oil red o-stained microscope images for Figure 3A, and 1 agarose gel image (PCR data) as well as an excel sheet for calculating adipogenic gene expression data in Figure 3B. Fig04 folder contains 4 phase-contrast microscope images for Figure 4A, 6 alcian blue-stained microscope images for Figure 4B, and 2 agarose gel images (PCR data) as well as an excel sheet for calculating chondrogenic gene expression data in Figure 4C. Fig05 folder contains 4 ALP-stained microscope images for Figure 5A, an excel sheet for quantifying ALP activity in Figure 5B, 4 alizain red-stained microscope images for Figure 5C, an excel sheet for quantifying alizarin red staining in Figure 5D, and 3 agarose gel images (PCR data) as well as an excel sheet for calculating osteogenic gene expression data in Figure 5E. Fig06 folder contains 8 phase-contrast and fluorescence microscope images for Figures 6A and 6B and 2 ALP-stained images for Figure 6C. (ZIP) [file pone.0139054.s001.zip › Data/Fig04 Chondrogenic Data/AB 03a-Set02-05X-C3H10THalf-Control01.TIF]

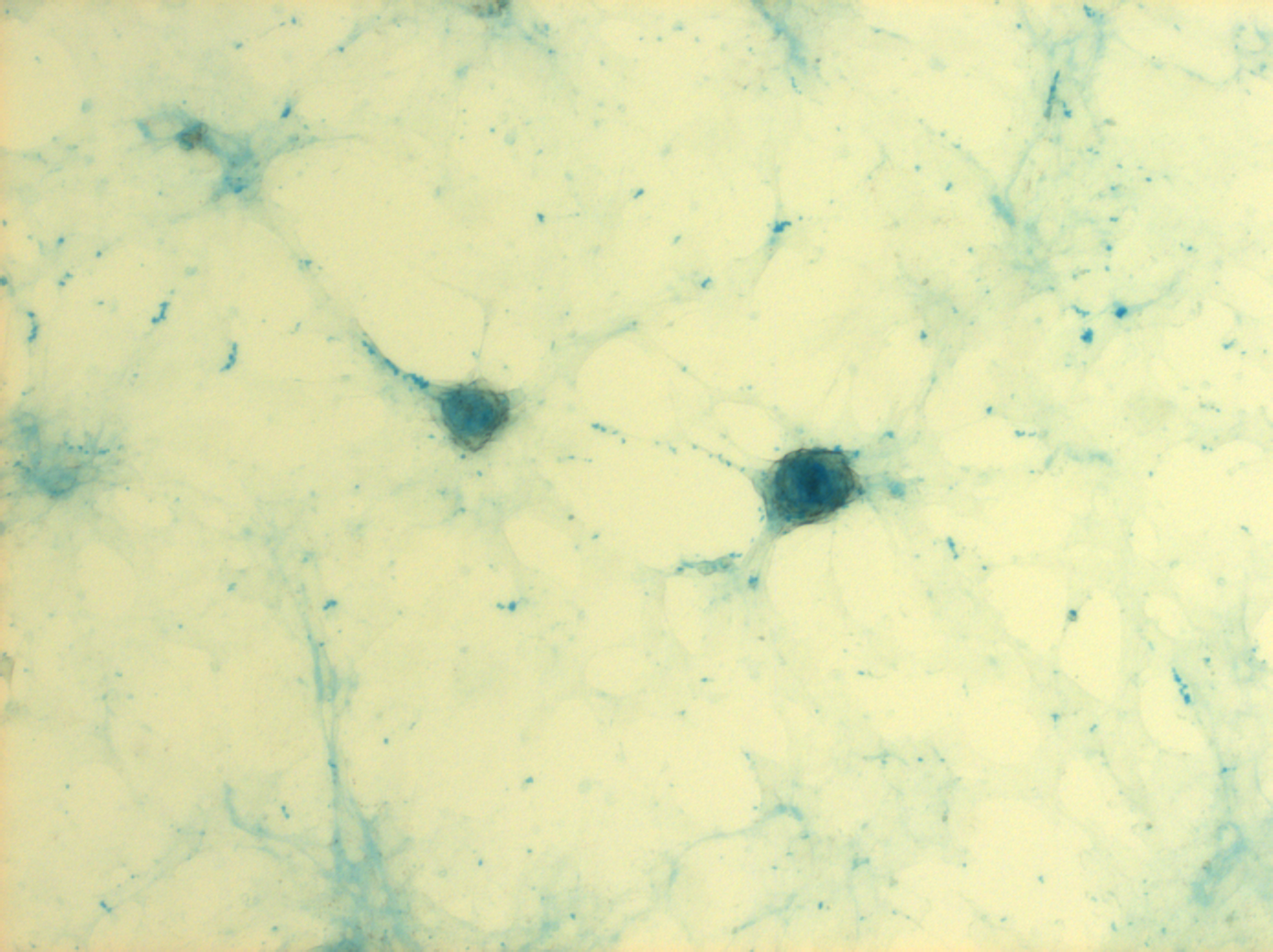

Supplement: S1 Data — Within the Data.zip file, there are 6 folders, whose name corresponds to each individual figure of our manuscript. Raw data including microscope and gel images as well as calculations are contained within each of these folders. Fig01 folder contains 2 flow cytometry plots for Figure 1A and 1C in pdf format as well as 12 phase-contrast and fluorescence microscope images for Figures 1B and 1D. Fig02 folder contains an excel sheet for cell counting and cell doubling data for Figure 2A and 2B. Fig03 folder contains 4 oil red o-stained microscope images for Figure 3A, and 1 agarose gel image (PCR data) as well as an excel sheet for calculating adipogenic gene expression data in Figure 3B. Fig04 folder contains 4 phase-contrast microscope images for Figure 4A, 6 alcian blue-stained microscope images for Figure 4B, and 2 agarose gel images (PCR data) as well as an excel sheet for calculating chondrogenic gene expression data in Figure 4C. Fig05 folder contains 4 ALP-stained microscope images for Figure 5A, an excel sheet for quantifying ALP activity in Figure 5B, 4 alizain red-stained microscope images for Figure 5C, an excel sheet for quantifying alizarin red staining in Figure 5D, and 3 agarose gel images (PCR data) as well as an excel sheet for calculating osteogenic gene expression data in Figure 5E. Fig06 folder contains 8 phase-contrast and fluorescence microscope images for Figures 6A and 6B and 2 ALP-stained images for Figure 6C. (ZIP) [file pone.0139054.s001.zip › Data/Fig04 Chondrogenic Data/AB 06b-Set03-05X-C3H10THalfmRuby2-Chondro03.TIF]

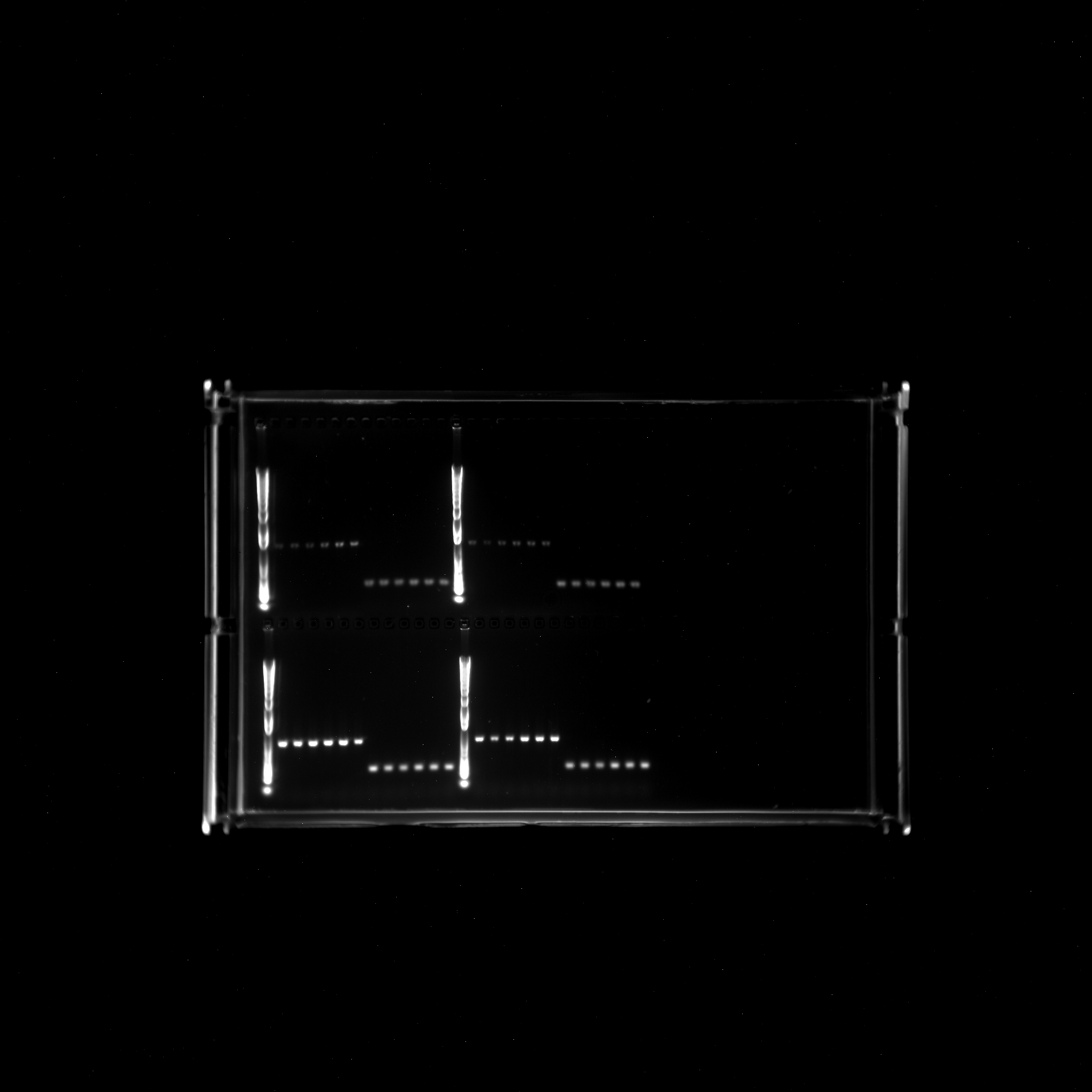

Supplement: S1 Data — Within the Data.zip file, there are 6 folders, whose name corresponds to each individual figure of our manuscript. Raw data including microscope and gel images as well as calculations are contained within each of these folders. Fig01 folder contains 2 flow cytometry plots for Figure 1A and 1C in pdf format as well as 12 phase-contrast and fluorescence microscope images for Figures 1B and 1D. Fig02 folder contains an excel sheet for cell counting and cell doubling data for Figure 2A and 2B. Fig03 folder contains 4 oil red o-stained microscope images for Figure 3A, and 1 agarose gel image (PCR data) as well as an excel sheet for calculating adipogenic gene expression data in Figure 3B. Fig04 folder contains 4 phase-contrast microscope images for Figure 4A, 6 alcian blue-stained microscope images for Figure 4B, and 2 agarose gel images (PCR data) as well as an excel sheet for calculating chondrogenic gene expression data in Figure 4C. Fig05 folder contains 4 ALP-stained microscope images for Figure 5A, an excel sheet for quantifying ALP activity in Figure 5B, 4 alizain red-stained microscope images for Figure 5C, an excel sheet for quantifying alizarin red staining in Figure 5D, and 3 agarose gel images (PCR data) as well as an excel sheet for calculating osteogenic gene expression data in Figure 5E. Fig06 folder contains 8 phase-contrast and fluorescence microscope images for Figures 6A and 6B and 2 ALP-stained images for Figure 6C. (ZIP) [file pone.0139054.s001.zip › Data/Fig04 Chondrogenic Data/PCR Col2 Data.tif]

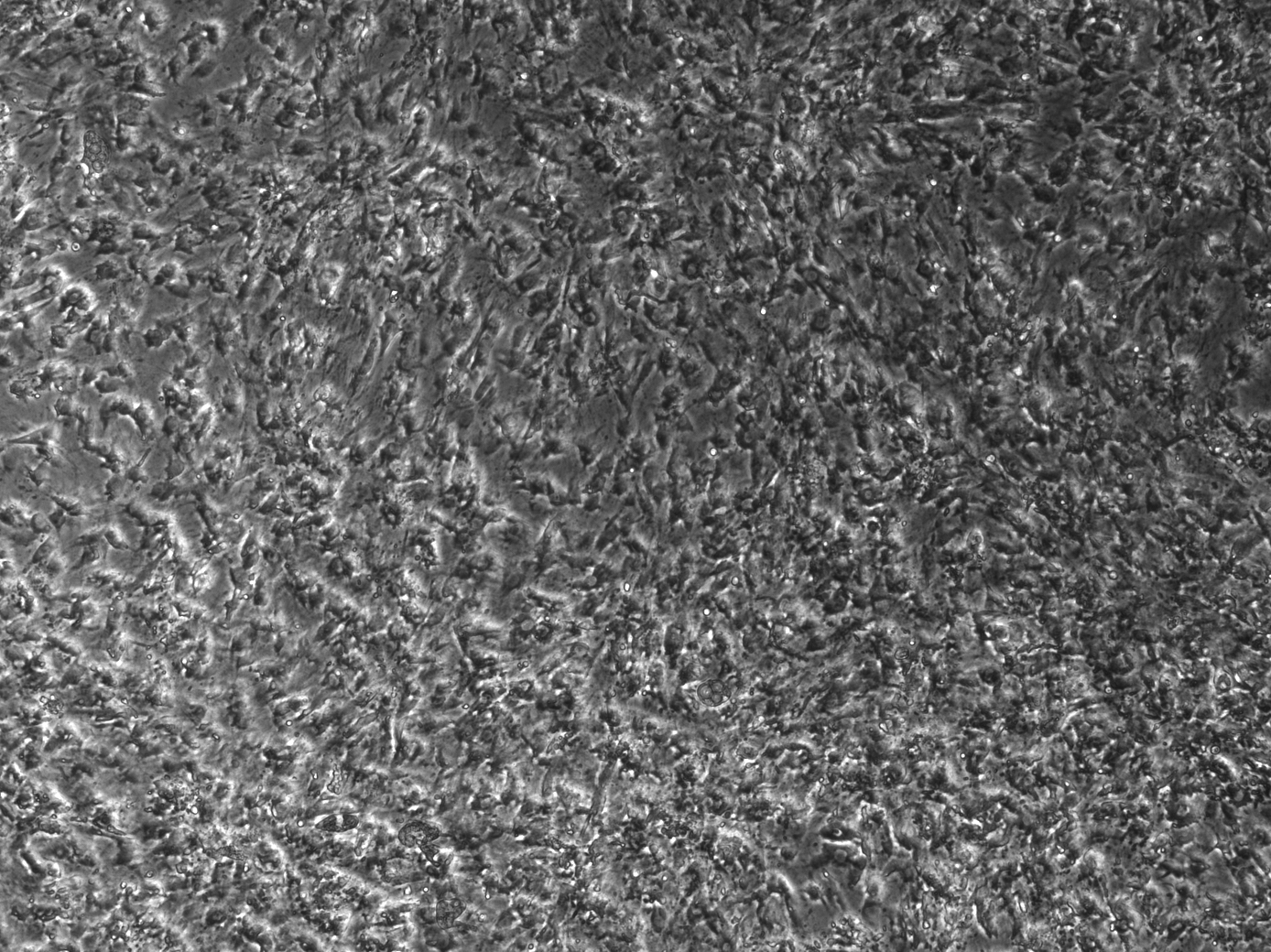

Supplement: S1 Data — Within the Data.zip file, there are 6 folders, whose name corresponds to each individual figure of our manuscript. Raw data including microscope and gel images as well as calculations are contained within each of these folders. Fig01 folder contains 2 flow cytometry plots for Figure 1A and 1C in pdf format as well as 12 phase-contrast and fluorescence microscope images for Figures 1B and 1D. Fig02 folder contains an excel sheet for cell counting and cell doubling data for Figure 2A and 2B. Fig03 folder contains 4 oil red o-stained microscope images for Figure 3A, and 1 agarose gel image (PCR data) as well as an excel sheet for calculating adipogenic gene expression data in Figure 3B. Fig04 folder contains 4 phase-contrast microscope images for Figure 4A, 6 alcian blue-stained microscope images for Figure 4B, and 2 agarose gel images (PCR data) as well as an excel sheet for calculating chondrogenic gene expression data in Figure 4C. Fig05 folder contains 4 ALP-stained microscope images for Figure 5A, an excel sheet for quantifying ALP activity in Figure 5B, 4 alizain red-stained microscope images for Figure 5C, an excel sheet for quantifying alizarin red staining in Figure 5D, and 3 agarose gel images (PCR data) as well as an excel sheet for calculating osteogenic gene expression data in Figure 5E. Fig06 folder contains 8 phase-contrast and fluorescence microscope images for Figures 6A and 6B and 2 ALP-stained images for Figure 6C. (ZIP) [file pone.0139054.s001.zip › Data/Fig04 Chondrogenic Data/Phase03a-Set02-05X-C3H10THalf-Control02.TIF]

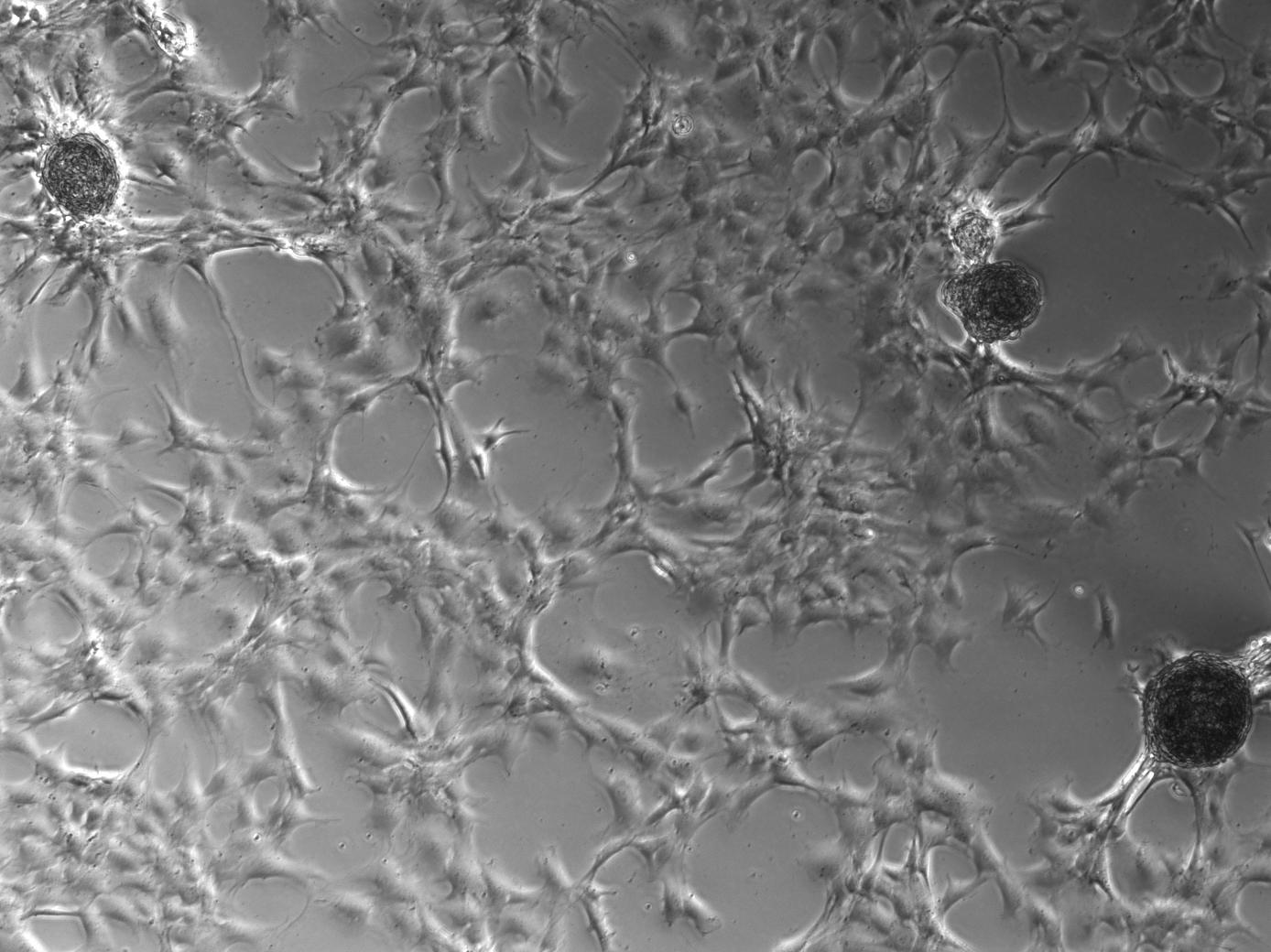

Supplement: S1 Data — Within the Data.zip file, there are 6 folders, whose name corresponds to each individual figure of our manuscript. Raw data including microscope and gel images as well as calculations are contained within each of these folders. Fig01 folder contains 2 flow cytometry plots for Figure 1A and 1C in pdf format as well as 12 phase-contrast and fluorescence microscope images for Figures 1B and 1D. Fig02 folder contains an excel sheet for cell counting and cell doubling data for Figure 2A and 2B. Fig03 folder contains 4 oil red o-stained microscope images for Figure 3A, and 1 agarose gel image (PCR data) as well as an excel sheet for calculating adipogenic gene expression data in Figure 3B. Fig04 folder contains 4 phase-contrast microscope images for Figure 4A, 6 alcian blue-stained microscope images for Figure 4B, and 2 agarose gel images (PCR data) as well as an excel sheet for calculating chondrogenic gene expression data in Figure 4C. Fig05 folder contains 4 ALP-stained microscope images for Figure 5A, an excel sheet for quantifying ALP activity in Figure 5B, 4 alizain red-stained microscope images for Figure 5C, an excel sheet for quantifying alizarin red staining in Figure 5D, and 3 agarose gel images (PCR data) as well as an excel sheet for calculating osteogenic gene expression data in Figure 5E. Fig06 folder contains 8 phase-contrast and fluorescence microscope images for Figures 6A and 6B and 2 ALP-stained images for Figure 6C. (ZIP) [file pone.0139054.s001.zip › Data/Fig04 Chondrogenic Data/Phase-03b-Set02-05X-C3H10THalf-Chondro02.TIF]

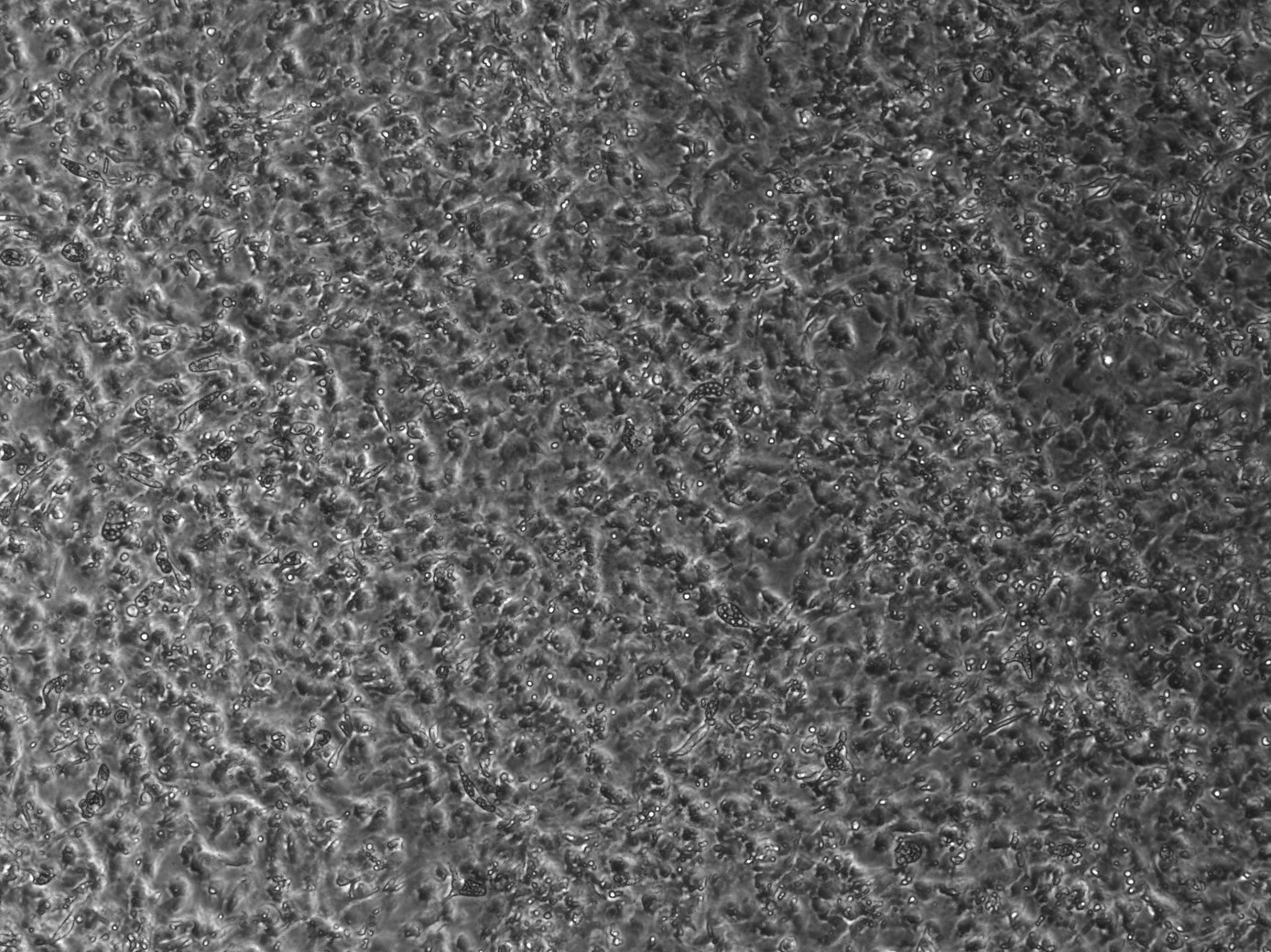

Supplement: S1 Data — Within the Data.zip file, there are 6 folders, whose name corresponds to each individual figure of our manuscript. Raw data including microscope and gel images as well as calculations are contained within each of these folders. Fig01 folder contains 2 flow cytometry plots for Figure 1A and 1C in pdf format as well as 12 phase-contrast and fluorescence microscope images for Figures 1B and 1D. Fig02 folder contains an excel sheet for cell counting and cell doubling data for Figure 2A and 2B. Fig03 folder contains 4 oil red o-stained microscope images for Figure 3A, and 1 agarose gel image (PCR data) as well as an excel sheet for calculating adipogenic gene expression data in Figure 3B. Fig04 folder contains 4 phase-contrast microscope images for Figure 4A, 6 alcian blue-stained microscope images for Figure 4B, and 2 agarose gel images (PCR data) as well as an excel sheet for calculating chondrogenic gene expression data in Figure 4C. Fig05 folder contains 4 ALP-stained microscope images for Figure 5A, an excel sheet for quantifying ALP activity in Figure 5B, 4 alizain red-stained microscope images for Figure 5C, an excel sheet for quantifying alizarin red staining in Figure 5D, and 3 agarose gel images (PCR data) as well as an excel sheet for calculating osteogenic gene expression data in Figure 5E. Fig06 folder contains 8 phase-contrast and fluorescence microscope images for Figures 6A and 6B and 2 ALP-stained images for Figure 6C. (ZIP) [file pone.0139054.s001.zip › Data/Fig04 Chondrogenic Data/Phase04a-Set02-05X-C3H10THalfmRuby2-Control03.TIF]

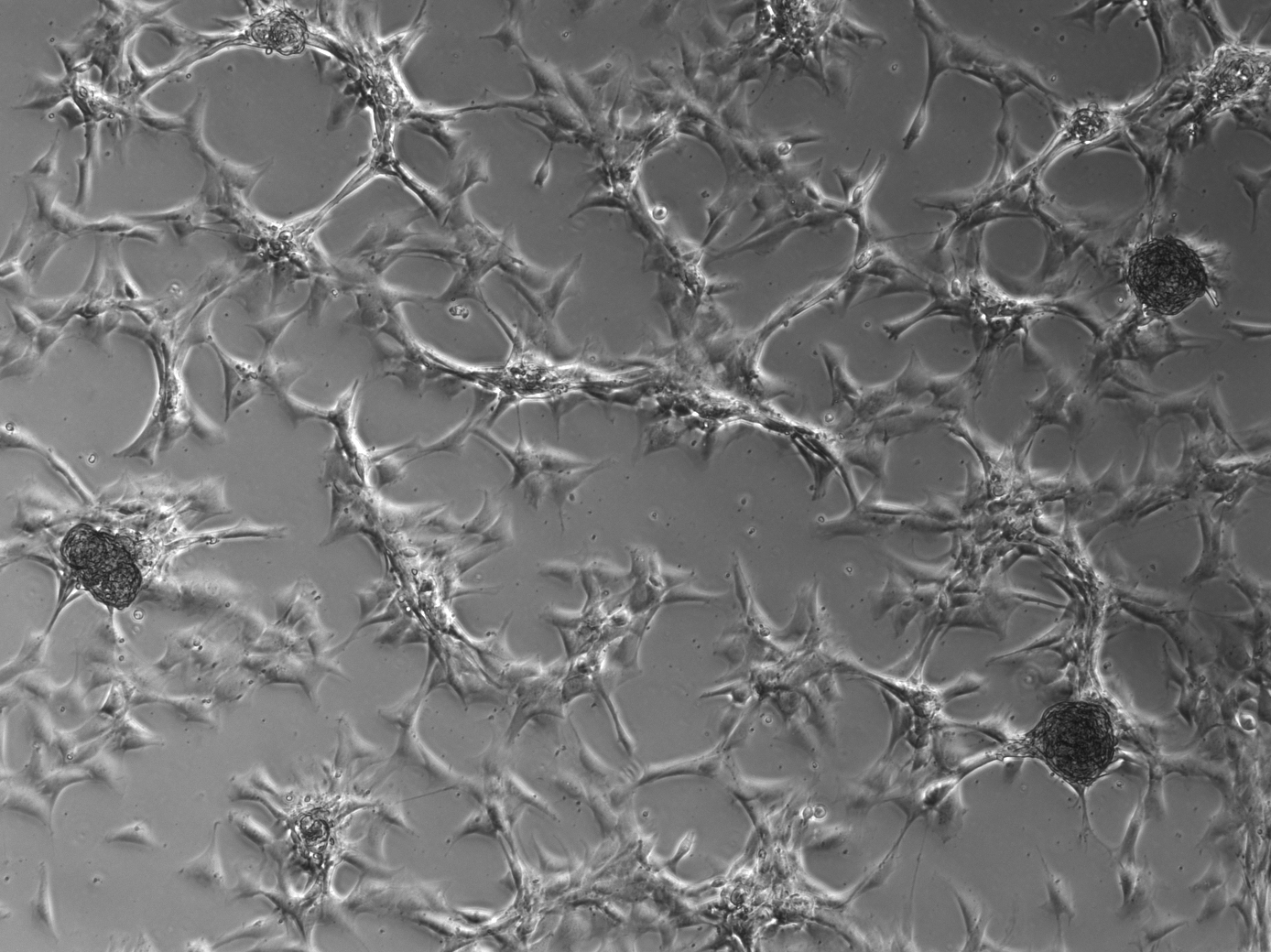

Supplement: S1 Data — Within the Data.zip file, there are 6 folders, whose name corresponds to each individual figure of our manuscript. Raw data including microscope and gel images as well as calculations are contained within each of these folders. Fig01 folder contains 2 flow cytometry plots for Figure 1A and 1C in pdf format as well as 12 phase-contrast and fluorescence microscope images for Figures 1B and 1D. Fig02 folder contains an excel sheet for cell counting and cell doubling data for Figure 2A and 2B. Fig03 folder contains 4 oil red o-stained microscope images for Figure 3A, and 1 agarose gel image (PCR data) as well as an excel sheet for calculating adipogenic gene expression data in Figure 3B. Fig04 folder contains 4 phase-contrast microscope images for Figure 4A, 6 alcian blue-stained microscope images for Figure 4B, and 2 agarose gel images (PCR data) as well as an excel sheet for calculating chondrogenic gene expression data in Figure 4C. Fig05 folder contains 4 ALP-stained microscope images for Figure 5A, an excel sheet for quantifying ALP activity in Figure 5B, 4 alizain red-stained microscope images for Figure 5C, an excel sheet for quantifying alizarin red staining in Figure 5D, and 3 agarose gel images (PCR data) as well as an excel sheet for calculating osteogenic gene expression data in Figure 5E. Fig06 folder contains 8 phase-contrast and fluorescence microscope images for Figures 6A and 6B and 2 ALP-stained images for Figure 6C. (ZIP) [file pone.0139054.s001.zip › Data/Fig04 Chondrogenic Data/Phase04b-Set02-05X-C3H10THalfmRuby2-Chondro02.TIF]

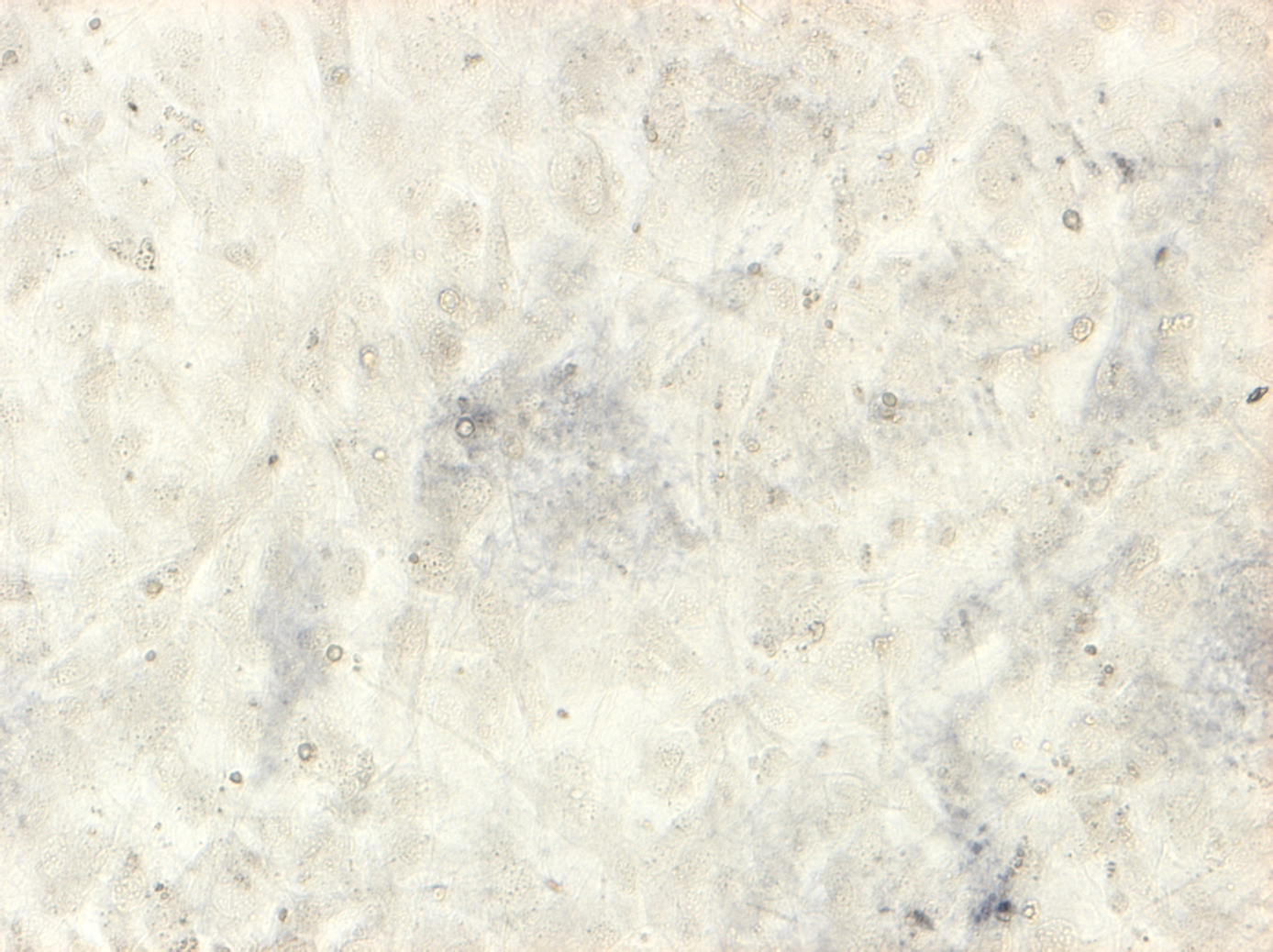

Supplement: S1 Data — Within the Data.zip file, there are 6 folders, whose name corresponds to each individual figure of our manuscript. Raw data including microscope and gel images as well as calculations are contained within each of these folders. Fig01 folder contains 2 flow cytometry plots for Figure 1A and 1C in pdf format as well as 12 phase-contrast and fluorescence microscope images for Figures 1B and 1D. Fig02 folder contains an excel sheet for cell counting and cell doubling data for Figure 2A and 2B. Fig03 folder contains 4 oil red o-stained microscope images for Figure 3A, and 1 agarose gel image (PCR data) as well as an excel sheet for calculating adipogenic gene expression data in Figure 3B. Fig04 folder contains 4 phase-contrast microscope images for Figure 4A, 6 alcian blue-stained microscope images for Figure 4B, and 2 agarose gel images (PCR data) as well as an excel sheet for calculating chondrogenic gene expression data in Figure 4C. Fig05 folder contains 4 ALP-stained microscope images for Figure 5A, an excel sheet for quantifying ALP activity in Figure 5B, 4 alizain red-stained microscope images for Figure 5C, an excel sheet for quantifying alizarin red staining in Figure 5D, and 3 agarose gel images (PCR data) as well as an excel sheet for calculating osteogenic gene expression data in Figure 5E. Fig06 folder contains 8 phase-contrast and fluorescence microscope images for Figures 6A and 6B and 2 ALP-stained images for Figure 6C. (ZIP) [file pone.0139054.s001.zip › Data/Fig05 Osteogenic Data/ALP 01-C3H10THalfP12-000ngpermLBMP2-03.TIF]

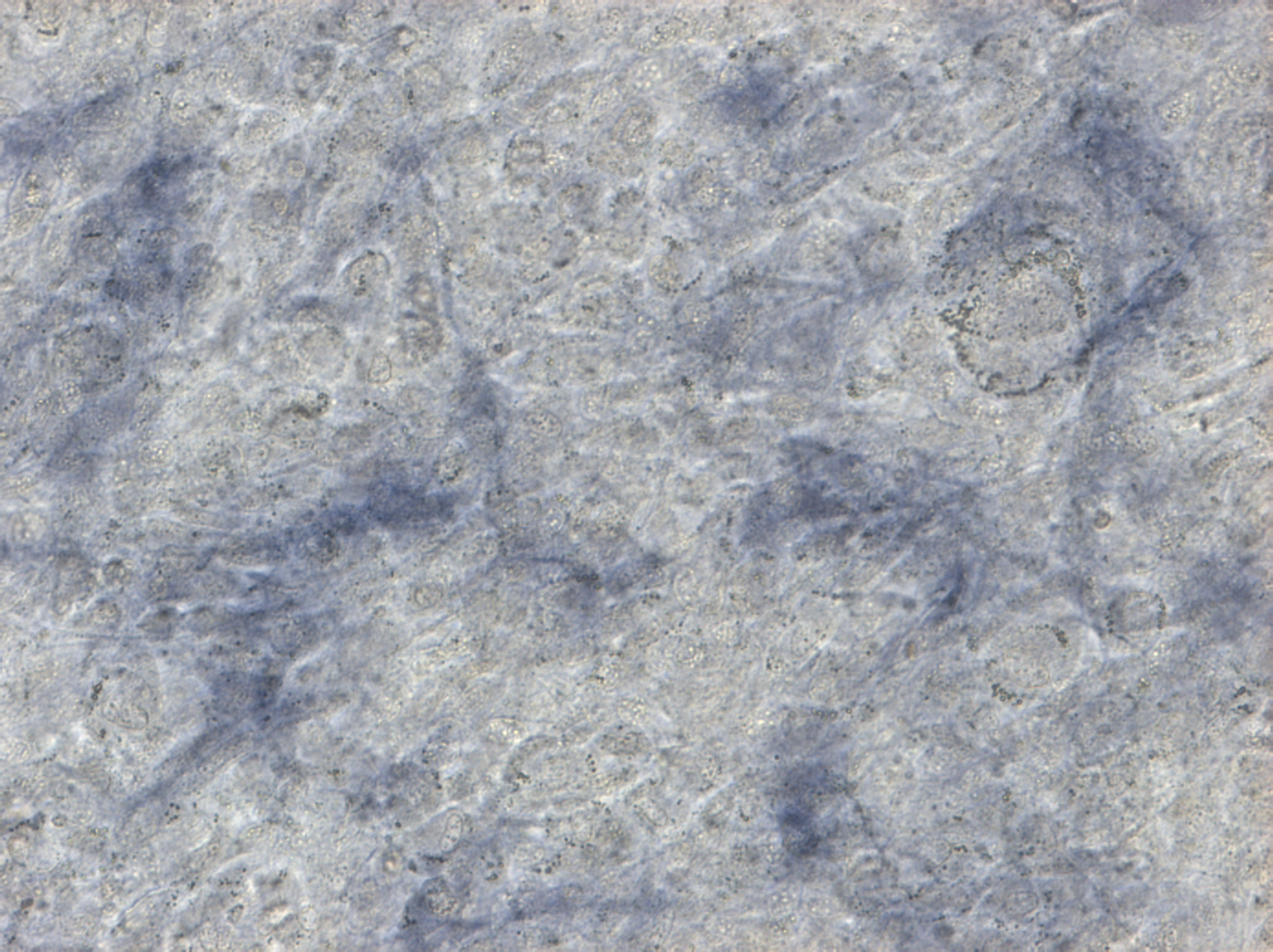

Supplement: S1 Data — Within the Data.zip file, there are 6 folders, whose name corresponds to each individual figure of our manuscript. Raw data including microscope and gel images as well as calculations are contained within each of these folders. Fig01 folder contains 2 flow cytometry plots for Figure 1A and 1C in pdf format as well as 12 phase-contrast and fluorescence microscope images for Figures 1B and 1D. Fig02 folder contains an excel sheet for cell counting and cell doubling data for Figure 2A and 2B. Fig03 folder contains 4 oil red o-stained microscope images for Figure 3A, and 1 agarose gel image (PCR data) as well as an excel sheet for calculating adipogenic gene expression data in Figure 3B. Fig04 folder contains 4 phase-contrast microscope images for Figure 4A, 6 alcian blue-stained microscope images for Figure 4B, and 2 agarose gel images (PCR data) as well as an excel sheet for calculating chondrogenic gene expression data in Figure 4C. Fig05 folder contains 4 ALP-stained microscope images for Figure 5A, an excel sheet for quantifying ALP activity in Figure 5B, 4 alizain red-stained microscope images for Figure 5C, an excel sheet for quantifying alizarin red staining in Figure 5D, and 3 agarose gel images (PCR data) as well as an excel sheet for calculating osteogenic gene expression data in Figure 5E. Fig06 folder contains 8 phase-contrast and fluorescence microscope images for Figures 6A and 6B and 2 ALP-stained images for Figure 6C. (ZIP) [file pone.0139054.s001.zip › Data/Fig05 Osteogenic Data/ALP 02-C3H10THalfP12-100ngpermLBMP2-02.TIF]

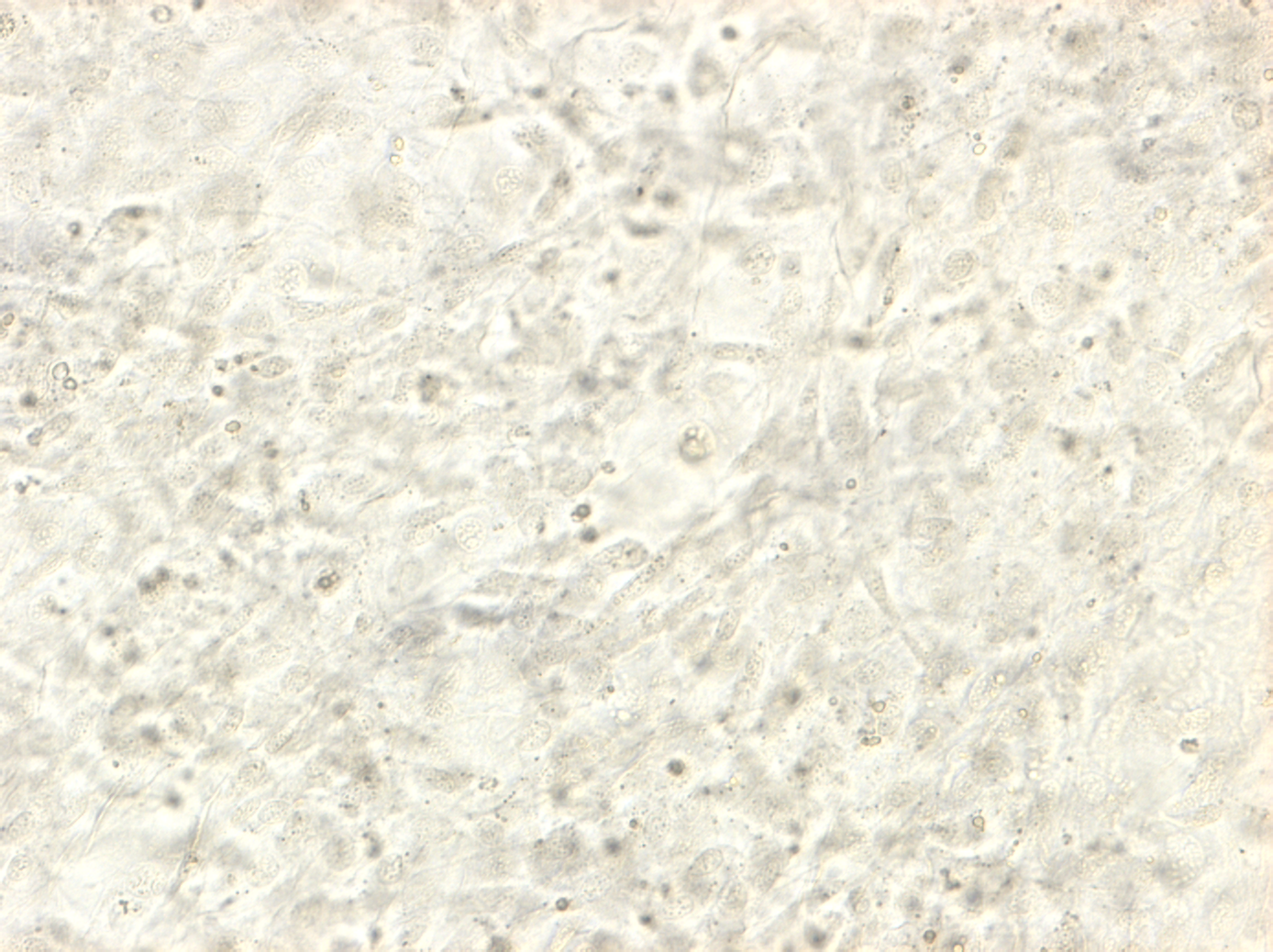

Supplement: S1 Data — Within the Data.zip file, there are 6 folders, whose name corresponds to each individual figure of our manuscript. Raw data including microscope and gel images as well as calculations are contained within each of these folders. Fig01 folder contains 2 flow cytometry plots for Figure 1A and 1C in pdf format as well as 12 phase-contrast and fluorescence microscope images for Figures 1B and 1D. Fig02 folder contains an excel sheet for cell counting and cell doubling data for Figure 2A and 2B. Fig03 folder contains 4 oil red o-stained microscope images for Figure 3A, and 1 agarose gel image (PCR data) as well as an excel sheet for calculating adipogenic gene expression data in Figure 3B. Fig04 folder contains 4 phase-contrast microscope images for Figure 4A, 6 alcian blue-stained microscope images for Figure 4B, and 2 agarose gel images (PCR data) as well as an excel sheet for calculating chondrogenic gene expression data in Figure 4C. Fig05 folder contains 4 ALP-stained microscope images for Figure 5A, an excel sheet for quantifying ALP activity in Figure 5B, 4 alizain red-stained microscope images for Figure 5C, an excel sheet for quantifying alizarin red staining in Figure 5D, and 3 agarose gel images (PCR data) as well as an excel sheet for calculating osteogenic gene expression data in Figure 5E. Fig06 folder contains 8 phase-contrast and fluorescence microscope images for Figures 6A and 6B and 2 ALP-stained images for Figure 6C. (ZIP) [file pone.0139054.s001.zip › Data/Fig05 Osteogenic Data/ALP 03-C3H10THalfpVitro2mRuby2BlastP22-000ngpermLBMP2-04.TIF]

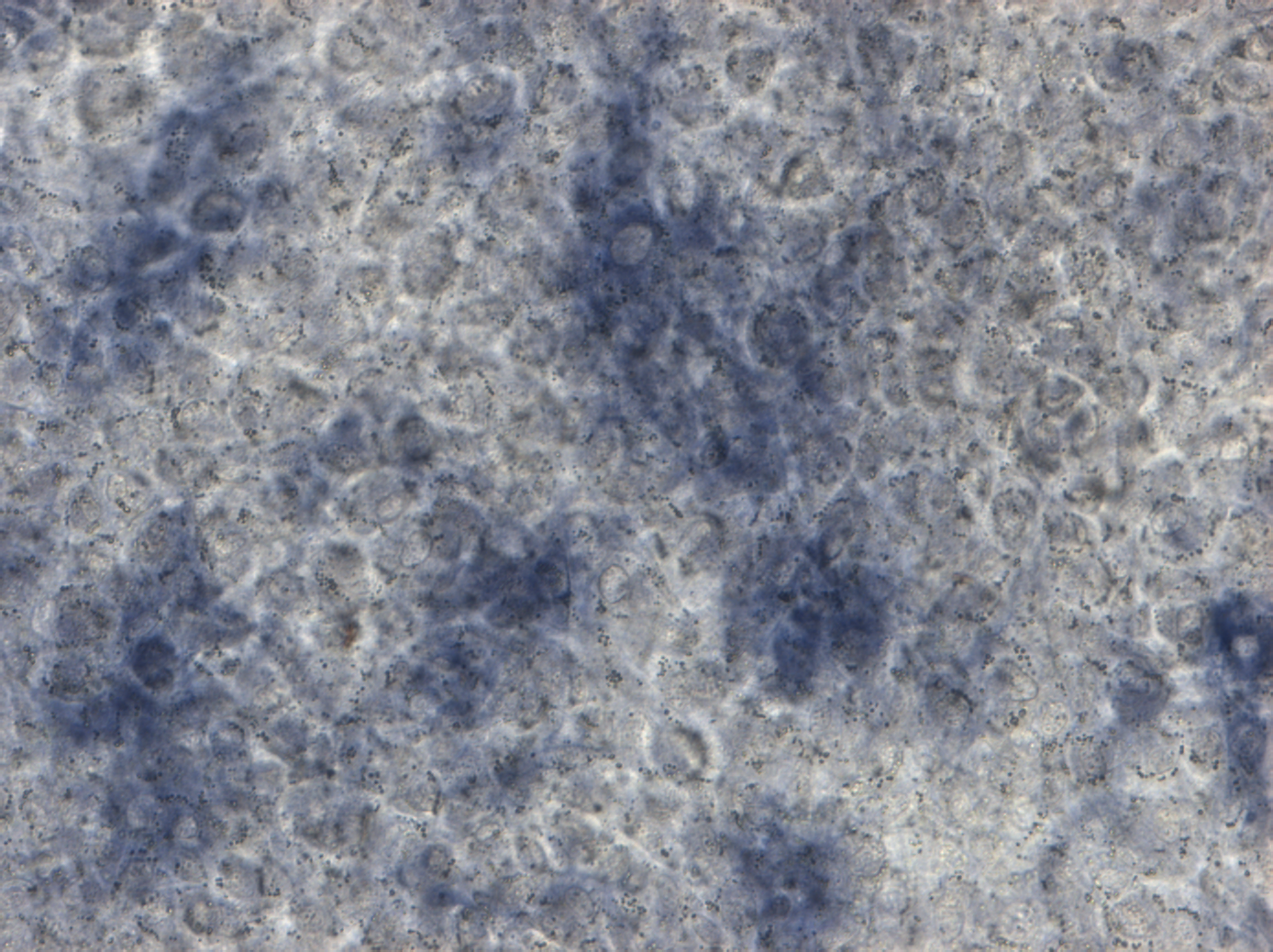

Supplement: S1 Data — Within the Data.zip file, there are 6 folders, whose name corresponds to each individual figure of our manuscript. Raw data including microscope and gel images as well as calculations are contained within each of these folders. Fig01 folder contains 2 flow cytometry plots for Figure 1A and 1C in pdf format as well as 12 phase-contrast and fluorescence microscope images for Figures 1B and 1D. Fig02 folder contains an excel sheet for cell counting and cell doubling data for Figure 2A and 2B. Fig03 folder contains 4 oil red o-stained microscope images for Figure 3A, and 1 agarose gel image (PCR data) as well as an excel sheet for calculating adipogenic gene expression data in Figure 3B. Fig04 folder contains 4 phase-contrast microscope images for Figure 4A, 6 alcian blue-stained microscope images for Figure 4B, and 2 agarose gel images (PCR data) as well as an excel sheet for calculating chondrogenic gene expression data in Figure 4C. Fig05 folder contains 4 ALP-stained microscope images for Figure 5A, an excel sheet for quantifying ALP activity in Figure 5B, 4 alizain red-stained microscope images for Figure 5C, an excel sheet for quantifying alizarin red staining in Figure 5D, and 3 agarose gel images (PCR data) as well as an excel sheet for calculating osteogenic gene expression data in Figure 5E. Fig06 folder contains 8 phase-contrast and fluorescence microscope images for Figures 6A and 6B and 2 ALP-stained images for Figure 6C. (ZIP) [file pone.0139054.s001.zip › Data/Fig05 Osteogenic Data/ALP 04-C3H10THalfpVitro2mRuby2BlastP22-100ngpermLBMP2-01.TIF]

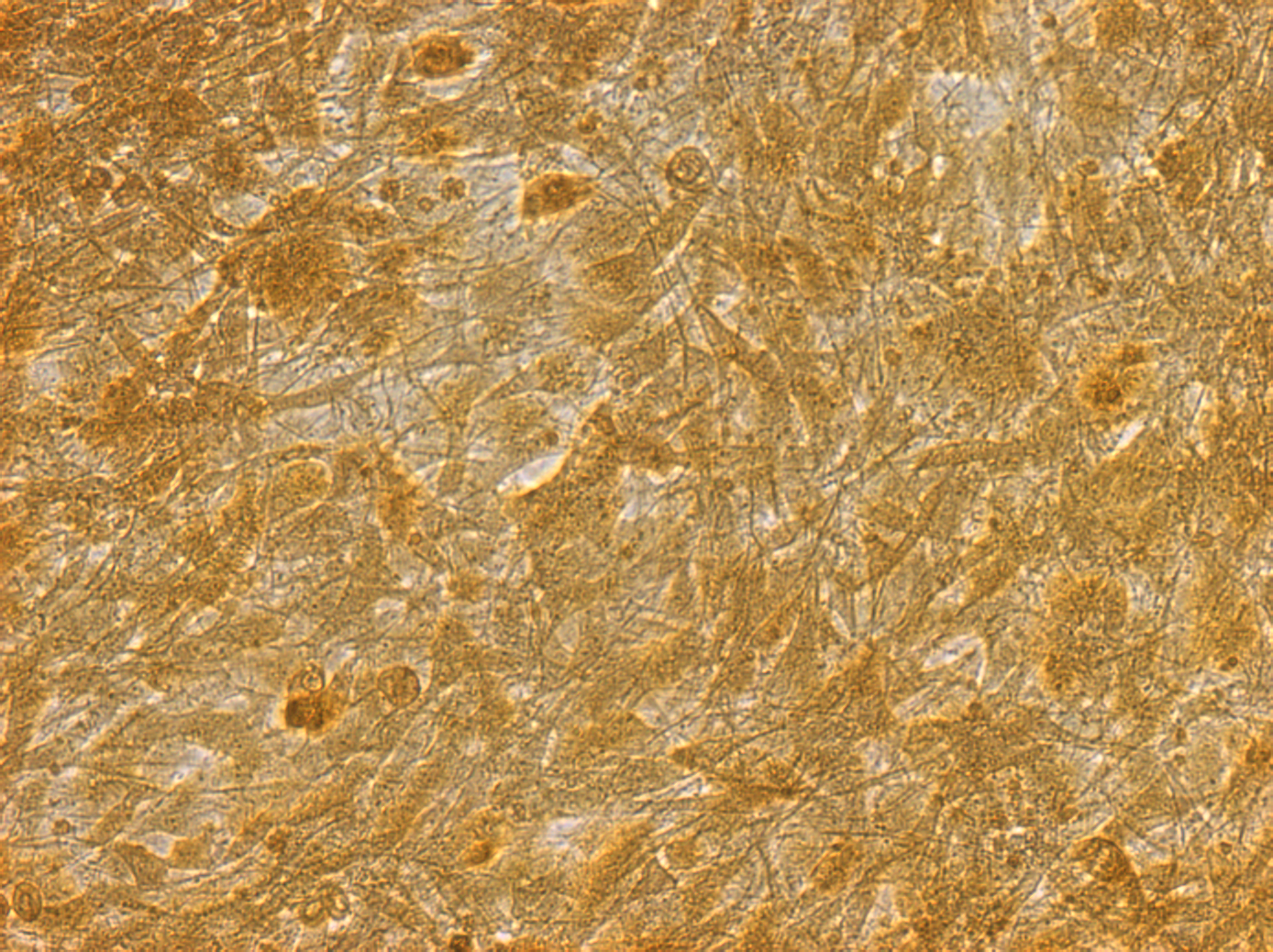

Supplement: S1 Data — Within the Data.zip file, there are 6 folders, whose name corresponds to each individual figure of our manuscript. Raw data including microscope and gel images as well as calculations are contained within each of these folders. Fig01 folder contains 2 flow cytometry plots for Figure 1A and 1C in pdf format as well as 12 phase-contrast and fluorescence microscope images for Figures 1B and 1D. Fig02 folder contains an excel sheet for cell counting and cell doubling data for Figure 2A and 2B. Fig03 folder contains 4 oil red o-stained microscope images for Figure 3A, and 1 agarose gel image (PCR data) as well as an excel sheet for calculating adipogenic gene expression data in Figure 3B. Fig04 folder contains 4 phase-contrast microscope images for Figure 4A, 6 alcian blue-stained microscope images for Figure 4B, and 2 agarose gel images (PCR data) as well as an excel sheet for calculating chondrogenic gene expression data in Figure 4C. Fig05 folder contains 4 ALP-stained microscope images for Figure 5A, an excel sheet for quantifying ALP activity in Figure 5B, 4 alizain red-stained microscope images for Figure 5C, an excel sheet for quantifying alizarin red staining in Figure 5D, and 3 agarose gel images (PCR data) as well as an excel sheet for calculating osteogenic gene expression data in Figure 5E. Fig06 folder contains 8 phase-contrast and fluorescence microscope images for Figures 6A and 6B and 2 ALP-stained images for Figure 6C. (ZIP) [file pone.0139054.s001.zip › Data/Fig05 Osteogenic Data/ARS 01-10X-C3H10THalfUntransfected-Control02.TIF]

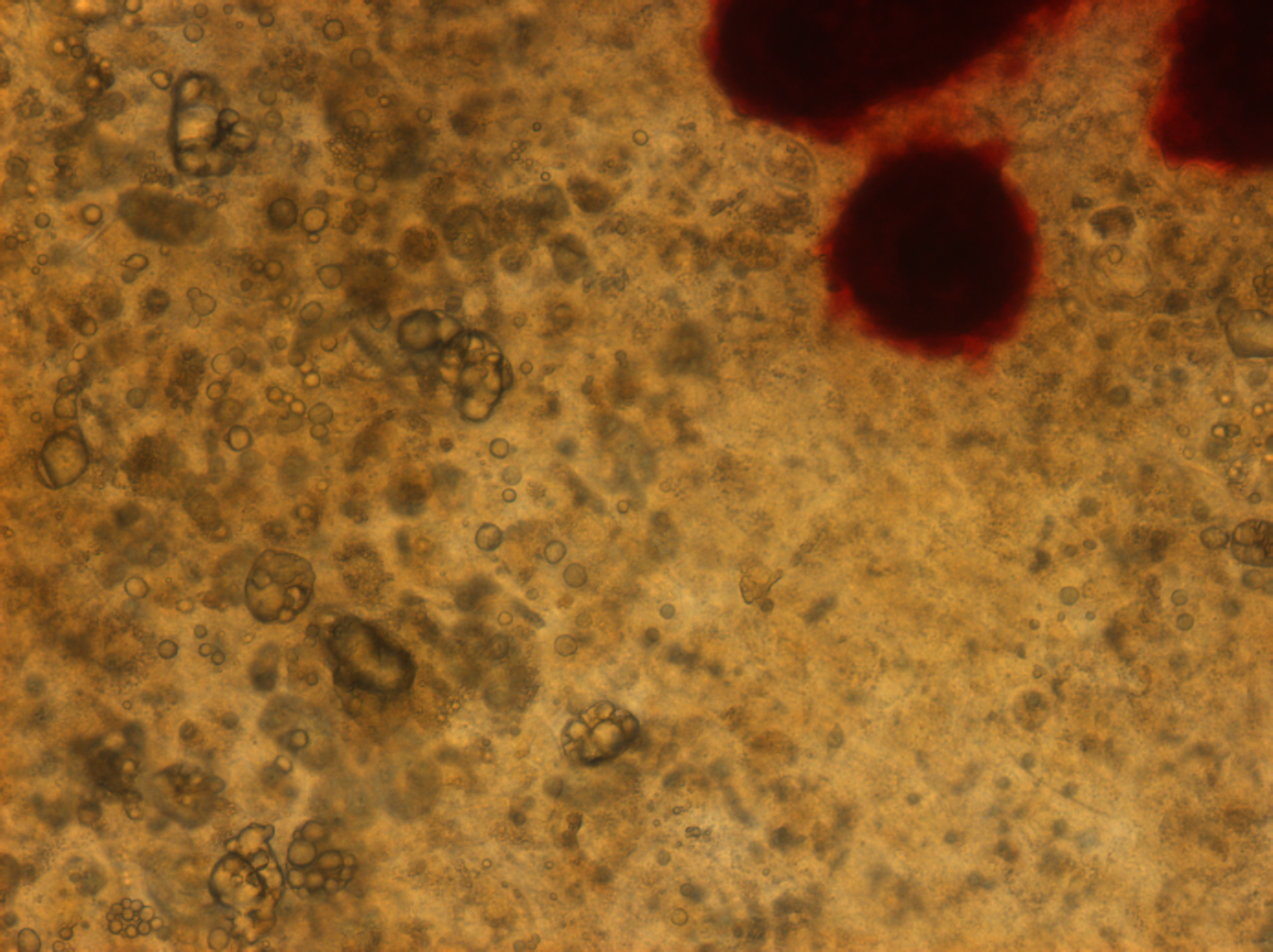

Supplement: S1 Data — Within the Data.zip file, there are 6 folders, whose name corresponds to each individual figure of our manuscript. Raw data including microscope and gel images as well as calculations are contained within each of these folders. Fig01 folder contains 2 flow cytometry plots for Figure 1A and 1C in pdf format as well as 12 phase-contrast and fluorescence microscope images for Figures 1B and 1D. Fig02 folder contains an excel sheet for cell counting and cell doubling data for Figure 2A and 2B. Fig03 folder contains 4 oil red o-stained microscope images for Figure 3A, and 1 agarose gel image (PCR data) as well as an excel sheet for calculating adipogenic gene expression data in Figure 3B. Fig04 folder contains 4 phase-contrast microscope images for Figure 4A, 6 alcian blue-stained microscope images for Figure 4B, and 2 agarose gel images (PCR data) as well as an excel sheet for calculating chondrogenic gene expression data in Figure 4C. Fig05 folder contains 4 ALP-stained microscope images for Figure 5A, an excel sheet for quantifying ALP activity in Figure 5B, 4 alizain red-stained microscope images for Figure 5C, an excel sheet for quantifying alizarin red staining in Figure 5D, and 3 agarose gel images (PCR data) as well as an excel sheet for calculating osteogenic gene expression data in Figure 5E. Fig06 folder contains 8 phase-contrast and fluorescence microscope images for Figures 6A and 6B and 2 ALP-stained images for Figure 6C. (ZIP) [file pone.0139054.s001.zip › Data/Fig05 Osteogenic Data/ARS 01-C3H10THalfMin27Days-100ngpermLBMP2-05.TIF]

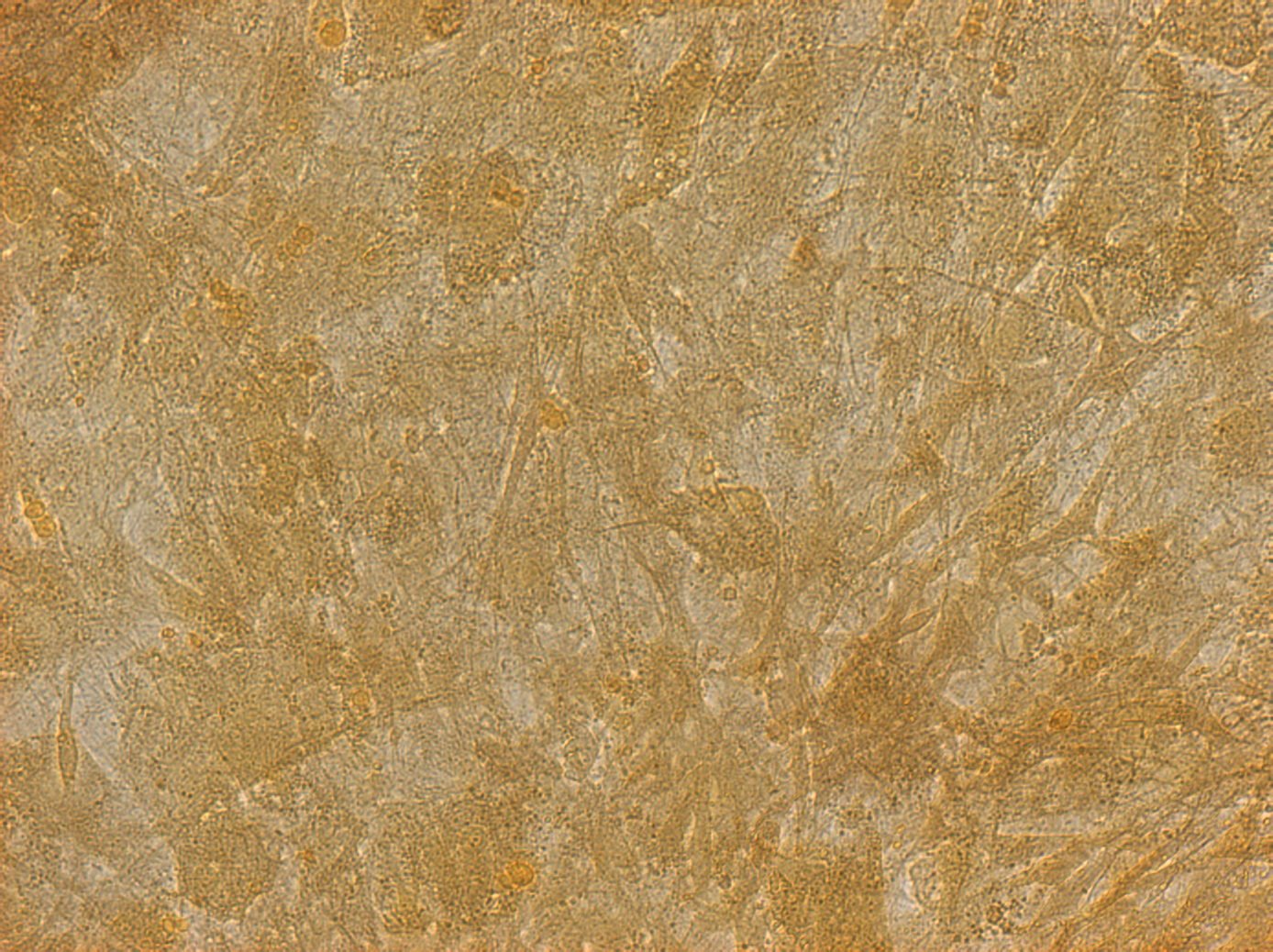

Supplement: S1 Data — Within the Data.zip file, there are 6 folders, whose name corresponds to each individual figure of our manuscript. Raw data including microscope and gel images as well as calculations are contained within each of these folders. Fig01 folder contains 2 flow cytometry plots for Figure 1A and 1C in pdf format as well as 12 phase-contrast and fluorescence microscope images for Figures 1B and 1D. Fig02 folder contains an excel sheet for cell counting and cell doubling data for Figure 2A and 2B. Fig03 folder contains 4 oil red o-stained microscope images for Figure 3A, and 1 agarose gel image (PCR data) as well as an excel sheet for calculating adipogenic gene expression data in Figure 3B. Fig04 folder contains 4 phase-contrast microscope images for Figure 4A, 6 alcian blue-stained microscope images for Figure 4B, and 2 agarose gel images (PCR data) as well as an excel sheet for calculating chondrogenic gene expression data in Figure 4C. Fig05 folder contains 4 ALP-stained microscope images for Figure 5A, an excel sheet for quantifying ALP activity in Figure 5B, 4 alizain red-stained microscope images for Figure 5C, an excel sheet for quantifying alizarin red staining in Figure 5D, and 3 agarose gel images (PCR data) as well as an excel sheet for calculating osteogenic gene expression data in Figure 5E. Fig06 folder contains 8 phase-contrast and fluorescence microscope images for Figures 6A and 6B and 2 ALP-stained images for Figure 6C. (ZIP) [file pone.0139054.s001.zip › Data/Fig05 Osteogenic Data/ARS 03-10X-C3H10THalfmRuby2-Control03.TIF]

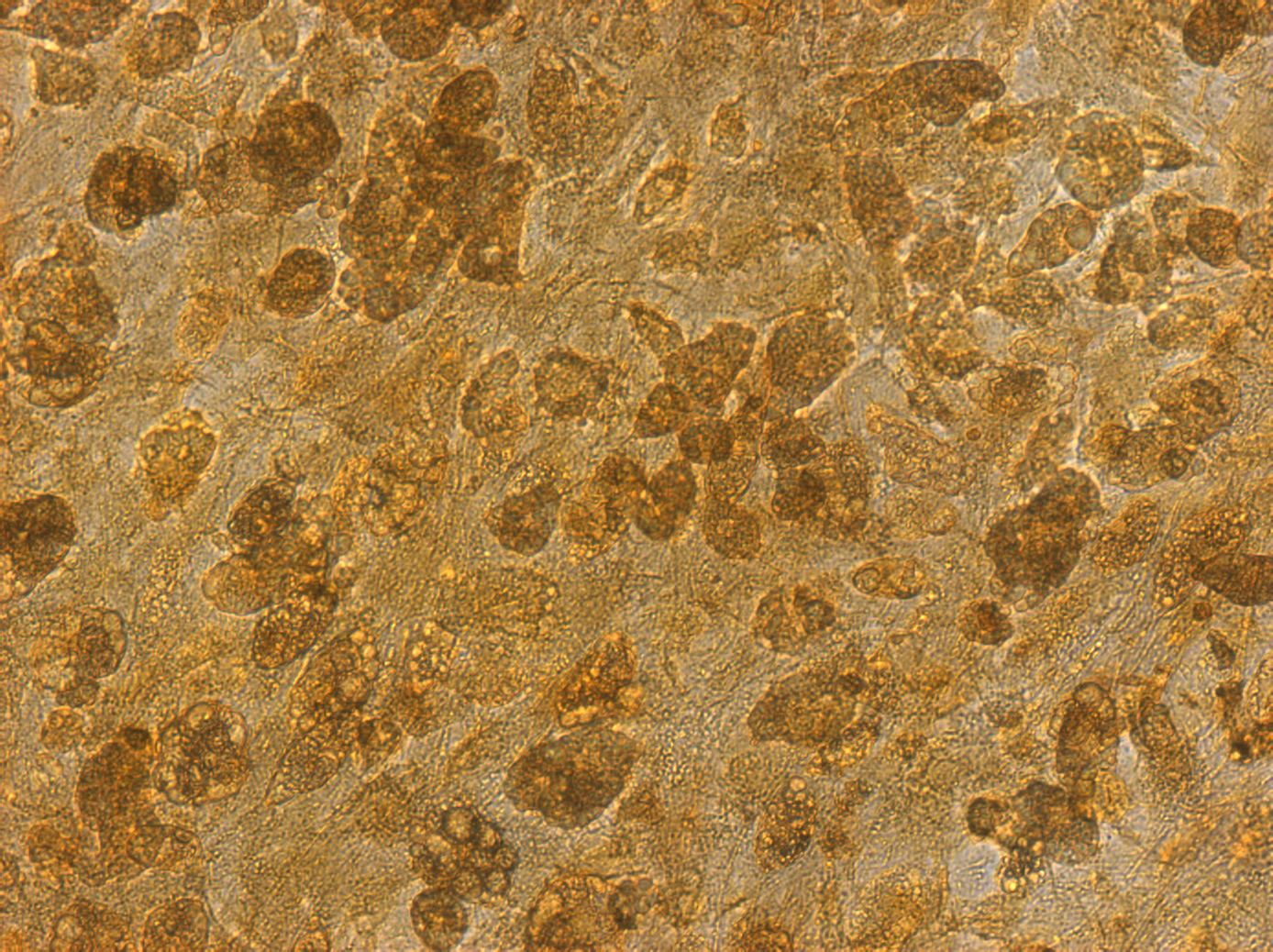

Supplement: S1 Data — Within the Data.zip file, there are 6 folders, whose name corresponds to each individual figure of our manuscript. Raw data including microscope and gel images as well as calculations are contained within each of these folders. Fig01 folder contains 2 flow cytometry plots for Figure 1A and 1C in pdf format as well as 12 phase-contrast and fluorescence microscope images for Figures 1B and 1D. Fig02 folder contains an excel sheet for cell counting and cell doubling data for Figure 2A and 2B. Fig03 folder contains 4 oil red o-stained microscope images for Figure 3A, and 1 agarose gel image (PCR data) as well as an excel sheet for calculating adipogenic gene expression data in Figure 3B. Fig04 folder contains 4 phase-contrast microscope images for Figure 4A, 6 alcian blue-stained microscope images for Figure 4B, and 2 agarose gel images (PCR data) as well as an excel sheet for calculating chondrogenic gene expression data in Figure 4C. Fig05 folder contains 4 ALP-stained microscope images for Figure 5A, an excel sheet for quantifying ALP activity in Figure 5B, 4 alizain red-stained microscope images for Figure 5C, an excel sheet for quantifying alizarin red staining in Figure 5D, and 3 agarose gel images (PCR data) as well as an excel sheet for calculating osteogenic gene expression data in Figure 5E. Fig06 folder contains 8 phase-contrast and fluorescence microscope images for Figures 6A and 6B and 2 ALP-stained images for Figure 6C. (ZIP) [file pone.0139054.s001.zip › Data/Fig05 Osteogenic Data/ARS 04-10X-C3H10THalfmRuby2-100ngpermLBMP2-03.TIF]

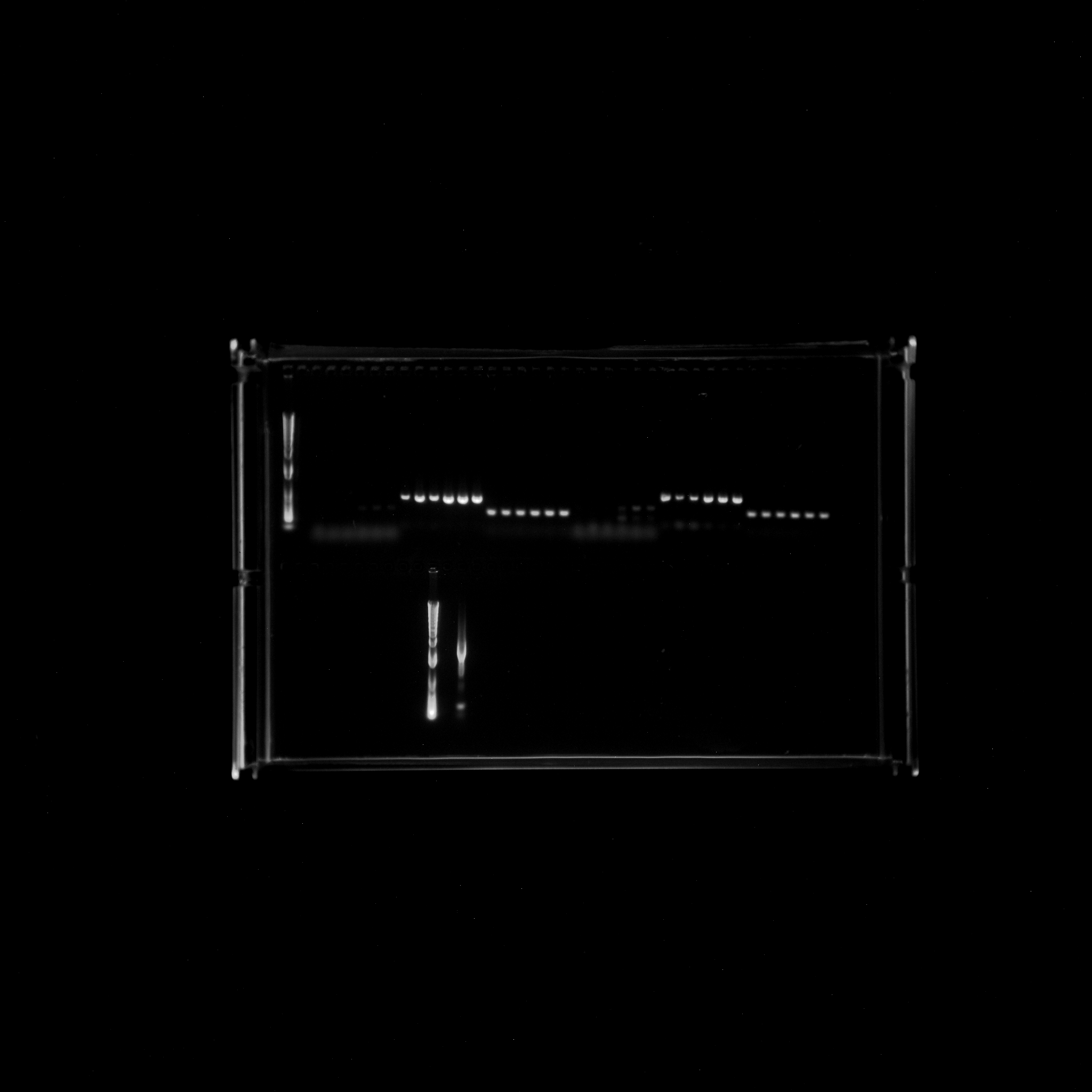

Supplement: S1 Data — Within the Data.zip file, there are 6 folders, whose name corresponds to each individual figure of our manuscript. Raw data including microscope and gel images as well as calculations are contained within each of these folders. Fig01 folder contains 2 flow cytometry plots for Figure 1A and 1C in pdf format as well as 12 phase-contrast and fluorescence microscope images for Figures 1B and 1D. Fig02 folder contains an excel sheet for cell counting and cell doubling data for Figure 2A and 2B. Fig03 folder contains 4 oil red o-stained microscope images for Figure 3A, and 1 agarose gel image (PCR data) as well as an excel sheet for calculating adipogenic gene expression data in Figure 3B. Fig04 folder contains 4 phase-contrast microscope images for Figure 4A, 6 alcian blue-stained microscope images for Figure 4B, and 2 agarose gel images (PCR data) as well as an excel sheet for calculating chondrogenic gene expression data in Figure 4C. Fig05 folder contains 4 ALP-stained microscope images for Figure 5A, an excel sheet for quantifying ALP activity in Figure 5B, 4 alizain red-stained microscope images for Figure 5C, an excel sheet for quantifying alizarin red staining in Figure 5D, and 3 agarose gel images (PCR data) as well as an excel sheet for calculating osteogenic gene expression data in Figure 5E. Fig06 folder contains 8 phase-contrast and fluorescence microscope images for Figures 6A and 6B and 2 ALP-stained images for Figure 6C. (ZIP) [file pone.0139054.s001.zip › Data/Fig05 Osteogenic Data/PCR Ocn,Osf-1-02p5s-Levels.tif]

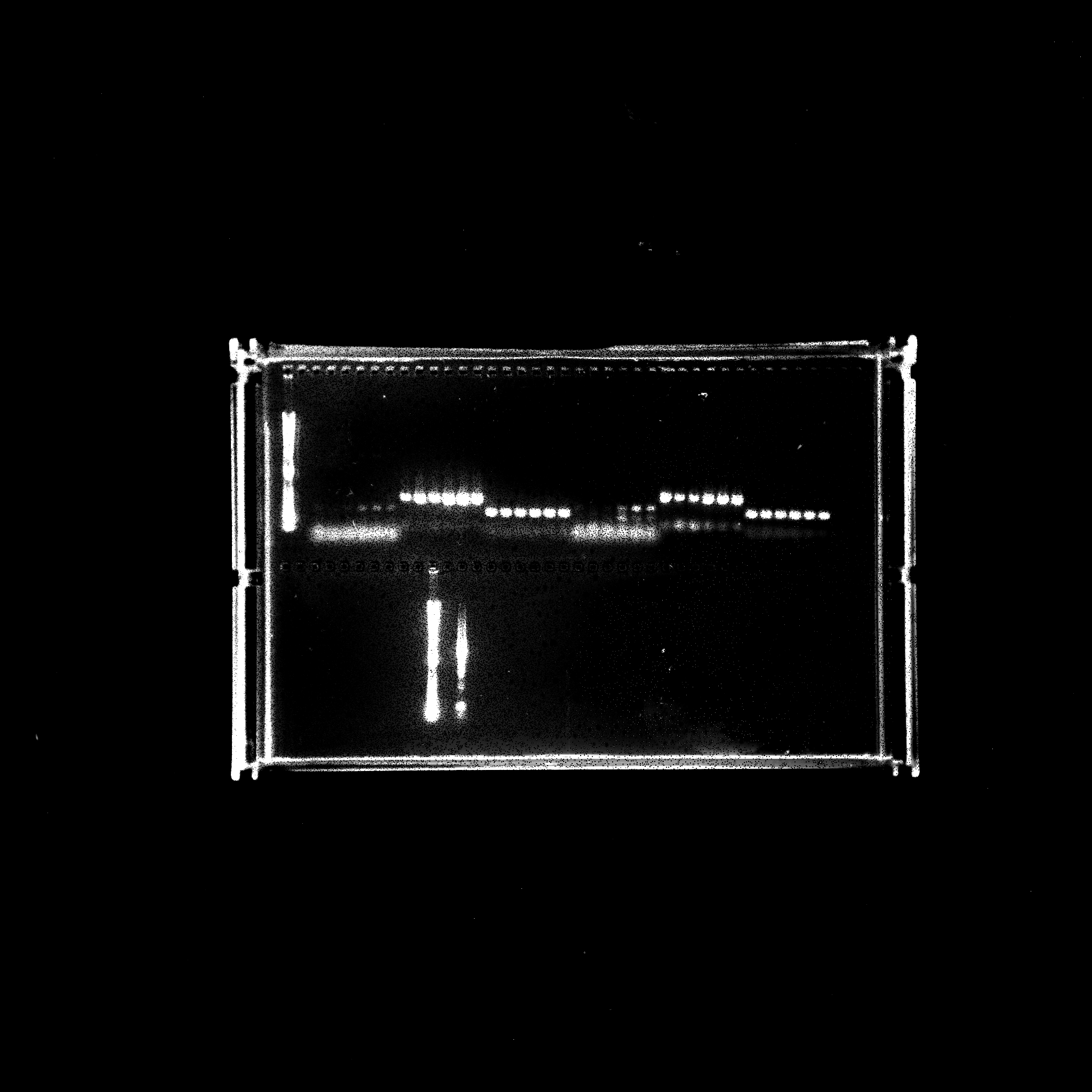

Supplement: S1 Data — Within the Data.zip file, there are 6 folders, whose name corresponds to each individual figure of our manuscript. Raw data including microscope and gel images as well as calculations are contained within each of these folders. Fig01 folder contains 2 flow cytometry plots for Figure 1A and 1C in pdf format as well as 12 phase-contrast and fluorescence microscope images for Figures 1B and 1D. Fig02 folder contains an excel sheet for cell counting and cell doubling data for Figure 2A and 2B. Fig03 folder contains 4 oil red o-stained microscope images for Figure 3A, and 1 agarose gel image (PCR data) as well as an excel sheet for calculating adipogenic gene expression data in Figure 3B. Fig04 folder contains 4 phase-contrast microscope images for Figure 4A, 6 alcian blue-stained microscope images for Figure 4B, and 2 agarose gel images (PCR data) as well as an excel sheet for calculating chondrogenic gene expression data in Figure 4C. Fig05 folder contains 4 ALP-stained microscope images for Figure 5A, an excel sheet for quantifying ALP activity in Figure 5B, 4 alizain red-stained microscope images for Figure 5C, an excel sheet for quantifying alizarin red staining in Figure 5D, and 3 agarose gel images (PCR data) as well as an excel sheet for calculating osteogenic gene expression data in Figure 5E. Fig06 folder contains 8 phase-contrast and fluorescence microscope images for Figures 6A and 6B and 2 ALP-stained images for Figure 6C. (ZIP) [file pone.0139054.s001.zip › Data/Fig05 Osteogenic Data/PCR Ocn,Osf-1-30p0s-Levels.tif]

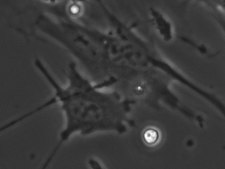

Supplement: S1 Data — Within the Data.zip file, there are 6 folders, whose name corresponds to each individual figure of our manuscript. Raw data including microscope and gel images as well as calculations are contained within each of these folders. Fig01 folder contains 2 flow cytometry plots for Figure 1A and 1C in pdf format as well as 12 phase-contrast and fluorescence microscope images for Figures 1B and 1D. Fig02 folder contains an excel sheet for cell counting and cell doubling data for Figure 2A and 2B. Fig03 folder contains 4 oil red o-stained microscope images for Figure 3A, and 1 agarose gel image (PCR data) as well as an excel sheet for calculating adipogenic gene expression data in Figure 3B. Fig04 folder contains 4 phase-contrast microscope images for Figure 4A, 6 alcian blue-stained microscope images for Figure 4B, and 2 agarose gel images (PCR data) as well as an excel sheet for calculating chondrogenic gene expression data in Figure 4C. Fig05 folder contains 4 ALP-stained microscope images for Figure 5A, an excel sheet for quantifying ALP activity in Figure 5B, 4 alizain red-stained microscope images for Figure 5C, an excel sheet for quantifying alizarin red staining in Figure 5D, and 3 agarose gel images (PCR data) as well as an excel sheet for calculating osteogenic gene expression data in Figure 5E. Fig06 folder contains 8 phase-contrast and fluorescence microscope images for Figures 6A and 6B and 2 ALP-stained images for Figure 6C. (ZIP) [file pone.0139054.s001.zip › Data/Fig06 Cell Labeling Data/Fig6A C3H01.tif]

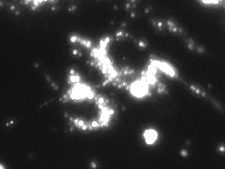

Supplement: S1 Data — Within the Data.zip file, there are 6 folders, whose name corresponds to each individual figure of our manuscript. Raw data including microscope and gel images as well as calculations are contained within each of these folders. Fig01 folder contains 2 flow cytometry plots for Figure 1A and 1C in pdf format as well as 12 phase-contrast and fluorescence microscope images for Figures 1B and 1D. Fig02 folder contains an excel sheet for cell counting and cell doubling data for Figure 2A and 2B. Fig03 folder contains 4 oil red o-stained microscope images for Figure 3A, and 1 agarose gel image (PCR data) as well as an excel sheet for calculating adipogenic gene expression data in Figure 3B. Fig04 folder contains 4 phase-contrast microscope images for Figure 4A, 6 alcian blue-stained microscope images for Figure 4B, and 2 agarose gel images (PCR data) as well as an excel sheet for calculating chondrogenic gene expression data in Figure 4C. Fig05 folder contains 4 ALP-stained microscope images for Figure 5A, an excel sheet for quantifying ALP activity in Figure 5B, 4 alizain red-stained microscope images for Figure 5C, an excel sheet for quantifying alizarin red staining in Figure 5D, and 3 agarose gel images (PCR data) as well as an excel sheet for calculating osteogenic gene expression data in Figure 5E. Fig06 folder contains 8 phase-contrast and fluorescence microscope images for Figures 6A and 6B and 2 ALP-stained images for Figure 6C. (ZIP) [file pone.0139054.s001.zip › Data/Fig06 Cell Labeling Data/Fig6A C3H02.tif]

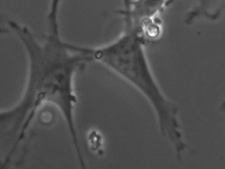

Supplement: S1 Data — Within the Data.zip file, there are 6 folders, whose name corresponds to each individual figure of our manuscript. Raw data including microscope and gel images as well as calculations are contained within each of these folders. Fig01 folder contains 2 flow cytometry plots for Figure 1A and 1C in pdf format as well as 12 phase-contrast and fluorescence microscope images for Figures 1B and 1D. Fig02 folder contains an excel sheet for cell counting and cell doubling data for Figure 2A and 2B. Fig03 folder contains 4 oil red o-stained microscope images for Figure 3A, and 1 agarose gel image (PCR data) as well as an excel sheet for calculating adipogenic gene expression data in Figure 3B. Fig04 folder contains 4 phase-contrast microscope images for Figure 4A, 6 alcian blue-stained microscope images for Figure 4B, and 2 agarose gel images (PCR data) as well as an excel sheet for calculating chondrogenic gene expression data in Figure 4C. Fig05 folder contains 4 ALP-stained microscope images for Figure 5A, an excel sheet for quantifying ALP activity in Figure 5B, 4 alizain red-stained microscope images for Figure 5C, an excel sheet for quantifying alizarin red staining in Figure 5D, and 3 agarose gel images (PCR data) as well as an excel sheet for calculating osteogenic gene expression data in Figure 5E. Fig06 folder contains 8 phase-contrast and fluorescence microscope images for Figures 6A and 6B and 2 ALP-stained images for Figure 6C. (ZIP) [file pone.0139054.s001.zip › Data/Fig06 Cell Labeling Data/Fig6A mRuby01.TIF]

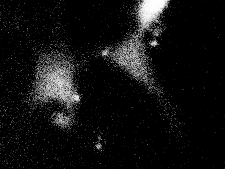

Supplement: S1 Data — Within the Data.zip file, there are 6 folders, whose name corresponds to each individual figure of our manuscript. Raw data including microscope and gel images as well as calculations are contained within each of these folders. Fig01 folder contains 2 flow cytometry plots for Figure 1A and 1C in pdf format as well as 12 phase-contrast and fluorescence microscope images for Figures 1B and 1D. Fig02 folder contains an excel sheet for cell counting and cell doubling data for Figure 2A and 2B. Fig03 folder contains 4 oil red o-stained microscope images for Figure 3A, and 1 agarose gel image (PCR data) as well as an excel sheet for calculating adipogenic gene expression data in Figure 3B. Fig04 folder contains 4 phase-contrast microscope images for Figure 4A, 6 alcian blue-stained microscope images for Figure 4B, and 2 agarose gel images (PCR data) as well as an excel sheet for calculating chondrogenic gene expression data in Figure 4C. Fig05 folder contains 4 ALP-stained microscope images for Figure 5A, an excel sheet for quantifying ALP activity in Figure 5B, 4 alizain red-stained microscope images for Figure 5C, an excel sheet for quantifying alizarin red staining in Figure 5D, and 3 agarose gel images (PCR data) as well as an excel sheet for calculating osteogenic gene expression data in Figure 5E. Fig06 folder contains 8 phase-contrast and fluorescence microscope images for Figures 6A and 6B and 2 ALP-stained images for Figure 6C. (ZIP) [file pone.0139054.s001.zip › Data/Fig06 Cell Labeling Data/Fig6A mRuby02.tif]

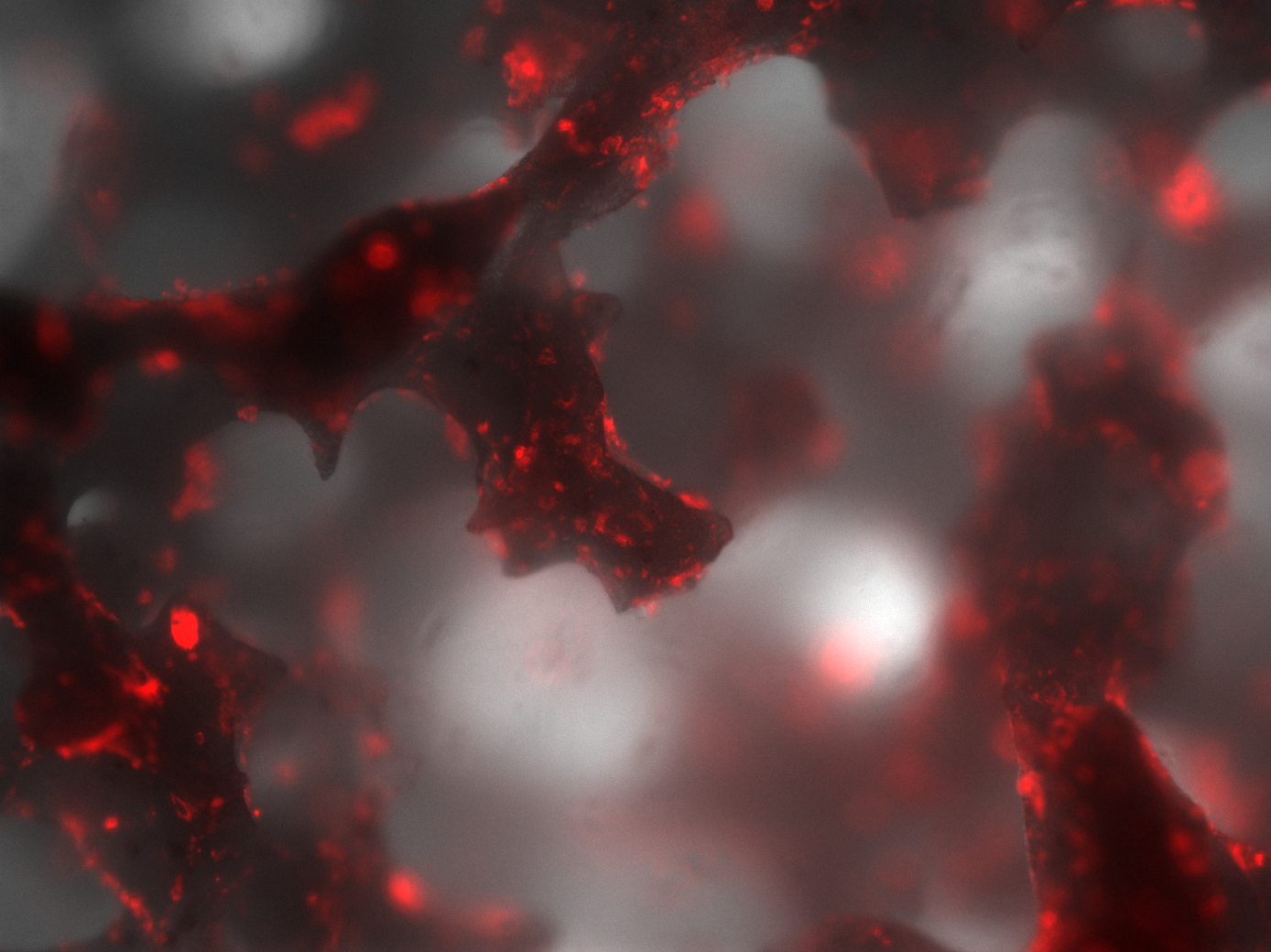

Supplement: S1 Data — Within the Data.zip file, there are 6 folders, whose name corresponds to each individual figure of our manuscript. Raw data including microscope and gel images as well as calculations are contained within each of these folders. Fig01 folder contains 2 flow cytometry plots for Figure 1A and 1C in pdf format as well as 12 phase-contrast and fluorescence microscope images for Figures 1B and 1D. Fig02 folder contains an excel sheet for cell counting and cell doubling data for Figure 2A and 2B. Fig03 folder contains 4 oil red o-stained microscope images for Figure 3A, and 1 agarose gel image (PCR data) as well as an excel sheet for calculating adipogenic gene expression data in Figure 3B. Fig04 folder contains 4 phase-contrast microscope images for Figure 4A, 6 alcian blue-stained microscope images for Figure 4B, and 2 agarose gel images (PCR data) as well as an excel sheet for calculating chondrogenic gene expression data in Figure 4C. Fig05 folder contains 4 ALP-stained microscope images for Figure 5A, an excel sheet for quantifying ALP activity in Figure 5B, 4 alizain red-stained microscope images for Figure 5C, an excel sheet for quantifying alizarin red staining in Figure 5D, and 3 agarose gel images (PCR data) as well as an excel sheet for calculating osteogenic gene expression data in Figure 5E. Fig06 folder contains 8 phase-contrast and fluorescence microscope images for Figures 6A and 6B and 2 ALP-stained images for Figure 6C. (ZIP) [file pone.0139054.s001.zip › Data/Fig06 Cell Labeling Data/Fig6B 01c-05X-CeramicC3H10THalfP12-5uMDiI-Day02-01_c1+2.tif]

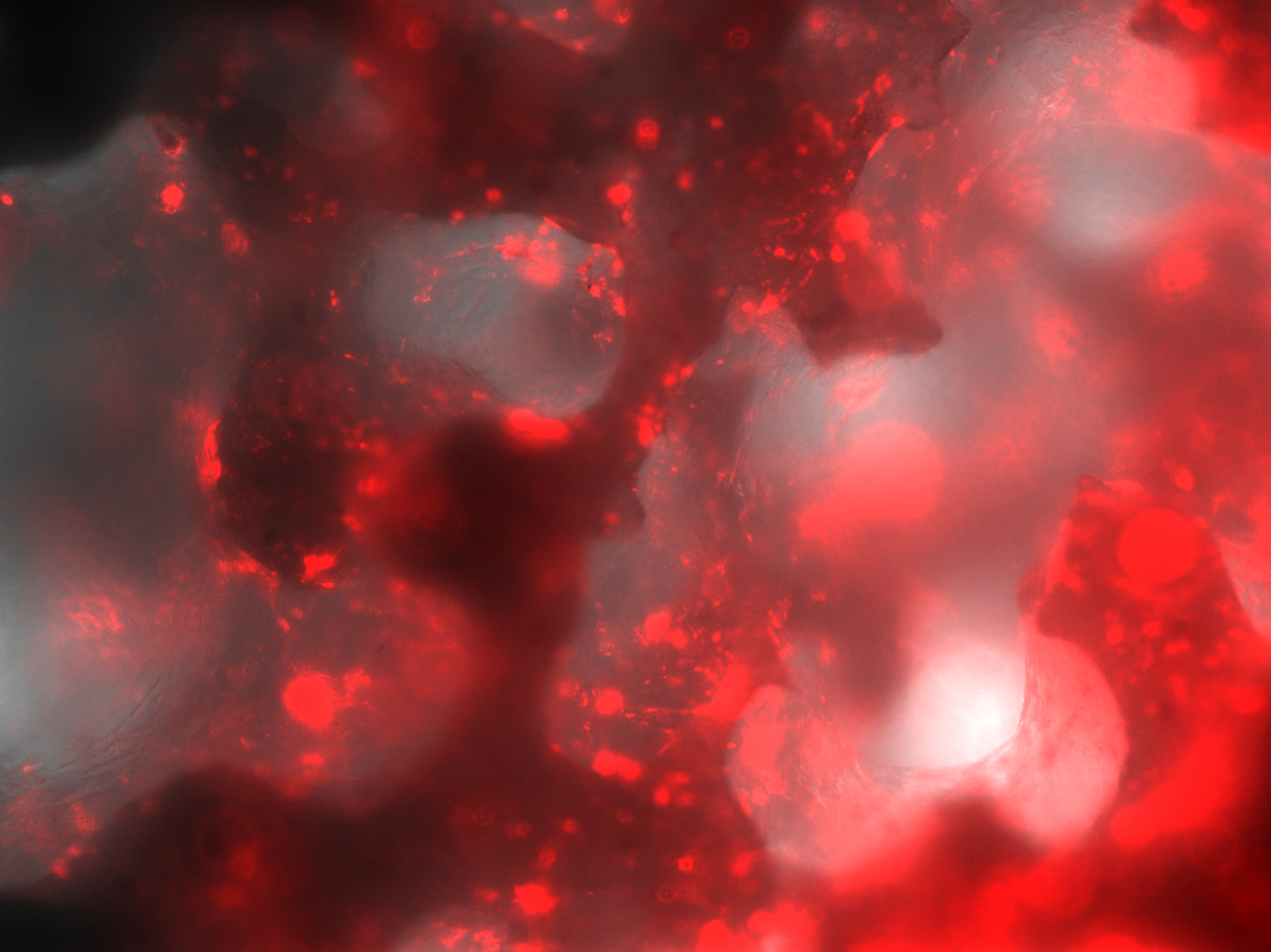

Supplement: S1 Data — Within the Data.zip file, there are 6 folders, whose name corresponds to each individual figure of our manuscript. Raw data including microscope and gel images as well as calculations are contained within each of these folders. Fig01 folder contains 2 flow cytometry plots for Figure 1A and 1C in pdf format as well as 12 phase-contrast and fluorescence microscope images for Figures 1B and 1D. Fig02 folder contains an excel sheet for cell counting and cell doubling data for Figure 2A and 2B. Fig03 folder contains 4 oil red o-stained microscope images for Figure 3A, and 1 agarose gel image (PCR data) as well as an excel sheet for calculating adipogenic gene expression data in Figure 3B. Fig04 folder contains 4 phase-contrast microscope images for Figure 4A, 6 alcian blue-stained microscope images for Figure 4B, and 2 agarose gel images (PCR data) as well as an excel sheet for calculating chondrogenic gene expression data in Figure 4C. Fig05 folder contains 4 ALP-stained microscope images for Figure 5A, an excel sheet for quantifying ALP activity in Figure 5B, 4 alizain red-stained microscope images for Figure 5C, an excel sheet for quantifying alizarin red staining in Figure 5D, and 3 agarose gel images (PCR data) as well as an excel sheet for calculating osteogenic gene expression data in Figure 5E. Fig06 folder contains 8 phase-contrast and fluorescence microscope images for Figures 6A and 6B and 2 ALP-stained images for Figure 6C. (ZIP) [file pone.0139054.s001.zip › Data/Fig06 Cell Labeling Data/Fig6B 01f-05X-CeramicC3H10THalfP12-5uMDiI-Day10-02_c1+2.tif]

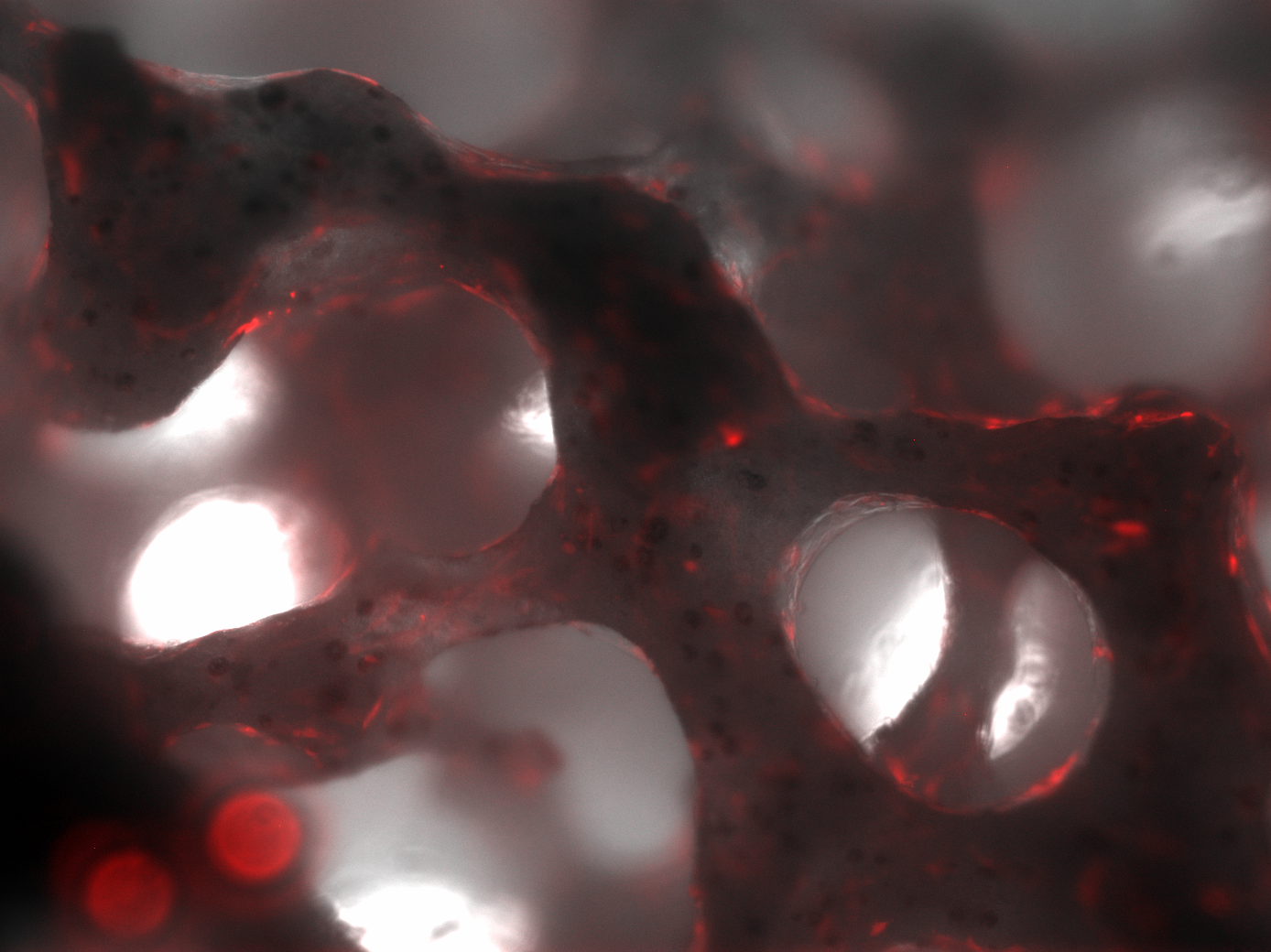

Supplement: S1 Data — Within the Data.zip file, there are 6 folders, whose name corresponds to each individual figure of our manuscript. Raw data including microscope and gel images as well as calculations are contained within each of these folders. Fig01 folder contains 2 flow cytometry plots for Figure 1A and 1C in pdf format as well as 12 phase-contrast and fluorescence microscope images for Figures 1B and 1D. Fig02 folder contains an excel sheet for cell counting and cell doubling data for Figure 2A and 2B. Fig03 folder contains 4 oil red o-stained microscope images for Figure 3A, and 1 agarose gel image (PCR data) as well as an excel sheet for calculating adipogenic gene expression data in Figure 3B. Fig04 folder contains 4 phase-contrast microscope images for Figure 4A, 6 alcian blue-stained microscope images for Figure 4B, and 2 agarose gel images (PCR data) as well as an excel sheet for calculating chondrogenic gene expression data in Figure 4C. Fig05 folder contains 4 ALP-stained microscope images for Figure 5A, an excel sheet for quantifying ALP activity in Figure 5B, 4 alizain red-stained microscope images for Figure 5C, an excel sheet for quantifying alizarin red staining in Figure 5D, and 3 agarose gel images (PCR data) as well as an excel sheet for calculating osteogenic gene expression data in Figure 5E. Fig06 folder contains 8 phase-contrast and fluorescence microscope images for Figures 6A and 6B and 2 ALP-stained images for Figure 6C. (ZIP) [file pone.0139054.s001.zip › Data/Fig06 Cell Labeling Data/Fig6B 02c-05X-CeramicC3H10THalfP12mRuby2-Day02-03_c1+2.tif]

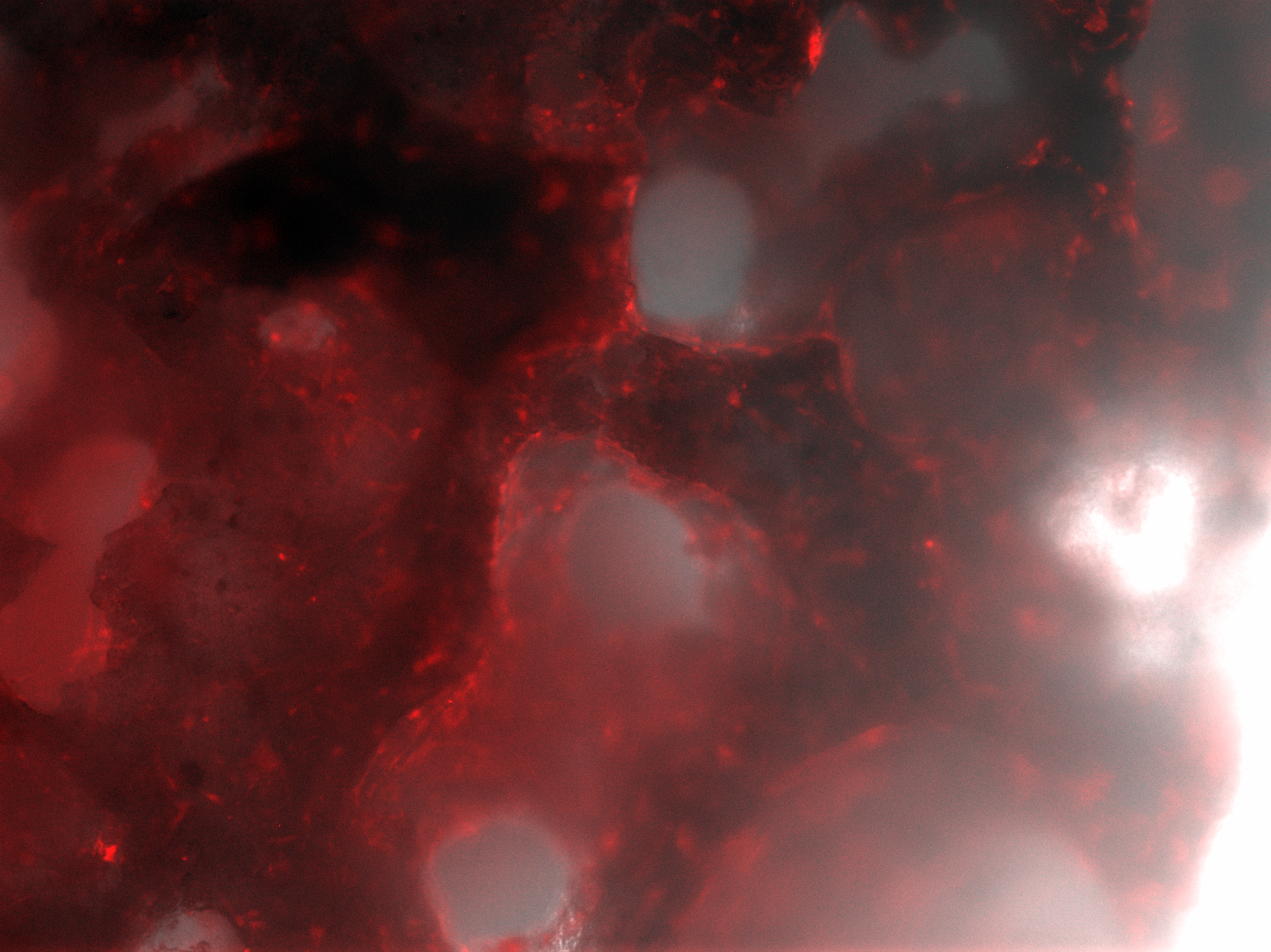

Supplement: S1 Data — Within the Data.zip file, there are 6 folders, whose name corresponds to each individual figure of our manuscript. Raw data including microscope and gel images as well as calculations are contained within each of these folders. Fig01 folder contains 2 flow cytometry plots for Figure 1A and 1C in pdf format as well as 12 phase-contrast and fluorescence microscope images for Figures 1B and 1D. Fig02 folder contains an excel sheet for cell counting and cell doubling data for Figure 2A and 2B. Fig03 folder contains 4 oil red o-stained microscope images for Figure 3A, and 1 agarose gel image (PCR data) as well as an excel sheet for calculating adipogenic gene expression data in Figure 3B. Fig04 folder contains 4 phase-contrast microscope images for Figure 4A, 6 alcian blue-stained microscope images for Figure 4B, and 2 agarose gel images (PCR data) as well as an excel sheet for calculating chondrogenic gene expression data in Figure 4C. Fig05 folder contains 4 ALP-stained microscope images for Figure 5A, an excel sheet for quantifying ALP activity in Figure 5B, 4 alizain red-stained microscope images for Figure 5C, an excel sheet for quantifying alizarin red staining in Figure 5D, and 3 agarose gel images (PCR data) as well as an excel sheet for calculating osteogenic gene expression data in Figure 5E. Fig06 folder contains 8 phase-contrast and fluorescence microscope images for Figures 6A and 6B and 2 ALP-stained images for Figure 6C. (ZIP) [file pone.0139054.s001.zip › Data/Fig06 Cell Labeling Data/Fig6B 02f-05X-CeramicC3H10THalfP12mRuby2-Day10-02_c1+2.tif]

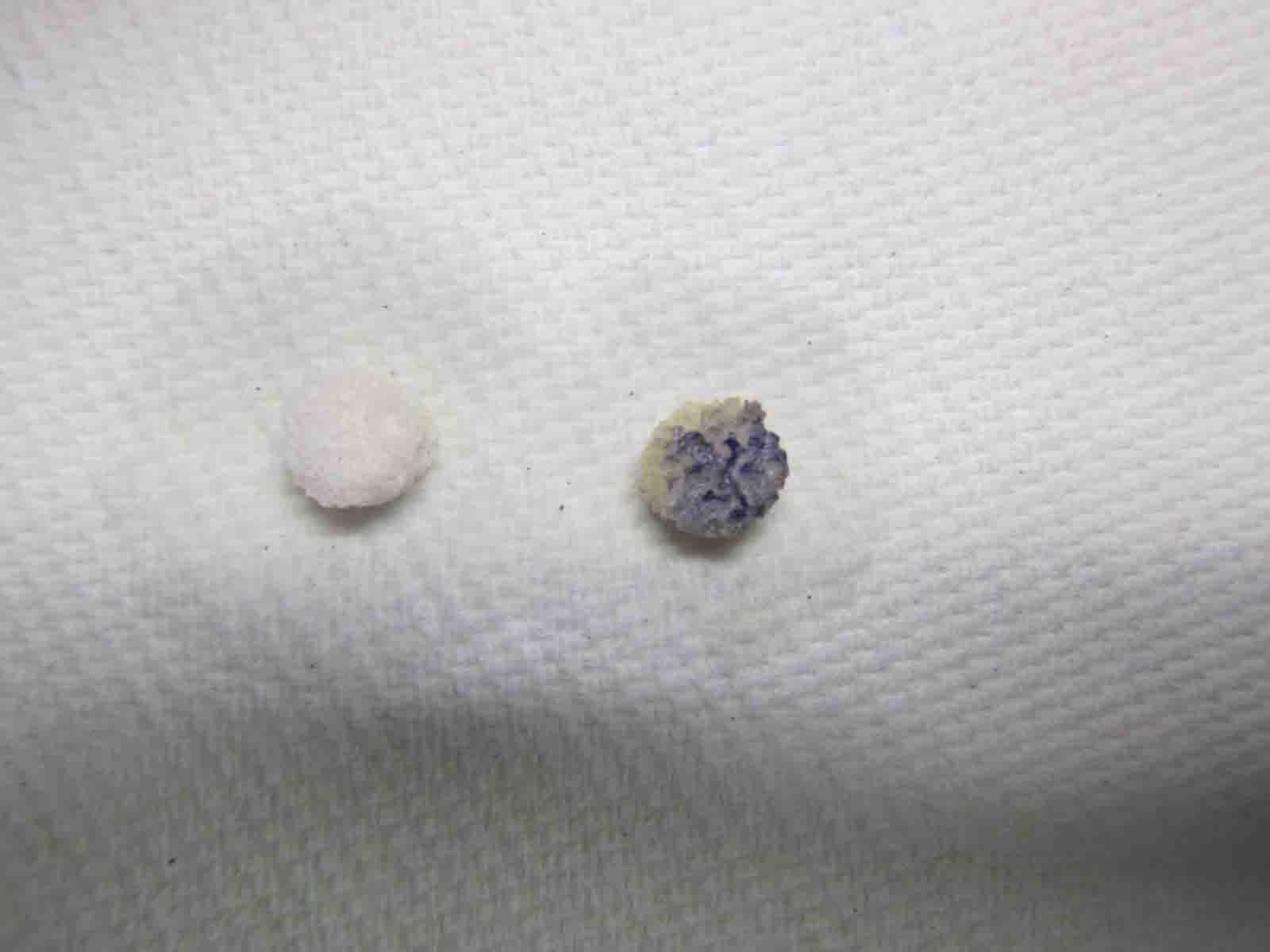

Supplement: S1 Data — Within the Data.zip file, there are 6 folders, whose name corresponds to each individual figure of our manuscript. Raw data including microscope and gel images as well as calculations are contained within each of these folders. Fig01 folder contains 2 flow cytometry plots for Figure 1A and 1C in pdf format as well as 12 phase-contrast and fluorescence microscope images for Figures 1B and 1D. Fig02 folder contains an excel sheet for cell counting and cell doubling data for Figure 2A and 2B. Fig03 folder contains 4 oil red o-stained microscope images for Figure 3A, and 1 agarose gel image (PCR data) as well as an excel sheet for calculating adipogenic gene expression data in Figure 3B. Fig04 folder contains 4 phase-contrast microscope images for Figure 4A, 6 alcian blue-stained microscope images for Figure 4B, and 2 agarose gel images (PCR data) as well as an excel sheet for calculating chondrogenic gene expression data in Figure 4C. Fig05 folder contains 4 ALP-stained microscope images for Figure 5A, an excel sheet for quantifying ALP activity in Figure 5B, 4 alizain red-stained microscope images for Figure 5C, an excel sheet for quantifying alizarin red staining in Figure 5D, and 3 agarose gel images (PCR data) as well as an excel sheet for calculating osteogenic gene expression data in Figure 5E. Fig06 folder contains 8 phase-contrast and fluorescence microscope images for Figures 6A and 6B and 2 ALP-stained images for Figure 6C. (ZIP) [file pone.0139054.s001.zip › Data/Fig06 Cell Labeling Data/Fig6C IMG_0763-mRuby2.JPG]

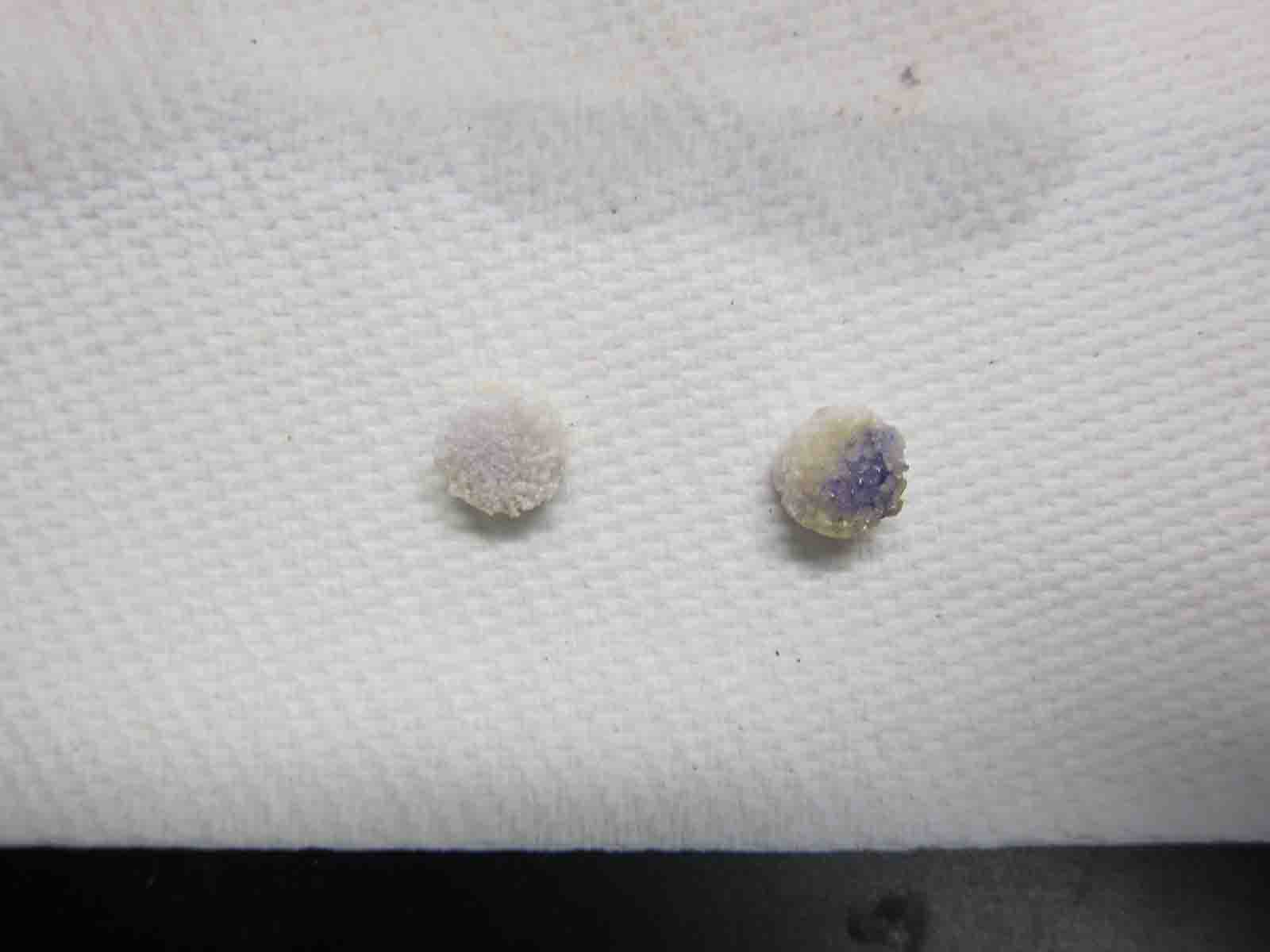

Supplement: S1 Data — Within the Data.zip file, there are 6 folders, whose name corresponds to each individual figure of our manuscript. Raw data including microscope and gel images as well as calculations are contained within each of these folders. Fig01 folder contains 2 flow cytometry plots for Figure 1A and 1C in pdf format as well as 12 phase-contrast and fluorescence microscope images for Figures 1B and 1D. Fig02 folder contains an excel sheet for cell counting and cell doubling data for Figure 2A and 2B. Fig03 folder contains 4 oil red o-stained microscope images for Figure 3A, and 1 agarose gel image (PCR data) as well as an excel sheet for calculating adipogenic gene expression data in Figure 3B. Fig04 folder contains 4 phase-contrast microscope images for Figure 4A, 6 alcian blue-stained microscope images for Figure 4B, and 2 agarose gel images (PCR data) as well as an excel sheet for calculating chondrogenic gene expression data in Figure 4C. Fig05 folder contains 4 ALP-stained microscope images for Figure 5A, an excel sheet for quantifying ALP activity in Figure 5B, 4 alizain red-stained microscope images for Figure 5C, an excel sheet for quantifying alizarin red staining in Figure 5D, and 3 agarose gel images (PCR data) as well as an excel sheet for calculating osteogenic gene expression data in Figure 5E. Fig06 folder contains 8 phase-contrast and fluorescence microscope images for Figures 6A and 6B and 2 ALP-stained images for Figure 6C. (ZIP) [file pone.0139054.s001.zip › Data/Fig06 Cell Labeling Data/Fig6C IMG_0765-DilCLabelled.JPG]
